# Boring systematics: A genome skimmed phylogeny of ctenostome bryozoans and their endolithic family Penetrantiidae with the description of one new species

**DOI:** 10.1002/ece3.11276

**Published:** 2024-04-18

**Authors:** Sebastian H. Decker, Ahmed J. Saadi, Christian Baranyi, Masato Hirose, Sarah Lemer, Andy Sombke, Felipe Aguilera, Leandro M. Vieira, Abigail M. Smith, Andrea Waeschenbach, Thomas Schwaha

**Affiliations:** ^1^ Department of Evolutionary Biology University of Vienna Vienna Austria; ^2^ School of Marine Biosciences Kitasato University Minato‐ku Japan; ^3^ Marine Laboratory UOG Station Mangilao Guam USA; ^4^ Center for Anatomy and Cell Biology, Cell and Developmental Biology Medical University of Vienna Vienna Austria; ^5^ Departamento de Bioquímica y Biología Molecular, Facultad de Ciencias Biológicas Universidad de Concepción Concepción Chile; ^6^ Laboratório de Estudos de Bryozoa—LAEBry, Departamento de Zoologia, Centro de Biociências Universidade Federal de Pernambuco Recife PE Brazil; ^7^ Department of Life Science Natural History Museum London UK; ^8^ Department of Marine Science University of Otago Dunedin New Zealand

**Keywords:** cryptic, mitogenomes, Multiporata, *Penetrantia*, skimming, *Terebripora*

## Abstract

Ctenostomes are a group of gymnolaemate bryozoans with an uncalcified chitinous body wall having few external, skeletal characters. Hence, species identification is challenging and their systematics remain poorly understood, even more so when they exhibit an endolithic (boring) lifestyle. Currently, there are four Recent families of endolithic bryozoans that live inside mineralized substrates like mollusk shells. In particular, Penetrantiidae Silén, 1946 has received considerable attention and its systematic affinity to either cheilostomes or ctenostomes has been debated. Species delimitation of penetrantiids remains difficult, owing to a high degree of colonial and zooidal plasticity. Consequently, an additional molecular approach is essential to unravel the systematics of penetrantiids, their phylogenetic placement and their species diversity. We therefore sequenced the mitochondrial (mt) genomes and two nuclear markers of 27 ctenostome species including nine penetrantiids. Our phylogeny supports the Penetrantiidae as a monophyletic group placed as sister taxon to the remaining ctenostomes alongside paludicellids, arachnidioids and terebriporids. The boring family Terebriporidae d'Orbigny, 1847 were previously considered to be among vesicularioids, but our results suggest an arachnidioid affinity instead. Ctenostome paraphyly is supported by our data, as the cheilostomes nest within them. A Multiporata clade is also well supported, including the former victorelloid genus *Sundanella*. Altogether, this study provides new insights into ctenostome systematics, assists with species delimitation and contributes to our understanding of the bryozoan tree of life.

## Graphical abstract

Better resolving ctenostome bryozoan systematics is crucial in understanding bryozoan evolution in general. Ctenostomes are a paraphyletic group of soft bodied bryozoans with a wide ecological diversity, including endolithic (boring) forms like *Penetrantia*. Consequently, this study seeks for a larger phylogenetic analysis based on data from mitochondrial genomes and nuclear ribosomal RNA.

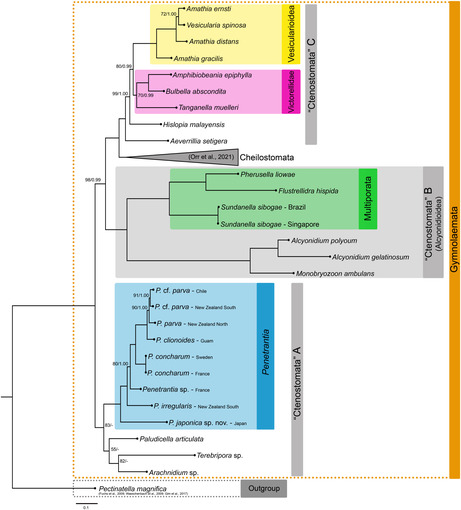

## 1 INTRODUCTION

Bryozoa is a clade of sessile and filter feeding metazoans that occurs in marine and freshwater habitats. As epibenthic organisms, they colonize various hard substrates and can create colonies up to several centimeters in size, which are composed of individual units (zooids) (Ryland, 1970; Schwaha, 2020a). Zooids that contain a tentacle crown (lophophore and digestive system) and actively feed are called autozooids, while polymorphic zooids specialized for reproduction, defense or colonial connectivity are called heterozooids (Mukai et al., 1997; Schack et al., 2019). The majority of the currently known extant ~6000 bryozoan species (Bock & Gordon, 2013) thrive in marine environments from intertidal to subtidal regimes and to depths of more than 7000 m (Grischenko et al., 2019; Ryland, 1970). The largest and most diverse taxon, Gymnolaemata, is subdivided into Cheilostomata and “Ctenostomata.” Cheilostomes comprise about 5000 known Recent species, while ctenostomes account for approximately 350 described species (Bock & Gordon, 2013). Cheilostomes have an (at least) partially calcified body wall that exhibits many skeletal features suitable for species identification, while ctenostomes lack such features (Schwaha, 2020b, 2020c, 2020d). Ctenostomes remain widely understudied, although they are important for our understanding of the interrelationships and evolution of gymnolaemates. It is presumed that cheilostomes originated from ctenostome‐like ancestors, rendering the latter paraphyletic (Jebram, 1973; Schwaha, 2020c; Todd, 2000; Waeschenbach et al., 2012).

Traditionally, ctenostomes were classified into eight superfamilies (Benedeniporoidea, Alcyonidioidea, Arachnidioidea, Hislopioidea, Paludicelloidea, Vesicularioidea, Victorelloidea and Walkerioidea) (Jebram, 1973, 1986; Todd, 2000), but the monophyly of some superfamilies remains uncertain. Ctenostomes have a wide ecological diversity living in marine, brackish and freshwater habitats and evolved specialized lifestyles, e.g., endolithic or solitary (Ryland, 1970; Schwaha, 2020c). Endolithic (boring) ctenostomes have received a considerable amount of attention thanks to their long fossil record, which dates back to the Ordovician (Pohowsky, 1978). However, their ecology and phylogeny remain poorly understood (Schwaha, 2020c). Endolithic bryozoans live immersed in calcareous substrates such as mollusk shells. They excavate their cavities by means of chemical dissolution with only minute boring traces of about 50–100 μm visible from the outside (Pohowsky, 1978). There are four Recent endolithic ctenostome families currently recognized: Terebriporidae d'Orbigny, 1847, Spathiporidae Pohowsky, 1978, Immergentiidae Silén, 1946 and Penetrantiidae Silén, 1946 (Pohowsky, 1978; Schwaha, 2020c). Of special interest is the boring family Penetrantiidae, with its sole genus *Penetrantia* including ten Recent species (Pohowsky, 1978; Silén, 1946, 1947). Penetrantiids feature an operculum and brood chambers similar to cheilostomes, but have an uncalcified body wall and stolonate colonies commonly found in ctenostomes (Pohowsky, 1978; Silén, 1947; Soule & Soule, 1969). They were judged to be associated with cheilostomes or ctenostomes, the subject of considerable debate (Pohowsky, 1978; Smyth, 1988; Soule & Soule, 1969). Several morphological investigations suggested convergent evolution of these cheilostome‐like features in penetrantiids, placing them firmly among ctenostomes (Decker et al., 2023; Pohowsky, 1978; Schwaha, 2020c). However, uncertainty remains concerning their classification and phylogenetic position. Based on the presence of polymorphic stolons, a close relationship with the two other ctenostome superfamilies that feature true stolons, Vesicularioidea and Walkerioidea, was suggested (Hayward, 1985; Pohowsky, 1978; Schwaha, 2020c).

Because only a few external characters can be detected, correct species identification of penetrantiids remains challenging and some species were described based on their boring traces alone. Therefore, thorough histological investigations are necessary to successfully delimitate penetrantiid species (Decker et al., 2023).

Although the number of molecular phylogenetic studies on bryozoans has increased in recent years, most of them focused on cheilostome bryozoans, with only a few studies included ctenostome representatives (Fuchs et al., 2009; Orr et al., 2019, 2021, 2022; Waeschenbach et al., 2012, 2015). The most comprehensive ctenostome molecular phylogenies are based on a handful of genes but play a crucial role in our understanding of ctenostome systematics and support their paraphyly (Waeschenbach et al., 2012, 2015). Consequently, this study seeks for a larger phylogenetic analysis based on data from mitochondrial (mt) genomes and nuclear ribosomal RNA (rRNA) genes 18S and 28S. Our analysis includes 27 ctenostome species representing seven of their eight superfamilies. The molecular phylogenetic framework is combined with known morphological characters to shed light on (1) general ctenostome phylogeny, (2) the systematic position of the Penetrantiidae and (3) the interrelationships of Penetrantiidae. Additionally, this study aims to unravel potential cryptic species complexes in the genus *Penetrantia* by including specimens from ten different geographical regions. Furthermore, we include one species of the boring ctenostome family Terebriporidae to evaluate whether an endolithic lifestyle evolved independently within ctenostomes. As this is one of the first studies comprising such a large molecular dataset of ctenostome bryozoans, it will also contribute to future analysis on the systematics of Gymnolaemata and might help to resolve the origin of cheilostomes and the paraphyly of ctenostomes.

## 2 MATERIALS AND METHODS

### 2.1 Sample collection and imaging

Twenty‐seven specimens from 11 different localities were collected for genome skimming including nine different *Penetrantia* and 18 additional ctenostome specimens (Table 1). One additional penetrantiid specimen (*Penetrantia* sp.) was collected in Helgoland, Germany (54°08.339′ N 7°52.298′ E) for sanger sequencing of the cytochrome *c* oxidase subunit I (cox1) gene (OR632352) and genetic distance analysis only (see below). Samples were either collected in the intertidal zone by hand or in shallow subtidal areas by dredging. All samples were fixed either in 96% or absolute ethanol and stored at 4°C until further investigation. Stereomicroscopic pictures were taken with a Nikon SMZ25 stereomicroscope (Nikon, Tokyo, Japan) equipped with a DsRi2 microscope camera, or with a Hirox RH‐2000 3D digital microscope (Hirox Co., Ltd., Tokyo, Japan). Scanning electron microscopic images were generated using a JEOL IT 300 (JEOL, Akishima, Tokyo, Japan) with a secondary detector at 10–25 KeV.

**TABLE 1:** Sample details and accession numbers of specimens used for genome skimming in this study.

Species	Collected	Location		Genes	GenBank accession number				
					mt genome	COX1	18S	28S	Raw (SRA)
*Arachnidium* sp.	2022	Stolvezen, Roscoff, France	48°42.847′ N 03°53.5′ W	17	OR620116	—	OR625474	OR625501	SRR28306760
*Terebripora* sp.	2022	Caleta Chome, Chile	36°46.387′ S 73°12.698′ W	17	OR620138	OR632416	OR625496	OR625523	SRR28306759
*Paludicella articulata* (Ehrenberg, 1831)	2022	Laxenburg, Austria	48°03.943′ N 16°22.140′ E	17	OR620117	OR632404	OR625475	OR625502	SRR28306746
*Penetrantia parva* Silén, 1946	2019	SW of Motukawanui Island, New Zealand	35°00.347′ S 173°55.337′ E	17	OR620118	OR632344	OR625476	OR625503	SRR28306740
*Penetrantia* cf. *parva*	2021	Dunedin, New Zealand	45°46.77′ S 170°57.40′ E	17	OR620119	OR632345	OR625477	OR625504	SRR28306739
*Penetrantia* cf. *parva*	2022	Caleta Chome, Chile	36°46.387′ S 73°12.698′ W	17	OR620120	OR632346	OR625478	OR625505	SRR28306738
*Penetrantia clionoides* Smyth, 1988	2020	Pago Bay, Guam	13°25.655′ N 144°47.890′ E	17	OR620121	OR632347	OR625479	OR625506	SRR28306737
*Penetrantia irregularis* Silén, 1956	2021	Dunedin, New Zealand	45°46.77′ S 170°57.40′ E	17	OR620122	OR632348	OR625480	OR625507	SRR28306736
*Penetrantia concharum* Silén, 1946	2019	Kristineberg, Sweden	58°12.96′ N 11°24.48′ E	17	OR620123	OR632349	OR625481	OR625508	SRR28306735
*Penetrantia concharum* Silén, 1946	2020	Chateau du Taureau, Roscoff, France	48°40.2’′ N 3°53.12′ W	17	OR620124	—	OR625482	OR625509	SRR28306734
*Penetrantia* sp.	2021	Stolvezen, Roscoff, France	48°42.847′ N 3°53.5′ W	17	OR620125	OR632350	OR625483	OR625510	SRR28372652
*Penetrantia japonica* sp. nov.	2020	Tenjin‐Jima Island, Sagami Bay, Japan	35°13.336′ N 139°36.152′ E	16	OR620126	OR632351	OR625484	OR625511	SRR28306757
*Alcyonidium polyoum* (Hassall, 1841)	2022	Stolvezen, Roscoff, France	48°42.847′ N 03°53.5′ W	16	OR620127	OR632405	OR625485	OR625512	SRR28306756
*Alcyonidium gelatinosum* (Linnaeus, 1761)	2022	Stolvezen, Roscoff, France	48°42.847′ N 03°53.5′ W	16	OR620128	OR632406	OR625486	OR625513	SRR28306755
*Pherusella liowae* Decker, Gordon, Spencer Jones & Schwaha, 2021	2019	Pulau Ubin, Singapore	1°24.171′ N 103°58.380′ E	16	OR620129	OR632407	OR625487	OR625514	SRR28306754
*Flustrellidra hispida* (Fabricius, 1780)	2022	Stolvezen, Roscoff, France	48°42.847′ N 03°53.5’′ W	17	OR620130	OR632408	OR625488	OR625515	SRR28306753
*Sundanella sibogae* (Harmer, 1915)	2019	Singapore	1°26.748′ N 103°42.480′ E	17	OR620131	OR632409	OR625489	OR625516	SRR28306752
*Sundanella sibogae* (Harmer, 1915)	2022	Barra de Catuama, Goiana, Pernambuco, Brazil	7°39.300′ S 34°49.450′ W	17	OR620132	OR632410	OR625490	OR625517	SRR28306751
*Aeverrillia setigera* (Hincks, 1887)	2022	Barra de Catuama, Goiana, Pernambuco, Brazil	7°39.300′ S 34°49.450′ W	17	OR620133	OR632411	OR625491	OR625518	SRR28306750
*Hislopia malayensis* Annandale, 1916	2020	Bangkok, Thailand	—	17	OR620134	OR632412	OR625492	OR625519	SRR28306749
*Tanganella muelleri* Kraepelin, 1887	2022	Greifswald, Germany	54°5.953′ N 13°24.105′ E	16	OR620135	OR632413	OR625493	OR625520	SRR28306747
*Bulbella abscondita* Braem, 1951	2022	Greifswald, Germany	54° 5.953′ N 13° 24.105′ E	17	OR620136	OR632414	OR625494	OR625521	SRR28306746
*Amphibiobeania epiphylla* Metcalfe, Gordon & Hayward, 2007	2007	Darwin, Australia	—	17	OR620137	OR632415	OR625495	OR625522	SRR28306745
*Amathia gracilis* (Leidy, 1855)	2022	Greifswald, Germany	54°5.953′ N 13°24.105′ E	17	OR620139	—	OR625497	OR625524	SRR28306744
*Amathia distans* Busk, 1886	2022	Barra de Catuama, Goiana, Pernambuco, Brazil	7°39.300′ S 34°49.450′ W	17	OR620140	OR632417	OR625498	OR625525	SRR28306743
*Amathia ernsti* (Vieira, Migotto & Winston, 2014)	2022	Barra de Catuama, Goiana, Pernambuco, Brazil	7°39.300′ S 34°49.450′ W	16	OR620141	OR632418	OR625499	OR625526	SRR28306742
*Vesicularia spinosa* (Linnaeus, 1758)	2022	Stolvezen, Roscoff, France	48°42.847′ N 03°53.5′ W	16	OR620142	OR632419	OR625500	OR625527	SRR28306741

### 2.2 DNA extraction

Genomic DNA (gDNA) of all samples was extracted using the QIAamp DNA Micro Kit (QIAGEN, Hilden, Germany) following the manufacturer's guidelines. Specimens of the endolithic genera *Penetrantia* and *Terebripora* were removed from their calcareous substrate either by mechanical breakage or by dissolving the substrate with 20% ethylenediaminetetraacetic acid (EDTA).

### 2.3 PCR amplification, sequencing and cox1 gene sequence analysis

Prior to genome skimming, the cox1 gene was sequenced for each specimen using PCR and Sanger sequencing. PCR amplification used universal (Folmer et al., 1994) or specific bryozoan primers (Table A1). PCR reactions were performed in 30 μL reaction volumes with 1 μL of 20 μM of each primer, 1–3 μL of gDNA and 15 μL of Red HS Taq Master Mix (Biozym, Oldendorf, Germany). PCR products were cleaned using an enzymatic cleanup reagent A'SAP (ArcticZymes Technologies ASA, Tromsø, Norway) and sent to Microsynth Austria GmbH for sequencing. Chromatograms were edited with SeaView v5.0.5 (Gouy et al., 2010) and aligned with MAFFT v7.520 using the model L‐INS‐i (Katoh et al., 2002, 2005).

### 2.4 Illumina sequencing, assembly and annotation

Library preparation and sequencing were conducted by the Next‐Generation Sequencing Facility at the Vienna BioCenter Core Facilities (VBCF). Genomic DNA libraries were constructed using NEBNext® Ultra™ II FS DNA Library Prep Kit for Illumina, with inputs >100 ng (# E7805). Multiplexing was done using the NEBNext Multiplex Oligos for Illumina (Dual Index Primers, NEB #E7600). Libraries were sequenced on an Illumina NextSeq 550 platform using the 300 Cycle Mid Output mode.

**FIGURE A1:**
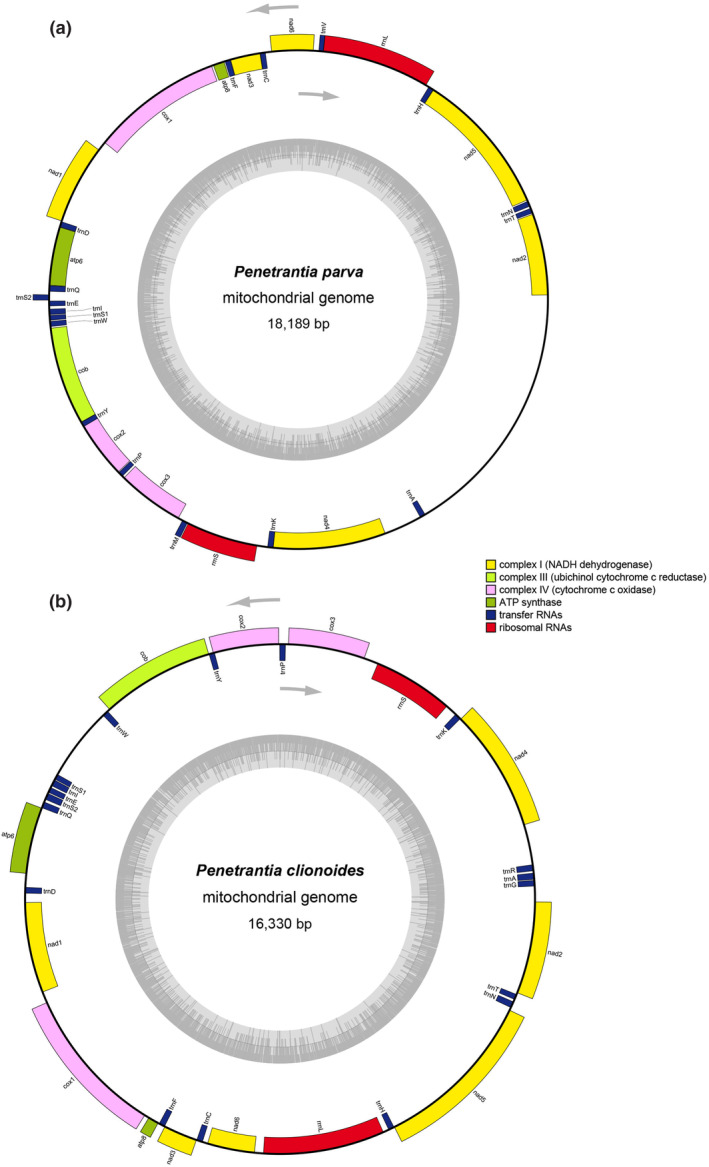
Circularized mitochondrial genome map of *Penetrantia parva* from northern New Zealand (a) and *Penetrantia clionoides* from Guam (b). Arrows show direction of transcription with outer strand corresponding to the forward and the inner to the reverse strand.

Prior to assembly, raw Illumina reads were quality‐checked with FastQC v0.11.8 (www.bioinformatics.babraham.ac.uk/projects/fastqc; last accessed April 08, 2022) and trimmed of adapters and low‐quality sequences using Trim Galore v0.6.5 (https://github.com/FelixKrueger/TrimGalore; last accessed April 08, 2022) with default setting. The clean reads were de novo assembled using SPAdes v3.15.3 (Bankevich et al., 2012) with k‐mers of 21, 33, 55, 77, 99 and 127. Mt genome contigs were identified using BLASTN (Altschul et al., 1990) and annotated with the MITOS2 web server (Donath et al., 2019) using the metazoan reference database RefSeq 63 and the invertebrate genetic code. Circularized mitochondrial genome maps (Figure A1) were generated with OrganellarGenome‐DRAW (OGDRAW) online server v 1.3.1 (Greiner et al., 2019). Manual curation of the mitogenomes was undertaken using previously published mitogenomes of bryozoans available on NCBI as references. In cases where incomplete mitogenome contigs were not recovered, Exonerate v2.4.0 (Slater & Birney, 2005) with the affine: local model and maximum intron length set to 40 kb was used to scan the remaining contigs in the assemblies to identify any missing mt genes (13 protein‐coding genes [PCG] and 12S and 16S rRNA genes; transfer RNAs were not scanned). 18S and 28S rRNA genes were annotated using RNAmmer (Lagesen et al., 2007).

### 2.5 Phylogenetic analysis

For phylogenetic inference we used twelve PCGs (cox1, cox2, cox3, cob, nad1, nad2, nad3, nad4, nad4l, nad5, nad6 and atp6), two mt rRNA genes (12S and 16S) and two nuclear rRNA genes (18S, 28S; Table A2). The PCGs were translated into amino acids and aligned with MAFFT v7.310 (Katoh et al., 2002; Katoh & Standley, 2013) with the parameters: auto, localpair, maxiterate 1000. Ambiguously aligned amino acids were removed using BMGE v. 1.12.2 (Criscuolo & Gribaldo, 2010). The rRNA genes were aligned with MAFFT using the same settings as above. Ambiguously aligned nucleotide positions were removed with trimAl v1.4. rev15 (Capella‐Gutiérrez et al., 2009) using the parameters gt 0.6 and some manual adjustments. Finally, the single gene alignments were concatenated into a supermatrix using AMAS (Borowiec, 2016).

Phylogenetic trees were constructed using Bayesian inference (BI) and maximum likelihood (ML) on a mixed partitioned data matrix including 16 partitions (12 PCGs, two mt rRNA genes (12S and 16S) and two nuclear rRNA genes (18S and 28S)). Mt PCGs were processed as amino acids while mt rRNA and nuclear rRNA genes as nucleotides. The best‐fitting evolutionary model for each partition was estimated using ModelTest‐NG v0.1.7 (Darriba et al., 2019) based on the corrected Akaike Information Criterion. The GTR+I+G4 was the best‐fitting model for the rRNA genes and the MtZoa+G4+F was the best‐fitting model for the PCGs. The ML tree was inferred using RAxML‐NG v. 1. 0. 2 (Kozlov et al., 2019) using the best‐fitting model for each partition as determined by ModelTest‐NG. Topological support was assessed with 1000 bootstrapping replicates. The BI analysis was conducted with MrBayes5d 3.2.6 (https://github.com/astanabe/mrbayes5d: last accessed on 26.02.2023), a modified version of MrBayes 3.1.2 incorporating the MtZoa evolutionary model (Ronquist & Huelsenbeck, 2003). Analyses were composed of two independent runs with four Markov Chain Monte Carlo (MCMC) chains, each. Chains were run for five million generations. Tree and parameter sampling were every 100th generation. The GTR+I+G4 model was used to correct for multiple substitutions of the nuclear and mt rRNA gene partitions, and the MtZoa+G4 model was used for mt PCGs gene partitions. Convergence of the MCMC chains was assessed by inspection of the tracefile outputs in Tracer (Nascimento et al., 2017). The convergence was also assed based on the average standard deviation of split frequencies (ASDOSF) and was <0.01 (0.000034)” The first 25% of samples were discarded as burn‐in, and the remaining trees were used to calculate posterior probability values and to build the consensus tree. The final ML and BI trees were visualized and adjusted in Figtree v1.4.4 (http://tree.bio.ed.ac.uk/software/figtree/).

### 2.6 Genetic distance

ML‐corrected substitutions per site were calculated in MEGA 7 using the maximum composite likelihood parameter with a gamma parameter of 1.0 (Kumar et al., 2008; Tamura et al., 2004, 2021).

### 2.7 Alignment

We generated sequences of 27 specimens that belong to 25 morphospecies and successfully assembled and annotated all PCGs, two rRNAs and two nuclear rRNA genes of 20 specimens while the atp8 gene was not recovered in seven samples (Table A2). As a result, the atp8 gene was excluded from our final data matrix, together with 18S and 28S of *Terebripora* sp. The remaining sequences of these 27 samples were combined with published sequences of the ctenostome *Monobryozoon ambulans* (Schwaha et al., 2024), nine cheilostome species (Orr et al., 2021) and the phylactolaemate *Pectinatella magnifica* as outgroup (Fuchs et al., 2009; Gim et al., 2018; Waeschenbach et al., 2009; Table A2).

Our data matrix included 16 genes (12 mt PCGs, two mt rRNA and two nuclear rRNA genes) totaling 9702 characters (2834 amino acids and 6868 nucleotide sites).

## 3 RESULTS

### 3.1 Phylogenetic analysis

#### 3.1.1 Ctenostome phylogeny and placement of *Penetrantia*

**FIGURE 1:**
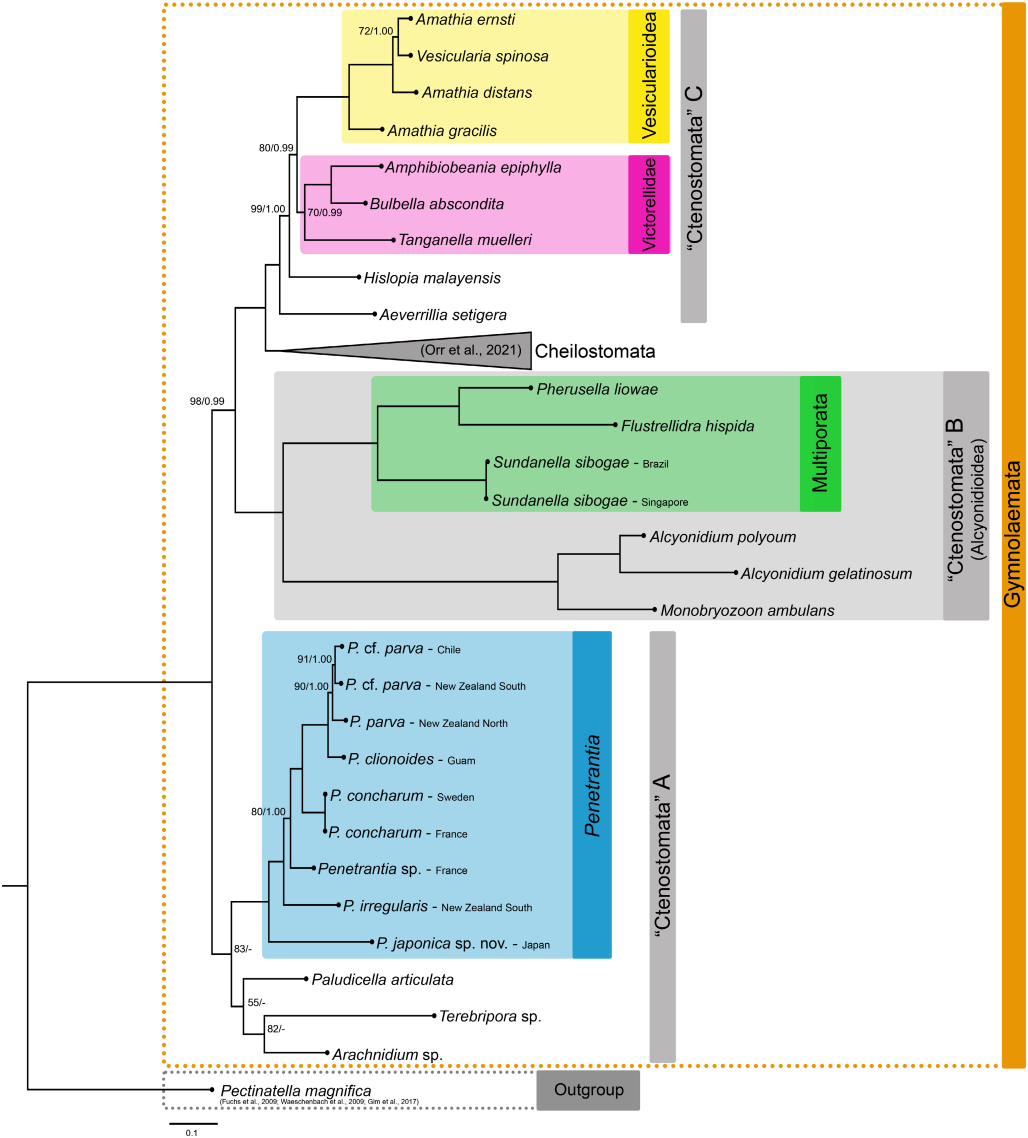
Maximum Likelihood phylogenetic tree based on a data matrix of 16 genes comprising 28 ctenostomes, 9 cheilostomes from Orr et al., 2021 (branch collapsed) and the phylactolaemate bryozoan *Pectinatella magnifica* (from Fuchs et al., 2009; Gim et al., 2018; Waeschenbach et al., 2009) as an outgroup to root the phylogenetic tree (see Table A2). Values on internal nodes correspond to ML bootstrap support (1000 replicates) and posterior probabilities for BI (based on the last 75% of trees) respectively. Values are only shown for nodes that are not fully supported by both phylogeny reconstruction methods. Different colored boxes represent different clades ‐ orange: Gymnolaemata, blue: *Penetrantia*, green: Multiporata, pink: Victorellidae, yellow: Vesicularioidea. Gray: three main clades **A**, **B** and **C**. Clade **B** reflects the superfamily Alcyonidioidea. Cheilostomata has been collapsed to allow better visualization. The scale bar represents 1 substitutional change per 100 character positions.

**FIGURE A2:**
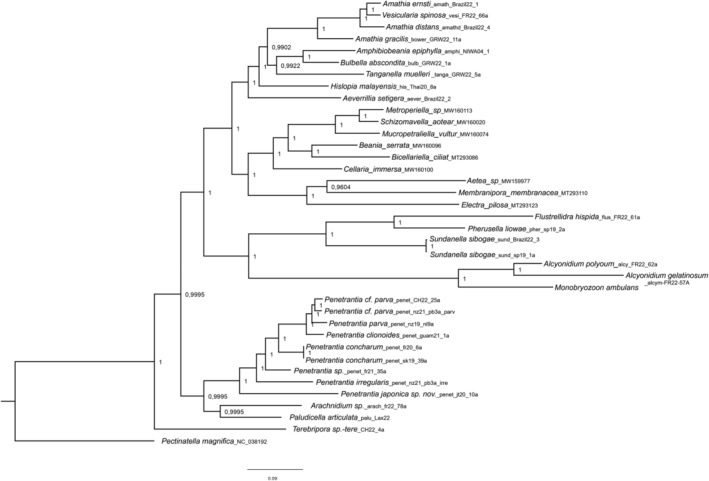
Bayesian Inference phylogenetic tree based on a data matrix of 16 genes comprising 28 ctenostomes, 9 cheilostomes from Orr et al., 2021 (branch collapsed) and the phylactolaemate bryozoan *Pectinatella magnifica* (from Fuchs et al., 2009; Gim et al., 2018; Waeschenbach et al., 2009) as an outgroup to root the phylogenetic tree (see Table A2). Values on internal nodes correspond to BI posterior probabilities.

The ML tree, which is based on the complete data matrix, is shown in Figure 1. Highly consistent tree topologies were observed from both phylogeny reconstruction methods (ML, Figure 1 and BI, Figure A2). The phylogeny is robust, and most nodes are either fully supported (100 bootstrap (BS)/1.00 Posterior Probability (PP)) or highly supported (>90 BS/>0.99 PP); while only four nodes have moderate support (<80 BS). Hereafter, only supports below 100 BS and 1.00 PP will be mentioned as all remaining branches are fully supported.

Gymnolaemata includes the paraphyletic ctenostomes and the monophyletic cheilostomes. Ctenostomes form three main clades: **A**, **B** and **C**. Clade **A** is well supported (83 BS) and includes the monophyletic Penetrantiidae as sister group to a moderately supported clade (55 BS) comprising *Paludicella articulata*, *Arachnidium* sp. and *Terebripora* sp. Within this clade *P. articulata* is the sister to *Arachnidium* sp. and *Terebripora* sp. with the latter forming a well‐supported (83 BS) sister group relationship (Figure 1).

Clade **B** represents the fully supported superfamily Alcyonidioidea and is divided into two clades, one comprising *Alcyonidium* and *Monobryozoon* and the other including *Pherusella*, *Flustrellidra* and *Sundanella*, the latter representing Multiporata. *Monobryozoon ambulans* is the sister taxon to the monophyletic Alcyonidiidae represented here by two species of the genus *Alcyonidium*. Within Multiporata, *Sundanella sibogae* forms the sister taxon to a clade comprising *Pherusella liowae* and *Flustrellidra hispida*. The two representatives of *S. sibogae* from Brazil and Singapore display a genetic divergence of only 0.5% (cox1) and are therefore considered to represent the same species (Figure 1, “Ctenostomata” B).

The fully supported clade **C** is the sister taxon to cheilostomes, thus confirming the paraphyletic status of ctenostomes, and includes all remaining ctenostomes in this study: Vesicularioidea, Victorellidae, as well as the walkerioid *Aeverrillia setigera* and the hislopioid *Hislopia malayensis*. The representative of the Walkerioidea superfamily *A. setigera* represents the sister taxon to all other members of Clade **C**. *Hislopia malayensis* is the sister taxon to victorelloids and vesicularioids with high support (99 BS/1.00 PP). Victorellidae and Vesicularioidea each are monophyletic, although the monophyly of Victorellidae is only supported moderately (70 BS/0.99 PP). Both taxa form a highly supported sister‐group relationship (80 BS/0.99 PP). Within Victorellidae, *Tanganella muelleri* is the sister taxon to *Bulbella abscondita* and *Amphibiobeania epiphylla*. Among Vesicularioidea, *Amathia gracilis* is the sister taxon to the paraphyletic assemblage of *Amathia* with the inclusion of *Vesicularia*. With moderate support (72 BS/1.00 PP), *Amathia ernsti* and *Vesicularia spinosa* cluster together with *Amathia distans* as sister taxon (Figure 1, “Ctenosotmata” C).

#### 3.1.2 Interrelationships of *Penetrantia* and their genetic distances

**FIGURE 2:**
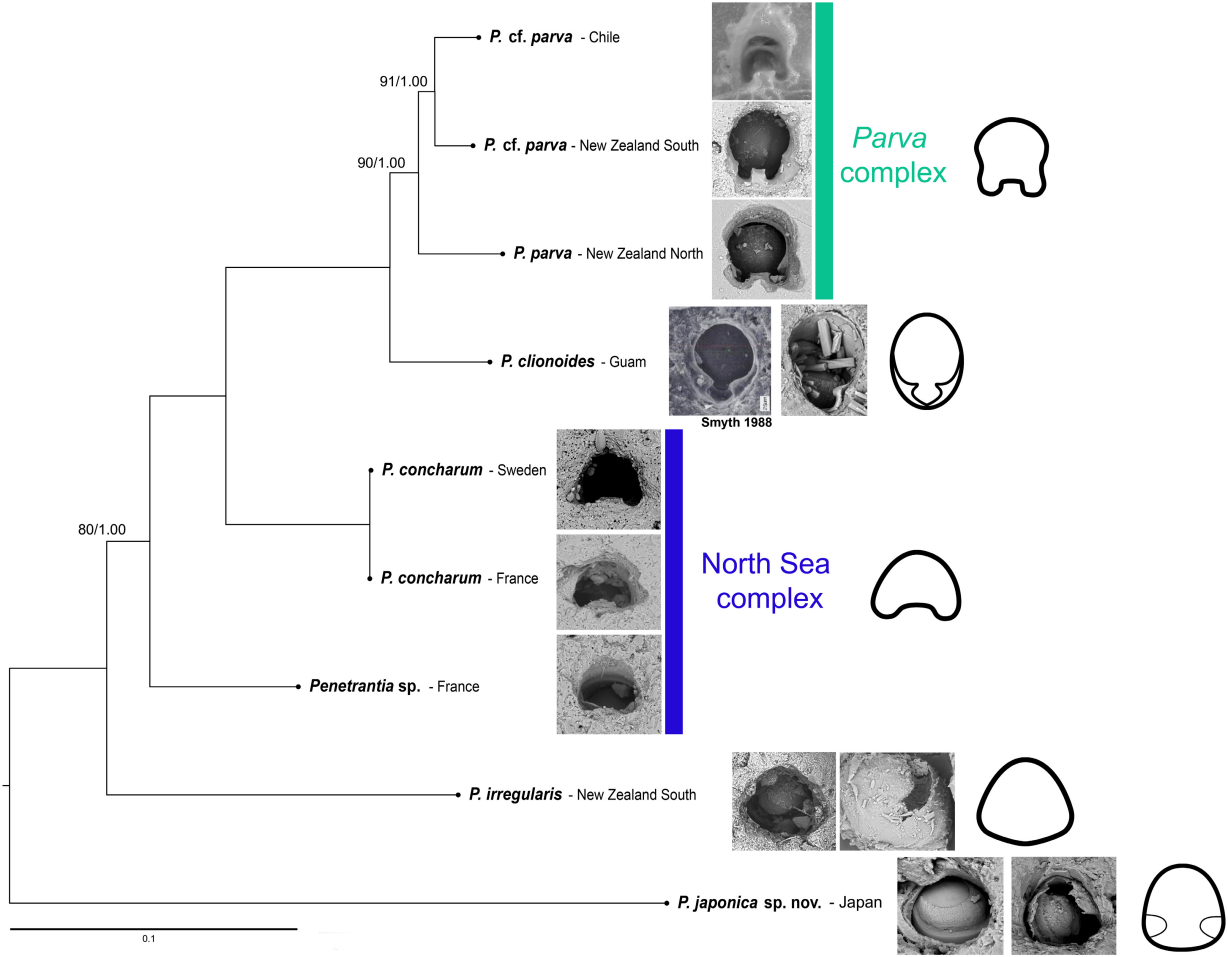
Interrelationships of nine penetrantiids from seven different regions. Subtree of the genus *Penetrantia* derived from the phylogeny shown in Figure 1. Values on internal nodes correspond to ML bootstrap support (1000 replicates) and posterior probabilities for BI (based on the last 75% of trees) respectively. Values are only shown for the nodes that are not fully supported by both phylogeny reconstruction methods. Images show borehole aperture and/or operculum of respective species. The borehole apertures have a diameter of 80–100 μm. The image of the borehole apertures of *P. clionoides* is modified from Smyth, 1988. Drawings are generalized outlines of the borehole apertures. Two cryptic species complexes ‐ green: North Sea‐complex, blue: *parva*‐complex. The scale bar represents 1 substitutional change per 100 character positions.

*Penetrantia japonica* sp. nov. is the sister taxon to all other *Penetrantia* species in our study. The next branch is formed of *Penetrantia irregularis* from New Zealand and is well separated from the other New Zealand penetrantiids of the *parva* clade. With moderate support (80 BS/1.00 PP), *Penetrantia* sp. from France (Roscoff) is the sister taxon to a clade composed of *Penetrantia concharum* from Sweden and France (Roscoff), *Penetrantia clionoides* from Guam and representatives of the *parva* complex from Chile and New Zealand. *Penetrantia clionoides* is the sister taxon to the *Penetrantia parva* complex (Figure 2). Both species from France possess *concharum*‐like borehole apertures, which are typically kidney‐shaped; however, the cox1 genetic divergence is 21.9%, confirming them to be different species. Contrary, *P. concharum* specimens from Sweden and France exhibit a genetic distance of 0.3% (Figure 2; Table A3).

*Penetrantia* sp. from France (Roscoff) is also confirmed in Germany (Helgoland) with a genetic divergence of 2.3% based on the barcoding region of the cox1 gene (Table A4).

The *parva* complex forms a monophyletic clade with high support (90 BS/1.00 PP) and all three representatives exhibit the species‐specific aperture outline with prominent apertural notches (Figure 2). *Penetrantia parva* from the northern Island of New Zealand is the sister taxon to a clade represented by *P*. cf. *parva* from the southern Island of New Zealand and *P*. cf. *parva* from Chile with moderate support (91 BS/1.00 PP; Figure 2). The cox1 genetic distances between representatives of this complex are: northern and southern Islands of New Zealand – 12.1%; *P. parva* from northern New Zealand and *P*. cf. *parva* from Chile – 12.9%; southern New Zealand and Chile – 9.8% (Table A3).

### 3.2 Systematic account/species description of *Penetrantia japonica* sp. nov.

Phylum Bryozoa Ehrenberg, 1831.

Class Myolaemata Schwaha et al., 2020.

Subclass Gymnolaemata Allman, 1856.

Order *Ctenostomata* Busk, 1852 asterisk indicating the paraphyletic status.

Family Penetrantiidae Silén, 1946.

Genus Penetrantia Silén, 1946.

*Penetrantia japonica* sp. nov.

*Penetrantia* sp. Decker et al., 2023, figures 3, 5, 8, 9, 13, 14, 17, 21 and 22.

**FIGURE 3:**
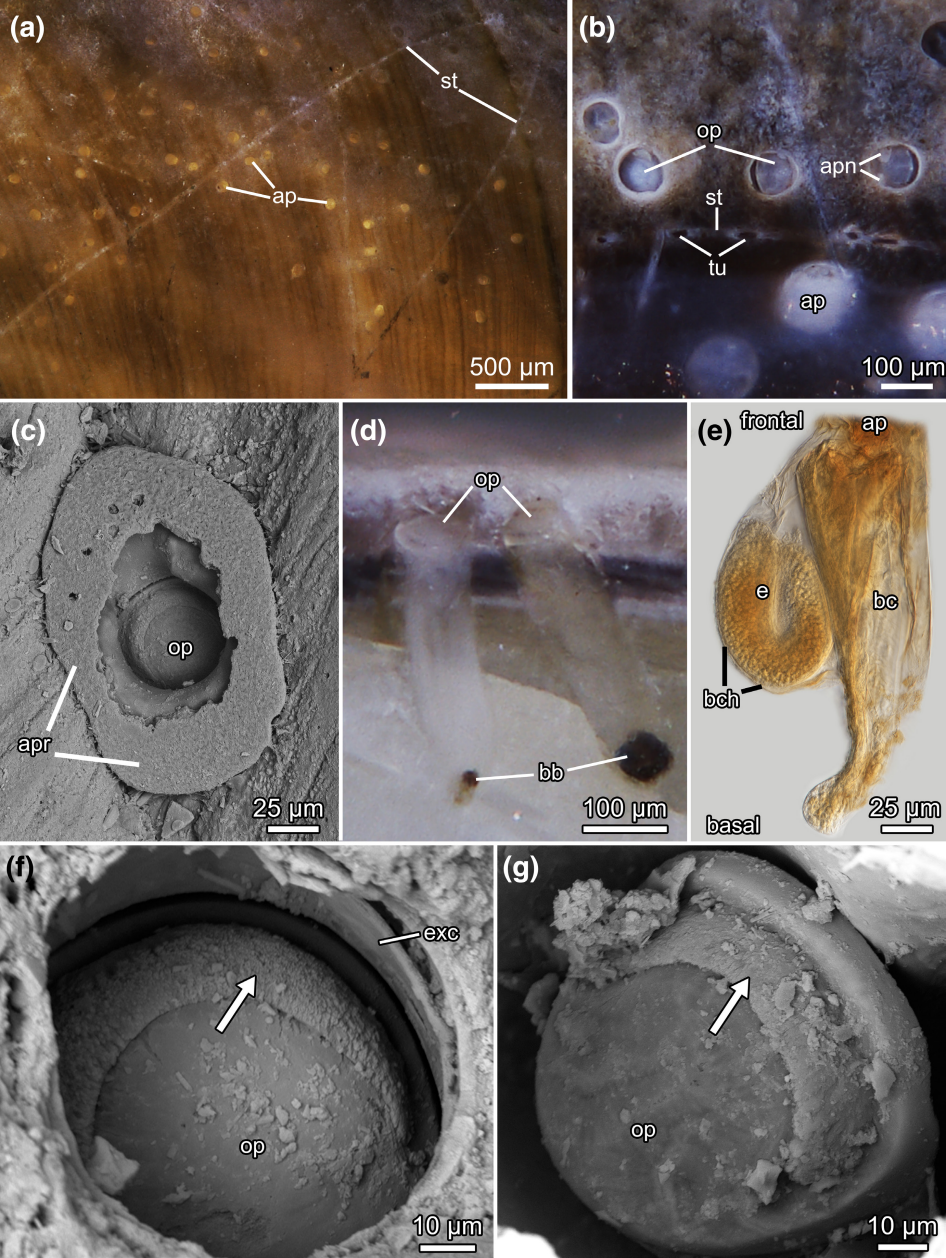
*Penetrantia japonica* sp. nov. from Japan. Stereomicroscopic images of the holotype (NSMT‐Te1270) in the shell of the gastropod *Tegula rugata* (A. Gould, 1861) (a) and (d). Borehole apertures with apertural notches in the gastropod *Japeuthria ferrea* (Reeve, 1847) (b). Scanning electron microscopic images of a borehole aperture with apertural rim (c). Stereomicroscopic image of a broken shell piece with two autozooids in lateral perspective, showing their penetration depth and the almost vertical orientation within the substrate (d). Microscopic image of a wholemount showing a gonozooid with its unique basal extension (e). Scanning electron microscopic images of the operculum in (f) and (g) with its peculiar crescent‐shaped and rough area on the frontal side (arrows). ap – aperture, apn – apertural notches, apr – apertural rim, bb ‐ brown body, bc – body cavity, bch – brood chamber, e – embryo, exc ‐ exterior cuticle, op – operculum, st – stolon, tu – tubulet.

**Type material**: *Holotype*: NSMT‐Te1270, National Museum of Nature and Science, Tokyo, Japan. Collected at Tenjin‐Jima Island, Sagami Bay, Japan (35°13.336′ N 139°36.152′ E), intertidal, 29th October2020, by Masato Hirose. In shell of hermited *Tegula rugata* (A. Gould, 1861) (Figure 3a). All paratypes were collected at the same location as the holotype. *Paratype1*: NSMT‐Te1271, collected 29th October 2020, hermited *T. rugata. Paratype2*: NSMT‐Te1272, collected 4th November 2020, hermited *Reishia clavigera* (Küster, 1860)*. Paratype3*: NSMT‐Te1273, collected 19th October 2020, live *Japeuthria ferrea* (Reeve, 1847).

**Diagnosis**: Penetrantiid bryozoan with calcified operculum, frontal side of operculum with rough crescent area, large tubulet intervals (180–200 μm) and gonozooid with lean basal tip bent slightly in frontal direction.

**Description**: Boring traces commonly found in live or hermited intertidal gastropod shells (*T. rugata*, *J. ferrea*, *R. clavigera*). Young colonies often close to aperture of gastropod shell, larger colonies all over shell, commonly close to apex. Typical feather‐shaped colony with leading principal stolon and secondary stolons branching of orthogonally. Older colonies strongly ramified with different stolons intercrossing, generating mesh‐like pattern (Figure. 3a). True kenozooidal stolons separated by distinct septa/pore plates. Zooids pedunculate, placed along both lateral sides of stolons (Figure. 3a,b). Borehole apertures 80–100 μm in width, circular to keyhole‐shaped, sometimes with small apertural notches on oral side, rarely with calcareous apertural rim (Figure. 3b,c). Tubulet pores often visible, small holes along stolons in intervals of 180–200 μm, 8–12 μm in width (Figure 3b). **Autozooids** tubular with slightly pointed basal tip, vertically in substrate, 380–430 μm in length, 120 μm in width (Figure 3d). Always 12 tentacles. Prominent exterior cuticle, extending far frontally of operculum (Figure 3f). Operculum about 100 μm in width, dome‐shaped in cross section, rough crescent area on frontal‐oral side, partially composed of calcium carbonate (Figure 3f, g). Multiple brown bodies common (Figure 3d). **Gonozooid** same length as autozooid, brood chamber on anal side about 230 μm long, pear‐shaped in longitudinal section. Gonozooidal tube longer than brood chamber with slandered basal tip, bending in anal direction. Operculum same as for autozooid. Polypide reduced, no tentacles (Figure 3e).

**Etymology**. Japonica is the adjective in the feminine case, referring to Japan, which represents the type locality of this possibly endemic species.

**Distribution**. Yoshihama Bay, Iwate Prefecture, Japan (39°6.984′ N 141°52.355′ E) and along the coast of Sagami Bay, Japan (35°13.336′ N 139°36.152′ E).

**Remarks**. The operculum morphology is very similar between *P. japonica* sp. nov., *P. clionoides* Smyth, 1988 and *Penetrantia bellardiellae* Schwaha, 2019. The opercula of these three species are composed of calcium carbonate, with a rough crescent‐shaped area on the frontal side and thereby tells them apart from all other penetrantiids. In contrast to *P. clionoides* and *P. bellardiellae*, *P. japonica* sp. nov. has the largest tubulet intervals and unique gonozooids with a lean basal tip which bends slightly in frontal direction.

**Zoobank.** urn:lsid:zoobank.org:act:702B1421‐5 CE9‐4D26‐8 EB3‐8A2BA4AA83F8.

## 4 DISCUSSION

### 4.1 Interrelationships of “Ctenostomata” and their paraphyly

Gymnolaemata is a widely accepted monophyletic class of bryozoans which is the sister taxon to Stenolaemata (Schwaha, 2020a, 2020d; Todd, 2000; Waeschenbach et al., 2012). Within Gymnolaemata, ctenostomes form a paraphyletic assemblage that includes the monophyletic cheilostomes (Fuchs et al., 2009; Schwaha, 2020a; Waeschenbach et al., 2012). However, the taxon sampling of ctenostomes for phylogenetic analyses was rather poor until now. Our study represents the broadest taxon sampling to date and resulted in three main clades of ctenostomes. The first main clade (**A**) includes representatives of four different families of ctenostome bryozoans (Paludicellidae, Arachnidiidae, Terebriporidae and Penetrantiidae). Although a close relationship between the superfamilies Arachnidioidea and Paludicelloidea was previously proposed (Jebram, 1973; Schwaha, 2020c; Todd, 2000; Waeschenbach et al., 2012), all three groups (including penetrantiids) possess distinct morphological traits that make their close relationship unexpected. Most arachnidioids are characterized by cystid appendages that can be anastomosing and create “pseudostolonal” connections between zooids (Jebram, 1973; Schwaha & De Blauwe, 2020), though some species in this group lack such appendages (see Jebram, 1986). *Paludicella articulata* lacks such cystid appendages, has a unique cruciform branching pattern and is restricted to freshwater habitats. These major differences led to the placement of *Paludicella* into the separate superfamily Paludicelloidea (Jebram, 1973; Schwaha, 2020c; Todd, 2000). The family Terebriporidae is one of four Recent endolithic ctenostome families and was considered a vesicularioid ctenostome with true stolonate colonies, and the presence of a gizzard was previously reported (Schwaha, 2020c; Soule & Soule, 1969). However, this information about the soft body morphology in terebriporids remains doubtful (Pohowsky, 1978). Subsequent histological analyses are necessary to clarify whether terebriporids have kenozooidal stolons or arachnidioid‐like cystid appendages and if they feature a gizzard or not (Pohowsky, 1978). Since this clade shows only a moderate support and a different topology within the BI analysis, a denser taxon sampling that includes more members of the Arachnidioidea, e.g., Immergentiidae and Nolellidae, is required to better resolve the unexpected sister‐group relationship of *Paludicella* with *Arachnidium* and *Terebripora*. The fourth family within clade **A**, Penetrantiidae, possess many distinct characters (e.g., operculum and kenozooidal stolons) that are not present in Paludicellidae or Arachnidioidea and will discussed in more detail later (see below) (Jebram, 1973; Schwaha, 2020c).

The second main clade (**B**) represents the superfamily Alcyonidioidea, a taxon characterized by tightly arranged zooids that are always in close contact with the body wall of neighboring zooids and never by stolon‐like connections (Schwaha, 2020c). Unlike other studies (Jebram, 1986; Todd, 2000; Waeschenbach et al., 2012), our phylogenetic analysis does not support alcyonidioids as sister taxon to all remaining ctenostomes but of clade **A** instead (Arachnidiids, Paludicellids and Penetrantiids). Consequently, our study indicates that the serial arrangement of zooids as found in paludicellids, arachnidioids and penetrantiids could represent the ancestral colony structure of ctenostomes rather than simple encrusting sheet‐like colonies of alcyonidioideans as previously suggested (Jebram, 1973; Schwaha, 2020c). Certainly, a larger taxon sampling within both clades might alter the phylogeny, since arachnidioids are only represented by one species in this study.

The only superfamily not included in the current study is Benedeniporoidea and was previously considered the sister taxon to all remaining ctenostomes. This led to the establishment of the “Protoctenostomata” – “Euctenostomata” concept, with Benedeniporoidea as early protoctenostome and all remaining Recent ctenostomes belonging to euctenostomes (Jebram, 1973; Todd, 2000). However, this phylogenetic hypothesis would imply that a ctenostome‐like ancestor possessed serially erect colonies, which is a rare state among Recent ctenostomes. Additionally, species of this superfamily were only rarely found and detailed information on their morphology as well as sequence data is missing (Schwaha, 2020c).

Multiporata, a recently erected taxon of alcyonidioid bryozoans that is characterized by multiporous pore plates, is monophyletic and nests within Alcyonidioidea. These distinct pore‐plates are usually known from cheilostomes and not found in other ctenostome bryozoans (Schwaha, Winston, et al., 2022). The multiporate genera *Flustrellidra* and *Pherusella* are sister taxa in our analysis and share some specific characters, e.g., a rectangular to bilateral shaped orifice and pseudocyphonautes larvae. The latter is only present in these two families and resembles an apomorphy of this group (Decker et al., 2020, 2021; Reed, 1991). This close relationship was also shown by a recent phylogenomic study (Saadi et al., 2022). Our study supports the affiliation of sundanellids to Multiporata and not to Victorelloidea. This affiliation is supported by several morphological characters e.g., multiporous pore plates, large bilateral lophophores with high tentacle numbers (more than 30) and a vestibular collar (Schwaha, Winston, et al., 2022). A close relationship of *S. sibogae* to the multiporate *F. hispida* was recently indicated by a phylogenetic analysis based on the nuclear marker 18S gene (Schwaha, Waeschenbach, et al., 2022). Remarkably, *S. sibogae* is confirmed in Singapore as well as in Brazil by our study and thereby underlines its vast distribution. *S. sibogae* was reported from Indonesia, Singapore, the eastern and western coast of Africa and the Western Atlantic before (Marcus, 1937, 1941; Harmer, 1915; Schwaha, Winston, et al., 2022;Vieira et al., 2014). The only multiporate genus not included in our study is *Elzerina*, which is currently placed in the family Flustrellidridae. However, the presence of pseudocyphonautes larvae in *Elzerina* (like in *F. hispida*) is not confirmed but internal brooding of lecithotrophic larvae seems possible. Since an intertentacular organ is only present in *Elzerina* and neither in *Flustrellidra* nor in *Pherusella*, the latter two genera may share a closer relationship (Schwaha, 2021). Consequently, future studies should include sequence data of the genus *Elzerina* to confirm this idea.

Our study also suggests a sister‐group relationship between the genus *Alcyonidium* and *M. ambulans*. The latter is a solitary bryozoan species living in sandy marine sediments as part of the meiofauna and was just recently rediscovered (Remane, 1936; Schwaha et al., 2024). Monobryozoidae was traditionally placed either among arachnidioids, primarily due to the presence of non‐kenozooidal cystid appendages (Jebram, 1986), or as incertae sedis (D'Hondt, 1983). More recent investigations suggest an affinity of monobryozoids with alcyonidioids (Schwaha, 2020c; Schwaha et al., 2024), which is also confirmed in our study. This affinity is reflected by alcyonidioid‐like characters such as a circular orifice, the presence of a prominent orifical sphincter and a vestibular anus (Schwaha, 2020c; Schwaha et al., 2024).

The third main clade (**C**) includes species of four different ctenostome superfamilies, two of them are characterized by kenozooidal stolons as found in penetrantiids. The origin of cheilostomes within ctenostomes is the most accepted scenario and supported by morphological and molecular data and thereby renders ctenostomes paraphyletic (Orr et al., 2022; Waeschenbach et al., 2012). However, it was still unclear which of the Recent ctenostome clades is the closest relative to cheilostomes. Former investigations suggested a close relationship and potential ancestry of cheilostomes with *Arachnidium*‐like ctenostomes (Banta, 1975; Taylor, 1986, 1990). More recent studies favor a sister‐group relationship of cheilostomes to the ctenostome superfamilies Hislopioidea and Vesicularioidea (Waeschenbach et al., 2012). Our study suggests a similar sister‐group relationship of cheilostomes; however, additionally includes representatives of Walkerioidea and Victorellidae, which were not included in Waeschenbach et al. (2012). Thus, it seems reasonable that cheilostomes and the superfamilies in clade **C** (Walkerioidea, Victorellidae, Hislopioidea, Vesicularioidea) share a most recent common ancestor. Future studies should continue to tackle this question by increasing taxon sampling especially including more representatives of walkerioid and hislopioid bryozoans.

Regarding the sister‐group relationship of Victorelloidea and Vesicularioidea, it is evident that they share a well‐developed funicular system and a cardiac constrictor often with a gizzard (Schwaha, 2020c). However, while vesicularioid bryozoans are characterized by zooids that always are connected by true kenozooidal stolons, victorelloids lack stolons and are restricted to brackish and freshwater habitats (excluding sundanellids) (Schwaha, 2020c). Based on morphological characters, the superfamily Victorelloidea was previously considered to be polyphyletic, which is supported in our analysis by the placement of *Sundanella* within Multiporata (see also Schwaha, Waeschenbach, et al., 2022; Schwaha, Winston, et al., 2022). Consequently, a morphological revision of Victorelloidea, with the exclusion of *Sundanella*, may reveal additional shared characters. In our analysis, *A. epiphylla* clusters together with the remaining two victorelloid species, with *B. abscondita* as sister taxon and *T. muelleri* being the sister taxon to both aforementioned. Formerly, *A. epiphylla* was regarded as cheilostome bryozoan due to the presence of an opercular‐like structure (Metcalfe et al., 2007). Recent morphological investigations proved typical ctenostome features (denticulate gizzard, low tentacle numbers (eight), a large number of interzooidal pore plate cells and the lack of duplicature bands) and indicate a potential affinity with vesicularioids and victorelloids (Schwaha, Waeschenbach, et al., 2022). An operculum was not confirmed in *A. epiphylla* and therefore assumed to be absent (Schwaha, Waeschenbach, et al., 2022). Furthermore, a phylogenetic analysis based on the 18S gene revealed its ctenostome affinity (Schwaha, Waeschenbach, et al., 2022), which is also confirmed in our analysis. Since this species was only reported from mangroves it may be adapted to brackish environments with changeable salinities, which again might cohere with a victorelloid affiliation of *A. epiphylla* (Metcalfe et al., 2007; Schwaha, 2020c; Schwaha, Waeschenbach, et al., 2022).

Within Vesicularioidea, *A. gracilis* is the sister taxon to all remaining *Amathia* species as well as to *V. spinosa*. *Amathia gracilis* was previously placed in the genus *Bowerbankia* and just recently re‐assigned to *Amathia* (Waeschenbach et al., 2015).

### 4.2 A ctenostome affiliation of Penetrantiidae and their closest relatives

Our analysis confirms a ctenostome affiliation of Penetrantiidae as suggested by several morphological studies previously (Decker et al., 2023; Pohowsky, 1978; Schwaha, 2020c; Silén, 1946, 1947), and contradicts other studies that favored a cheilostome affinity (Smyth, 1988; Soule & Soule, 1969). Especially, the presence of cheilostome‐like features such as the operculum and the brood chamber started a long‐lasting discussion on the placement of penetrantiids. However, these structures appear to have evolved convergently in Penetrantiidae and Cheilostomata, since there are major morphological differences, particularly in the underlying musculature (see Decker et al., 2023).

Additionally, the absence of opercula and brood chambers in the closely related taxa (*Paludicella*, *Terebripora* and *Arachnidium*) points to apomorphic characters of Penetrantiidae. *Paludicella pentagonalis* differs in its colony pattern from *P. articulata*, in contrast to the *P. articulata*, *P. pentagonalis* has a linear series of zooids with no lateral branches (Annandale, 1916). *Paludicella pentagonalis* is also reported to sometimes possess “stolon‐like” connections between zooids (see Rogick & Brown, 1942) that might support a potential relationship of *P. pentagonalis* with arachnidioids or penetrantiids. Therefore, it would be essential to investigate whether these stolon‐like tubes feature pore plates because no information is currently available about the kenozooidal status of these tubes limiting their phylogenetic value.

The presence of true polymorphic stolons in penetrantiids traditionally favored a close relationship with the other stolon‐bearing groups vesicularioids or walkerioids (Decker et al., 2023; Schwaha, 2020c). The potential presence of a gizzard also supported a vesicularioid affinity (Pohowsky, 1978; Schwaha, 2020c; Silén, 1946, 1947). However, the other two stolonate groups are not considered closely related to penetrantiids and also do not form a monophyletic group. How terebriporids fit into this clade remains questionable, but they maybe lack true stolons after all, which could explain the unexpected close relationship with *Arachnidium* (see above). Consequently, our study suggests that kenozooidal stolons have evolved at least three times independently within ctenostomes (vesicularioids, walkerioids and penetrantiids). The polyphyly of the artificial construct of “Stolonifera” was already suggested (Jebram, 1973; Schwaha, 2020c) and is also supported by recent molecular studies (Waeschenbach et al., 2012, 2015). This hypothesis is also based on several morphological and ontogenetical differences in the stolons of these two taxa (see Jebram, 1973; Schwaha, 2020c). Stolon‐like structures are also present in other groups of bryozoans and essential in the formation of characteristic colony forms and in the interaction and competition between other sessile organisms (Pohowsky, 1978; Schack et al., 2019). Stolons might also support faster propagation and expansion of colonies, ensuring good colony interconnectivity and the distribution of metabolites throughout a colony (Jebram, 1973; Pohowsky, 1978). Growth experiments on *Penetrantia* showed that their stolons grow relatively fast, which probably helps them invade new substrates faster and outcompete other endolithic organisms (Decker et al., submitted). In endolithic bryozoans, the main advantage of stolonate colonies with spaced zooids probably lies in decelerated substrate deterioration, thereby ensuring substrate stability (Decker et al., 2023; Pohowsky, 1978). This might explain why all endolithic bryozoans have relatively long kenozooidal stolons or long cystid appendages between their zooids.

Furthermore, the presence of a true gizzard in penetrantiids was questioned, as the gizzard‐like structure is indistinct, does not feature denticles and thereby resembles a proventriculus (Decker et al., 2023). Overall, a closer relationship of penetrantiids and vesicularioids is unlikely.

### 4.3 Interrelationship of Penetrantiidae

The sequences of nine different penetrantiid specimens correspond to eight genetically diverged species in our analysis. However, there are two cryptic species complexes present, which can be hardly differentiated based on morphological characters. Cryptic speciation is a common phenomenon known from many different groups of bryozoans becoming more evident with the increase of molecular investigations (Chimenz Gusso et al., 2004; Fehlauer‐Ale et al., 2014; Thorpe & Ryland, 1979; Waeschenbach et al., 2015). Particularly, the soft bodied ctenostomes, without any distinct skeletal characters, are prone to this taxonomic issue (Thorpe et al., 1978; Waeschenbach et al., 2015).

We unraveled two cryptic species complexes within the genus *Penetrantia*: (1) a species complex in the North Sea and the Northern Atlantic and (2) *parva* complex in the Southern Pacific. The species assembly in the Northern Atlantic is intriguing since at least two similar species do co‐occur in the same region (Roscoff, France), *P. concharum* and *Penetrantia* sp. *Penetrantia concharum* from Roscoff is genetically identical to *P. concharum* from Sweden while *Penetrantia* sp. from Roscoff is genetically very different from *P. concharum* and most likely represents an undescribed species. Although *P. concharum* and *Penetrantia* sp. do not form a monophyletic clade in our analysis, their morphology is very similar and they form almost identical borehole apertures and colonies. A recent study found minor soft body differences between *Penetrantia* sp. and *P. concharum*. For instance, *Penetrantia* sp. features a collar, has a thinner operculum and on average smaller autozooids than *P. concharum* from Sweden (see Decker et al., 2023). However, since these morphologically investigated specimens were not sequenced it is not possible to assign these characters to one species with certainty. Furthermore, there are reports of *Penetrantia* along the Iberian coast that were not assigned to one of the known European penetrantiid species (*P. concharum* or *Penetrantia brevis*) and might represent the undescribed species in Roscoff (Decker et al., 2023; Reverter‐Gil et al., 1995, 2016; Reverter‐Gil & Souto, 2014). The picture becomes even more complex as the undescribed *Penetrantia* sp. from Roscoff (France) is also confirmed in Helgoland (Germany), which is geographically much closer to Sweden than France, and suggests an overlapping distribution of both species in the North Sea and the Northern Atlantic. Consequently, a much more detailed analysis at population level is required to delineate *Penetrantia* species occurring in the Northern Atlantic that should also include specimens from Norway, United Kingdom, Belgium, Spain and Portugal.

The second cryptic species complex is the *parva* complex distributed throughout the Southern Pacific and represented by three specimens in our study (northern and southern Islands of New Zealand and Chile). *Penetrantia parva* was also reported from New Caledonia and Hawaii and has one of the largest distributions of penetrantiids (see table 2 in Decker et al., 2023). This species complex is morphologically characterized by unique borehole apertures with prominent apertural notches, heavy cuticularized opercula and gonozooids where the brood chamber is half as long as the gonozooid itself (Decker et al., 2023; Silén, 1946, 1947). Interestingly, *P*. cf. *parva* from southern New Zealand is more closely related to the Chilean one than *P. parva* from northern New Zealand. As zooid dimensions are very similar, the only considerable difference is the presence of a shallow pit in the frontal side of the operculum in some specimens of *P. parva* from northern New Zealand. Since this pit was never observed in specimens of the remaining two representatives of the *parva* complex, it might indicate a more distant relationship between *P. parva* from northern New Zealand to both *P*. cf. *parva* from southern New Zealand and Chile (Decker et al., 2023). However, the genetic distances between specimens from all three localities are sufficient (>10%) to consider each of them as a separate species, when applying a cox1 genetic distance of more than 3% as the threshold for species delimitation (see Baptista et al., 2022). The threshold of genetic distance for species delimitation is, however, still debated and depends on the marker gene and the group of animals investigated, but a threshold of about 3% is considered to have the lowest error rate with an optimum of 2.6% for cowrie gastropods (Meyer & Paulay, 2005).

Similar cryptic speciation was observed in the cheilostome *Bugula neritina*, which was considered to have a cosmopolitan distribution, yet only one of the three cryptic species in this complex is distributed globally (Fehlauer‐Ale et al., 2014). On an even smaller geographical scale, cryptic bryozoan species were discovered in a recent study focusing on *Reteporella* species from the Azores and Mediterranean Sea (Baptista et al., 2022). Accordingly, cryptic speciation in bryozoans seems to be unexplored with many cryptic species complexes awaiting their discovery. Considering that most bryozoans have short‐living lecithotrophic larvae, including penetrantiids, the gene flow between populations might be rather restricted, and consequently, speciation may occur on smaller geographical scales (Decker et al., 2023; Gruhl, 2020; Reed, 1991; Todd et al., 1998). However, level of gene flow and genetic structure between populations are not solely explained by pelagic larvae duration (PLD), since there are many examples of species that have a restricted distribution despite having a long PLD and vice versa (Todd et al., 1998).

Similar to the cryptic species complex in the Northern Atlantic, future work should include more specimens from different locations and combine molecular results with thorough morphological investigations. Additionally, it might be important to apply different genetic markers and a larger dataset to better resolve cryptic speciation in *Penetrantia* and to better understand intra‐ and interspecific genetic diversity (see Baptista et al., 2022; Fehlauer‐Ale et al., 2014). Despite large genetic distances, potential new cryptic penetrantiid species should be validated with mating trials to confirm whether they are truly different biological species or not (see Gomez et al., 2007). Nevertheless, there are three penetrantiid species in our study (*P. clionoides*, *P. irregularis* and *P. japonica* sp. nov.) that do not form species complexes and are well separated from other penetrantiids on molecular and morphological basis.

*Penetrantia clionoides* from Guam is the sister taxon of the *P. parva* clade and differs in the morphology of its operculum and gonozooid from the latter. The operculum of *P. clionoides* has a rough crescent‐shaped area on its frontal side and is partially composed of calcium carbonate, which is otherwise only known from *P. japonica* sp. nov. from Japan and *P. bellardiellae* from Papua New Guinea (Decker et al., 2023; Schwaha et al., 2019; Smyth, 1988). Although the latter three species (*P. clionoides, P. bellardiellae* and *P. japonica* sp. nov.) exhibit similar opercula, they clearly differ in terms of gonozooid shape and/or interval length between tubulets (Decker et al., 2023). The geographically closest species to Japan is *Penetrantia taeanata* from South Korea (Seo et al., 2018). This species is much smaller than *P. japonica* sp. nov. and with an average autozooid length of 160 μm by far the smallest penetrantiid (see table 3 in Decker et al., 2023). Consequently, we propose *P. japonica* sp. nov. as a new species here due to distinct morphological differences and discrete phylogenetic placement.

### 4.4 Terebriporidae and the convergent evolution of the boring life style

In our analysis, the family Terebriporidae is represented by a sole species from Chile and is placed among arachnidioid ctenostomes. The original type specimen of the family and genus, *Terebripora ramosa*, was also collected in Chile (d'Orbigny, 1847; Pohowsky, 1978). Characteristic tubulets arising from the zooids were reported for *T. ramosa* along with very symmetrical feeder‐shaped colonies, such that our specimens closely resemble *T. ramosa* (Pohowsky, 1978). However, as there is no information regarding soft body morphology of the latter species, we cannot assign these specimens to *T. ramosa* with certainty. In fact, there is a lot of confusion in the literature about the correct affiliation of many terebriporid species, particularly of fossils. The enantiomorphic apertures of boring traces of *Immergentia* and *Terebripora* can appear very alike and probably led to the wrong assignment of species (Pohowsky, 1978), e.g., *Spathipora comma* (Soule, 1950a) was previously assigned to *Terebripora* and there is still confusion whether *Immergentia philippinensis* Soule, 1950b is a terebriporid or immergentiid species (Bobin & Prenant, 1954; Pohowsky, 1978; Soule, 1950a, 1950b). Accordingly, it is not easy to assign boring traces to a family without information on stolon and gut morphology. In general, an affiliation of boring traces to a family, genus or even a species should be treated carefully as these traces resemble the boring activity of an animal and not true morphological characters. Therefore, such assignments should be considered separate ichnotaxa instead of a true biological species (see Bertling et al., 2006; Decker et al., 2023; Rosso, 2008; Wisshak et al., 2019). The problem becomes even more apparent as the family Terebriporidae was erected based on boring traces and colony patterns alone without any soft body information, rendering the entire family an ichnotaxon (Bertling et al., 2006; d'Orbigny, 1847; Wisshak et al., 2019). Accordingly, a histological reinvestigation of the type material would be necessary to provide soft body information and confirm the taxonomic integrity of the family Terebriporidae.

How many times an endolithic life style has evolved independently remains unanswered. Such an adaptation most likely occurred convergently within the lineage leading to the family Penetrantiidae and within the Arachnidioidea, which includes the boring family Immergentiidae (Pohowsky, 1978; Schwaha, 2020c; Silén, 1947). The fourth Recent endolithic bryozoan family, Spathiporidae, is commonly assigned to vesicularioids and thereby not closely related to the other endolithic taxa, which indicates that the endolithic life style has evolved independently in this group. Spathiporidae and Terebriporidae were considered closely related among the endolithic taxa, since they share unique tubulets arising from autozooids, which the other two families Penetrantiidae and Immergentiidae are lacking. The most distinct difference between spathiporids and terebriporids is the connection of the zooids to their stolonal network. While spathiporids have pedunculate zooids, terebriporids have their zooids placed along the stolons and lack a peduncle (Pohowsky, 1978; Schwaha, 2020c; Soule & Soule, 1975).

Considering that spathiporids are vesicularioids, an endolithic life style should have evolved at least two times independently in ctenostomes and probably an additional time within arachnidioids. With the fossil record dating back to the Ordovician, an early radiation within different ctenostome lineages seems plausible (Pohowsky, 1978).

There seems to be a tendency within ctenostomes towards a boring or burrowing lifestyle as it has also evolved for other substrates, e.g., in wood and parchment‐like polychaete tubes (*B. abscondita* and *Hypophorella expansa* Ehlers, 1876) or inside cheilostome skeletons (*Harmeriella terebrans* Borg, 1940) (Borg, 1940; Pohowsky, 1978). Since *H. expansa* use a specialized gnawing apparatus with teeth to mechanically bore into uncalcified tubes of polychaetes like *Chaetopterus*, its burrowing lifestyle probably evolved independently as well (Borg, 1940; Pröts et al., 2019; Schwaha, 2020c). Colonies of *B. abscondita* live inside degraded wood, which is yet another different substrate, and its burrowing lifestyle most likely evolved independently too, reflected in the separated placement and affiliation of *B. abscondita* to victorelloids (Braem, 1951; Schwaha, 2020c). Overall, such lifestyles have evolved about five times independently within ctenostomes and probably even more often when taking all the different boring ctenostome taxa into account that are only known from the fossil records (Pohowsky, 1978). Such a lifestyle has also evolved multiple times convergently within other groups of invertebrates, such as mollusks and polychaetes. An endolithic lifestyle has evolved at least eight times independently within bivalves, manifested in a wide variety of shell morphologies, including chemical and mechanical borers (Collins et al., 2023). A similar pattern is observed in polychaetes with many boring representatives belonging to different families, which is also reflected in a wide variety of boring methods and constructions of their burrows (Çinar & Dagli, 2021). In sponges, endolithic forms are distributed among at least three different families, indicating a similar convergent radiation of boring species (Van Soest et al., 2012). The overall benefit of such a lifestyle is probably better protection against environmental stressors like waves and currents, but particularly to reduce predation pressure, which might constitute the main driver of the convergent evolution of an endolithic lifestyle in so many different taxa (Collins et al., 2023; Pohowsky, 1978). This holds particularly true for ctenostome bryozoans, which, in contrast to cyclostomes and cheilostomes, lack a calcified body wall (Schwaha, 2020b), thus providing additional protection to the delicate zooids when immersed into substrates (Pohowsky, 1978). However, a potential cost of such a lifestyle is the dependency on calcareous substrates, which limits the spatial and geographical distribution of such species (Collins et al., 2023). Many calcareous substrates that could potentially provide habitats for boring bryozoans, such as whale bones, have not been searched for boring bryozoans, which could still harbor a large diversity of boring bryozoans and other boring taxa.

## 5 CONCLUSION

This study provides the most comprehensive up‐to‐date phylogeny of ctenostome bryozoans, including representatives of all commonly accepted ctenostome superfamilies. It corroborates the paraphyletic status of “Ctenostomata” by the inclusion of cheilostomes as the sister taxon of a clade comprising Walkerioidea, Victorellidae, Hislopioidea and Vesicularioidea. Furthermore, this study gives the first molecular support for a ctenostome affiliation of Penetrantiidae and reveals a potential sister‐group relationship to a clade containing *P. articulata*, *Arachnidium* sp. and *Terebripora* sp. However, additional morphological investigations, particularly on *P. pentagonalis* are essential to better understand this close relationship. The same holds true for *Terebripora*, since its arachnidioid affiliation is still surprising, and morphological information about this family are urgently required, not only to better understand its phylogeny but also to solve the ichnotaxonomic issue.

We also unraveled two cryptic species complexes, one in the North Sea and Northern Atlantic, and the *parva* complex in the Southern Pacific. Additionally, we confirm and describe *P. japonica* sp. nov. as a new species from Japan. Given the cryptic nature of these endolithic bryozoans, their potential diversity is expected to be much higher with many more boring species awaiting their discovery.

Moreover, our study proposes a monophyletic nature of Alcyonidioidea, with the monophyletic Multiporata nesting firmly within the latter, including the family Sundanellidae. However, sequence data of the multiporate genus *Elzerina*, would be essential to completely confirm the monophyly of Multiporata. Since this study provides the first complete mt genomes of 27 different ctenostomes it contributes to recent and future studies on this cryptic group of bryozoans. Although, we cover most ctenostome superfamilies, some of them are underrepresented und future studies should include more species to better understand their interrelationships. This holds particularly true for: Arachnidioidea, Walkerioidea, Benedeniporoidea and the families Hislopiidae, Victorellidae and Alcyonidiidae. Once sequence data of all recent endolithic families is available, a future study should combine all data to infer how often this lifestyle evolved independently within ctenostomes.

## AUTHOR CONTRIBUTIONS

**Sebastian H. Decker:** Conceptualization (equal); data curation (lead); investigation (lead); methodology (lead); software (equal); visualization (lead); writing – original draft (lead); writing – review and editing (lead). **Ahmed J. Saadi:** Methodology (equal); software (lead); visualization (equal); writing – review and editing (supporting). **Christian Baranyi:** Methodology (equal); writing – original draft (supporting); writing – review and editing (supporting). **Masato Hirose:** Investigation (supporting); methodology (supporting); resources (supporting); writing – original draft (supporting); writing – review and editing (supporting). **Sarah Lemer:** Formal analysis (supporting); investigation (supporting); writing – original draft (supporting); writing – review and editing (supporting). **Andy Sombke:** Methodology (supporting); resources (equal); writing – original draft (supporting); writing – review and editing (supporting). **Felipe Aguilera:** Data curation (supporting); investigation (supporting); methodology (supporting); resources (supporting); writing – original draft (supporting); writing – review and editing (supporting). **Leandro M. Vieira:** Data curation (supporting); investigation (supporting); methodology (supporting); resources (supporting); writing – original draft (supporting); writing – review and editing (supporting). **Abigail M. Smith:** Data curation (supporting); investigation (supporting); resources (supporting); supervision (supporting); validation (equal); writing – original draft (supporting); writing – review and editing (supporting). **Andrea Waeschenbach:** Formal analysis (supporting); investigation (supporting); methodology (supporting); software (supporting); writing – original draft (supporting); writing – review and editing (supporting). **Thomas Schwaha:** Conceptualization (equal); data curation (equal); investigation (supporting); methodology (supporting); supervision (equal); writing – original draft (supporting); writing – review and editing (supporting).

## CONFLICT OF INTEREST STATEMENT

All authors declare no conflict of interest.

## Supporting information


Appendix S1.

## Data Availability

All sequence data and raw reads used in this study can be found on GenBank (NCBI), the corresponding accession numbers are listed in Table 1 and Table A2. The alignment and tree files are deposited on figshare and publicly available. Single gene alignment before and after trimming: https://doi.org/10.6084/m9.figshare.25398193.v1. The concatenated alignment: https://doi.org/10.6084/m9.figshare.25398199.v1. The ML tree file: https://doi.org/10.6084/m9.figshare.25382560.v1 and the BI tree file: https://doi.org/10.6084/m9.figshare.25398187.v1.

## APPENDIX A

**TABLE A1:** PCR primers used for PCR amplification.

Name	Sequence 5′ ‐3′			Tann	Reference
LCO1490	GGTCAACAAATCATAAAGATATTGG	F	40 cycles	45°C	Folmer et al. (1994)
HCO2198	TAAACTTCAGGGTGACCAAAAAATCA	R		45°C	Folmer et al. (1994)
boring_coF	TCAACTAACCATAAAGACATTGG	F		48°C	This study
penet_coF	ATGTCAACTAACCATAAAGACATTGGCA	F		49°C	This study
penet_coR	TAGACTTCTGGGTGTCCGAAGAATCA	R		49°C	This study

**TABLE A2:** Species and genes of the data matrix used in the phylogenetic reconstruction shown in Figures 1, 2 and A2.

Internal code	GenBank accession number				Species	cox1	cox2	cox3	Cob	nad1	nad2	nad3	nad4	nad4l	nad5	nad6	atp6	atp8	rrnS 12S	rrnL 16S	18S	28S
	Mt genome	18 S	28 S	Raw																		
arach‐FR22‐78A	OR620116	OR625474	OR625501	SRR28306760	*Arachnidium* sp.	1	1	1	1	1	1	1	1	1	1	1	1	1	1	1	1	1
tere‐CH22‐4A	OR620138	OR625496	OR625523	SRR28306759	*Terebripora* sp.	1	1	1	1	1	1	1	1	1	1	1	1	1	1	1	1	1
palu‐Lax22	OR620117	OR625475	OR625502	SRR28306746	*Paludicella articulata*	1	1	1	1	1	1	1	1	1	1	1	1	1	1	1	1	1
penet_NZ19_NL9_A	OR620118	OR625476	OR625503	SRR28306740	*Penetrantia parva* North	1	1	1	1	1	1	1	1	1	1	1	1	1	1	1	1	1
penet_NZ21_PB3_PARVA	OR620119	OR625477	OR625504	SRR28306739	*Penetrantia* cf. *parva* South	1	1	1	1	1	1	1	1	1	1	1	1	1	1	1	1	1
penet_CH22_25A	OR620120	OR625478	OR625505	SRR28306738	*Penetrantia* cf. *parva*	1	1	1	1	1	1	1	1	1	1	1	1	1	1	1	1	1
penet_Guam21_1A	OR620121	OR625479	OR625506	SRR28306737	*Penetrantia clionoides*	1	1	1	1	1	1	1	1	1	1	1	1	1	1	1	1	1
penet_NZ21_PB3_IRREG	OR620122	OR625480	OR625507	SRR28306736	*Penetrantia irregularis*	1	1	1	1	1	1	1	1	1	1	1	1	1	1	1	1	1
penet_Sk19_39A	OR620123	OR625481	OR625508	SRR28306735	*Penetrantia concharum*	1	1	1	1	1	1	1	1	1	1	1	1	1	1	1	1	1
penet_FR20_6A	OR620124	OR625482	OR625509	SRR28306734	*Penetrantia concharum*	1	1	1	1	1	1	1	1	1	1	1	1	1	1	1	1	1
penet_FR21_35A	OR620125	OR625483	OR625510	SRR28372652	*Penetrantia* sp.	1	1	1	1	1	1	1	1	1	1	1	1	1	1	1	1	1
penet_JT20_10A	OR620126	OR625484	OR625511	SRR28306757	*Penetrantia japonica* sp. nov.	1	1	1	1	1	1	1	1	1	1	1	1	0	1	1	1	1
alcy‐FR22‐62A	OR620127	OR625485	OR625512	SRR28306756	*Alcyonidium polyoum*	1	1	1	1	1	1	1	1	1	1	1	1	0	1	1	1	1
alcym‐FR22‐57A	OR620128	OR625486	OR625513	SRR28306755	*Alcyonidium gelatinosum*	1	1	1	1	1	1	1	1	1	1	1	1	0	1	1	1	1
pher‐SP19‐2A	OR620129	OR625487	OR625514	SRR28306754	*Pherusella liowae*	1	1	1	1	1	1	1	1	1	1	1	1	0	1	1	1	1
flus‐FR22‐61A	OR620130	OR625488	OR625515	SRR28306753	*Flustrellidra hispida*	1	1	1	1	1	1	1	1	1	1	1	1	1	1	1	1	1
sund‐SP19‐1A	OR620131	OR625489	OR625489	SRR28306752	*Sundanella sibogae*	1	1	1	1	1	1	1	1	1	1	1	1	1	1	1	1	1
sund‐Brazil22‐3	OR620132	OR625490	OR625517	SRR28306751	*S*. sibogae	1	1	1	1	1	1	1	1	1	1	1	1	1	1	1	1	1
aever‐Brazil22‐2	OR620133	OR625491	OR625518	SRR28306750	*Aeverrillia setigera*	1	1	1	1	1	1	1	1	1	1	1	1	1	1	1	1	1
his‐Thai20‐8A	OR620134	OR625492	OR625519	SRR28306749	*Hislopia malayensis*	1	1	1	1	1	1	1	1	1	1	1	1	1	1	1	1	1
tanga‐GRW22‐5A	OR620135	OR625493	OR625520	SRR28306747	*Tanganella muelleri*	1	1	1	1	1	1	1	1	1	1	1	1	0	1	1	1	1
bulb‐GRW22‐1A	OR620136	OR625494	OR625521	SRR28306746	*Bulbella abscondita*	1	1	1	1	1	1	1	1	1	1	1	1	1	1	1	1	1
amphi‐NIWA04‐1	OR620137	OR625495	OR625522	SRR28306745	*Amphibiobeania epiphylla*	1	1	1	1	1	1	1	1	1	1	1	1	1	1	1	1	1
bower‐GRW22‐11A	OR620139	OR625497	OR625524	SRR28306744	*Amathia gracilis*	1	1	1	1	1	1	1	1	1	1	1	1	1	1	1	1	1
amathd‐Brazil22‐4	OR620140	OR625498	OR625525	SRR28306743	*Amathia distans*	1	1	1	1	1	1	1	1	1	1	1	1	1	1	1	1	1
amath‐Brazil22‐1	OR620141	OR625499	OR625526	SRR28306742	*Amathia ernsti*	1	1	1	1	1	1	1	1	1	1	1	1	0	1	1	1	1
vesi‐FR22‐66A	OR620142	OR625500	OR625527	SRR28306741	*Vesicularia spinosa*	1	1	1	1	1	1	1	1	1	1	1	1	0	1	1	1	1
Mono1	—	—	—	SRR25585373	*Monobryozoon ambulans*	1	1	1	1	1	1	1	1	1	1	1	1	1	1	1	1	1
BLEED819_2018	MT293123	MT311330	MT311474	‐	*Electra pilosa*	1	1	1	1	1	1	1	1	1	1	1	1	0	1	1	1	1
BLEED601	MW159977	MW13482	MW173683	‐	*Aetea* sp.	1	1	1	1	1	1	1	1	1	1	1	1	1	1	1	1	1
BLEED816_2018	MT293110	MT311337	MT311481	‐	*Membranipora membranacea*	1	1	1	1	1	1	1	1	1	1	1	1	1	1	1	1	1
BLEED1112	MW160020	MW134983	MW173826	—	*Schizomavella aotearoa*	1	1	1	1	1	1	1	1	1	1	1	1	1	1	1	1	1
BLEED1666	MW160113	MW134930	MW173777	‐	*Metroperiella* sp.	1	1	1	1	1	1	1	1	1	1	1	1	1	1	1	1	1
BLEED1362	MW160074	MW134947	MW173791	‐	*Mucropetraliella vultur*	1	1	1	1	1	1	1	1	1	1	1	1	0	1	1	1	1
BLEED1631	MW160100	MW134855	MW173710	‐	*Cellaria immersa*	1	1	1	1	1	1	1	1	1	1	1	1	1	1	1	1	1
BLEED560	MT293086	MT311322	MT311467	‐	*Bicellariella ciliata*	1	1	1	1	1	1	1	1	1	1	1	1	1	1	1	1	1
BLEED1625	MW160096	MW134837	MW173695	‐	*Beania serrata*	1	1	1	1	1	1	1	1	1	1	1	1	1	1	1	1	1
‐	NC_038192	FJ196124	FJ409576	‐	*Pectinatella magnifica*	1	1	1	1	1	1	1	1	1	1	1	1	1	1	1	1	1

*Note*: 1—recovered; , recoverd with exonerate; , not included.

**TABLE A3:** Estimates of evolutionary divergence between nine *Penetrantia* specimens based on the cox1 gene full length.

	*P*. cf. *parva* – Chile	*P. concharum* – France	*Penetrantia* sp. – France	*P. clionoides*–‐ Guam	*P.japonica* sp. nov. – Japan	*P. parva* – NZ north	*P. irregularis* – NZ south	*P*. cf. *parva* – NZ south	*P. concharum* – Sweden
*P. parva* ‐ Chile									
*P. concharum* – France	0.227								
*Penetrantia* sp. – France	0.231	0.219							
*P. clionoides* – Guam	0.146	0.233	0.232						
*P. japonica* sp. nov. – Japan	0.298	0.292	0.281	0.291					
*P. parva* – NZ North	0.129	0.222	0.232	0.154	0.302				
*P. irregularis* – NZ South	0.241	0.226	0.220	0.234	0.302	0.225			
*P*. cf. *parva* – NZ South	0.098	0.225	0.242	0.136	0.298	0.121	0.231		
*P. concharum* – Sweden	0.226	0.003	0.216	0.232	0.291	0.221	0.225	0.224	

*Note*: The number of base substitutions per site between sequences are shown. Analyses were conducted using the Maximum Composite Likelihood model (Tamura et al., 2004). The rate variation among sites was modeled with a gamma distribution (shape parameter = 1). All ambiguous positions were removed for each sequence pair (pairwise deletion option). There were a total of 1506 positions in the final dataset. Evolutionary analyses were conducted in MEGA11 (Tamura et al., 2021).

**TABLE A4:** Estimates of evolutionary divergence between ten *Penetrantia* specimens including Helgoland, Germany based on the cox1 gene barcoding region.

	*P*. cf. *parva* – Chile	*P. concharum* – France	*Penetrantia* sp. – France	*P. clionoides* – Guam	*P. japonica* sp. nov. – Japan	*P. parva* – NZ north	*P. irregularis* – NZ south	*P*. cf. *parva* – NZ south	*P. concharum* – Sweden	*Penetrantia* sp. – Helgoland
*P. parva* ‐ Chile										
*P. concharum* – France	0.378									
*Penetrantia* sp. ‐ France	0.371	0.346								
*P. clionoides* – Guam	0.212	0.384	0.371							
*P. japonica* sp. nov. – Japan	0.533	0.518	0.484	0.513						
*P. parva* – NZ North	0.189	0.368	0.371	0.232	0.537					
*P. irregularis* – NZ South	0.399	0.374	0.349	0.389	0.536	0.370				
*P*. cf. *parva* – NZ South	0.140	0.369	0.394	0.196	0.533	0.179	0.382			
*P. concharum* ‐ Sweden	0.376	0.004	0.341	0.383	0.516	0.366	0.371	0.368		
*Penetrantia* sp. – Helgoland	0.352	0.280	0.023	0.361	0.475	0.371	0.311	0.344	0.269	

*Note*: The number of base substitutions per site between sequences is shown. Analyses were conducted using the Maximum Composite Likelihood model (Tamura et al., 2004). The rate variation among sites was modeled with a gamma distribution (shape parameter = 1). All ambiguous positions were removed for each sequence pair (pairwise deletion option). There was a total of 640 positions in the final dataset. Evolutionary analyses were conducted in MEGA11 (Tamura et al., 2021).

## References

[ece311276-bib-0001] Allman, G. J. (1856). A monograph of the fresh‐water Polyzoa. (Vol. 28). Ray Society.

[ece311276-bib-0002] Altschul, S. F. , Gish, W. , Miller, W. , Myers, E. W. , & Lipman, D. J. (1990). Basic local alignment search tool. Journal of Molecular Biology, 215(3), 403–410. 10.1016/S0022-2836(05)80360-2 2231712

[ece311276-bib-0003] Annandale, N. (1916). Zoological results of a tour in the Far East. Polyzoa, Entoprocta, and Ctenostomata. Memoirs of the Asiatic Society of Bengal, 6, 13–37.

[ece311276-bib-0004] Bankevich, A. , Nurk, S. , Antipov, D. , Gurevich, A. A. , Dvorkin, M. , Kulikov, A. S. , Lesin, V. M. , Nikolenko, S. I. , Pham, S. , Prjibelski, A. D. , Pyshkin, A. V. , Sirotkin, A. V. , Vyahhi, N. , Tesler, G. , Alekseyev, M. A. , & Pevzner, P. A. (2012). SPAdes: A new genome assembly algorithm and its applications to single‐cell sequencing. Journal of Computational Biology, 19(5), 455–477. 10.1089/cmb.2012.0021 22506599 PMC3342519

[ece311276-bib-0005] Banta, W. C. (1975). Origin and early evolution of cheilostome Bryozoa. In S. Pouyet (Ed.), Bryozoa 1974 (pp. 565–582). Université Claude Bernard.

[ece311276-bib-0006] Baptista, L. , Berning, B. , Curto, M. , Waeschenbach, A. , Meimberg, H. , Santos, A. M. , & Ávila, S. P. (2022). Morphospecies and molecular diversity of ‘lace corals’: The genus *Reteporella* (Bryozoa: Cheilostomatida) in the central North Atlantic Azores archipelago. BMC Ecology and Evolution, 22(1), 128. 10.1186/s12862-022-02080-z 36333666 PMC9635095

[ece311276-bib-0007] Bertling, M. , Braddy, S. J. , Bromley, R. G. , Demathieu, G. R. , Genise, J. , Mikuláš, R. , Nielsen, J. K. , Nielsen, K. S. S. , Rindsberg, A. K. , Schlirf, M. , & Uchman, A. (2006). Names for trace fossils: A uniform approach. Lethaia, 39(3), 265–286. 10.1080/00241160600787890

[ece311276-bib-0008] Bobin, G. , & Prenant, M. (1954). Sur un bryozaire perforant (*Terebripora* comma Soule), trouvé en Méditerranée. Archives de Zoologie Expérimentale et générale, 91, 130–144.

[ece311276-bib-0009] Bock, P. , & Gordon, D. P. (2013). Phylum Bryozoa Ehrenberg, 1831. Zootaxa, 3703, 67–74. 10.11646/zootaxa.4979.1.27 34186996

[ece311276-bib-0010] Borg, F. (1940). On the genus *Tubiporella* and a new boring bryozoan. Zoologiska Bidrag från Uppsala, 18, 415–437.

[ece311276-bib-0011] Borowiec, M. L. (2016). AMAS: A fast tool for alignment manipulation and computing of summary statistics. PeerJ, 28(4), e1660. 10.7717/peerj.1660 PMC4734057 26835189

[ece311276-bib-0012] Braem, F. (1951). Über *Victorella* und einige ihrer nächsten Verwandten, sowie über die Bryozoenfauna des Ryck bei Greifswald. Zoologica, 102, 1–59.

[ece311276-bib-0013] Capella‐Gutiérrez, S. , Silla‐Martínez, J. M. , & Gabaldón, T. (2009). trimAl: A tool for automated alignment trimming in large‐scale phylogenetic analyses. Bioinformatics, 25(15), 1972–1973. 10.1093/bioinformatics/btp348 19505945 PMC2712344

[ece311276-bib-0014] Chimenz Gusso, C. , Boccia, P. , & Giovannini, N. (2004). Importance of faunistic and taxonomical studies for a correct analysis of the zoogeography of Mediterranean Bryozoa. Biogeographia, 25, 93–108.

[ece311276-bib-0015] Çinar, M. E. , & Dagli, E. (2021). Bioeroding (boring) polychaete species (Annelida: Polychaeta) from the Aegean Sea (eastern Mediterranean). Journal of the Marine Biological Association of the United Kingdom, 101(2), 309–318. 10.1017/S002531542100031X

[ece311276-bib-0016] Collins, K. S. , Edie, S. M. , & Jablonski, D. (2023). Convergence and contingency in the evolution of a specialized mode of life: Multiple origins and high disparity of rock‐boring bivalves. Proceedings of the Royal Society B: Biological Sciences, 290(1992), 20221907. 10.1098/rspb.2022.1907 PMC9904949 36750185

[ece311276-bib-0017] Criscuolo, A. , & Gribaldo, S. (2010). BMGE (block mapping and gathering with entropy): A new software for selection of phylogenetic informative regions from multiple sequence alignments. BMC Evolutionary Biology, 10(1), 210. 10.1186/1471-2148-10-210 20626897 PMC3017758

[ece311276-bib-0018] Darriba, D. , Posada, D. , Kozlov, A. M. , Stamatakis, A. , Morel, B. , & Flouri, T. (2019). ModelTest‐NG: A new and scalable tool for the selection of DNA and protein evolutionary models. Molecular Biology and Evolution, 37(1), 291–294. 10.1093/molbev/msz189 PMC6984357 31432070

[ece311276-bib-0019] Decker, S. , Wanninger, A. , & Schwaha, T. (2020). Morphology and life cycle of an epiphytic pherusellid ctenostome bryozoan from the Mediterranean Sea. Organisms Diversity & Evolution, 20, 417–437. 10.1007/s13127-020-00443-2

[ece311276-bib-0020] Decker, S. H. , Gordon, D. P. , Spencer Jones, M. E. , & Schwaha, T. (2021). A revision of the ctenostome bryozoan family Pherusellidae, with description of two new species. Journal of Zoological Systematics and Evolutionary Research, 59(5), 963–980. 10.1111/jzs.12466

[ece311276-bib-0021] Decker, S. H. , Hirose, M. , Lemer, S. , Kuklinski, P. , Spencer, H. G. , Smith, A. M. , & Schwaha, T. (2023). Boring bryozoans: An investigation into the endolithic bryozoan family Penetrantiidae. Organisms, Diversity and Evolution, 23, 743–785. 10.1007/s13127-023-00612-z PMC10689564 38046835

[ece311276-bib-0022] D'Hondt, J. L. (1983). Tabular keys for identification of the recent Ctenostomatous Bryozoa. Mémoires de L'Institut Océanographique, Monaco, 14, 1–134.

[ece311276-bib-0023] Donath, A. , Jühling, F. , Al‐Arab, M. , Bernhart, S. H. , Reinhardt, F. , Stadler, P. F. , Middendorf, M. , & Bernt, M. (2019). Improved annotation of protein‐coding genes boundaries in metazoan mitochondrial genomes. Nucleic Acids Research, 47(20), 10543–10552. 10.1093/nar/gkz833 31584075 PMC6847864

[ece311276-bib-0024] d'Orbigny, A. (1847). Voyage dans l'Amérique méridionale Vol. 5, part 4: Zoophytes. B. Bertrand, V. Levrault.

[ece311276-bib-0025] Ehlers, E. (1876). *Hypophorella expansa*, ein Beitrag zur Kenntnis der minierenden Bryozoen. Abhandlungen der Koeniglichen Gesellschaft der Wissenschaften Zu Goettingen, 21, 1–156.

[ece311276-bib-0026] Ehrenberg, C. G. (1831). Symbolae Physicae, seu Icones etDescription Mammalium, Avium, Insectorum et Animalium Evertebratorum. Ex Officina Academica, Berlin .

[ece311276-bib-0027] Fehlauer‐Ale, K. H. , Mackie, J. A. , Lim‐Fong, G. E. , Ale, E. , Pie, M. R. , & Waeschenbach, A. (2014). Cryptic species in the cosmopolitan *Bugula neritina* complex (Bryozoa, Cheilostomata). Zoologica Scripta, 43(2), 193–205. 10.1111/zsc.12042

[ece311276-bib-0028] Folmer, O. , Black, M. , Hoeh, W. , Lutz, R. , & Vrijenhoek, R. (1994). DNA primers for amplification of mitochondrial cytochrome *c* oxidase subunit I from diverse metazoan invertebrates. Molecular Marine Biology and Biotechnology, 3(5), 294–299. [Research Support, Non‐U S Gov't]. 7881515

[ece311276-bib-0029] Fuchs, J. , Obst, M. , & Sundberg, P. (2009). The first comprehensive molecular phylogeny of Bryozoa (*Ectoprocta*) based on combined analyses of nuclear and mitochondrial genes. Molecular Phylogenetics and Evolution, 52(1), 225–233. 19475710 10.1016/j.ympev.2009.01.021

[ece311276-bib-0030] Gim, J.‐S. , Ko, E.‐J. , Kim, H.‐G. , Kim, Y.‐M. , Hong, S. , Kim, H.‐W. , Gim, J. A. , Joo, G. J. , & Jo, H. (2018). Complete mitochondrial genome of the freshwater bryozoan *Pectinatella magnifica* (Phylactolaemata: Plumatellida) assembled from next‐generation sequencing data. Mitochondrial DNA Part B Resources, 3(1), 373–374. 10.1080/23802359.2018.1450657 33474173 PMC7799736

[ece311276-bib-0031] Gomez, A. , Wright, P. J. , Lunt, D. H. , Cancino, J. M. , & Hughes, R. N. (2007). Mating trials validate the use of DNA barcoding to reveal cryptic speciation of a marine bryozoan taxon. Proceedings of the Royal Society B: Biological Sciences, 274(1607), 199–207. 10.1098/rspb.2006.3718 PMC1685843 17035167

[ece311276-bib-0032] Gouy, M. , Guindon, S. , & Gascuel, O. (2010). SeaView version 4: A multiplatform graphical user interface for sequence alignment and phylogenetic tree building. Molecular Biology and Evolution, 27(2), 221–224. 19854763 10.1093/molbev/msp259

[ece311276-bib-0033] Greiner, S. , Lehwark, P. , & Bock, R. (2019). OrganellarGenomeDRAW (OGDRAW) version 1.3.1: Expanded toolkit for the graphical visualization of organellar genomes. Nucleic Acids Research, 47(W1), W59–W64. 10.1093/nar/gkz238 30949694 PMC6602502

[ece311276-bib-0034] Grischenko, A. V. , Hirose, M. , Schwaha, T. , & Chernyshev, A. V. (2019). First record of an abyssal and hadal bryozoan fauna from the Kuril‐Kamchatka trench. Progress in Oceanography, 176, 102130. 10.1016/j.pocean.2019.102130

[ece311276-bib-0035] Gruhl, A. (2020). Larval structure and metamorphosis. In T. Schwaha (Ed.), Handbook of zoology, Bryozoa (pp. 123–142). de Gruyter.

[ece311276-bib-0036] Harmer, S. F. (1915). The polyzoa of the Siboga expedition. Part 1. Entoprocta, Ctenostomata and Cyclostomata. Siboga Expeditie, 28 A, 1–180.

[ece311276-bib-0037] Hayward, P. J. (1985). Ctenostome Bryozoans. E.J. Brill/Dr.W. Backhuys for The Linnean Society of London & The Estuarine and Brackish‐Water Siences Association.

[ece311276-bib-0038] Jebram, D. (1973). Stolonen‐Entwicklung und Systematik bei den Bryozoa Ctenostomata. Journal of Zoological Systematics and Evolutionary Research, 11, 1–48.

[ece311276-bib-0040] Jebram, D. (1986). The ontogenetical and supposed phylogenetical fate of the parietal muscles in the Ctenostomata (Bryozoa). Journal of Zoological Systematics and Evolutionary Research, 24, 58–82.

[ece311276-bib-0041] Katoh, K. , Kuma, K.‐I. , Toh, H. , & Miyata, T. (2005). MAFFT version 5: Improvement in accuracy of multiple sequence alignment. Nucleic Acids Research, 33(2), 511–518. 10.1093/nar/gki198 15661851 PMC548345

[ece311276-bib-0042] Katoh, K. , Misawa, K. , Kuma, K. , & Miyata, T. (2002). MAFFT: A novel method for rapid multiple sequence alignment based on fast Fourier transform. Nucleic Acids Research, 30(14), 3059–3066. 10.1093/nar/gkf436 12136088 PMC135756

[ece311276-bib-0043] Katoh, K. , & Standley, D. M. (2013). MAFFT multiple sequence alignment software version 7: Improvements in performance and usability. Molecular Biology and Evolution, 30(4), 772–780. 10.1093/molbev/mst010 23329690 PMC3603318

[ece311276-bib-0044] Kozlov, A. M. , Darriba, D. , Flouri, T. , Morel, B. , & Stamatakis, A. (2019). RAxML‐NG: A fast, scalable and user‐friendly tool for maximum likelihood phylogenetic inference. Bioinformatics, 35(21), 4453–4455. 10.1093/bioinformatics/btz305 31070718 PMC6821337

[ece311276-bib-0045] Kumar, S. , Nei, M. , Dudley, J. , & Tamura, K. (2008). MEGA: A biologist‐centric software for evolutionary analysis of DNA and protein sequences. Briefings in Bioinformatics, 9(4), 299–306. 10.1093/bib/bbn017 18417537 PMC2562624

[ece311276-bib-0046] Lagesen, K. , Hallin, P. , Rødland, E. A. , Stærfeldt, H.‐H. , Rognes, T. , & Ussery, D. W. (2007). RNAmmer: Consistent and rapid annotation of ribosomal RNA genes. Nucleic Acids Research, 35(9), 3100–3108. 10.1093/nar/gkm160 17452365 PMC1888812

[ece311276-bib-0047] Marcus, E. (1937). Briozoarios marinhos brasileiros I. Boletim da Faculdade de Filosofia, ciéncias e Letras, 1, 1–224.

[ece311276-bib-0048] Marcus, E. (1941). Sobre Bryozoa do Brasil. I. Boletim da Faculdade de Filosofia, ciéncias e Letras, 5, 3–208.

[ece311276-bib-0049] Metcalfe, K. , Gordon, D. P. , & Hayward, E. (2007). An amphibious bryozoan from living mangrove leaves ‐ *Amphibiobeania* new genus (Beaniidae). Zoological Science, 24, 563–570. 17867857 10.2108/zsj.24.563

[ece311276-bib-0050] Meyer, C. P. , & Paulay, G. (2005). DNA barcoding: Error rates based on comprehensive sampling. PLoS Biology, 3(12), e422. 10.1371/journal.pbio.0030422 16336051 PMC1287506

[ece311276-bib-0051] Mukai, H. , Terakado, K. , & Reed, C. G. (1997). Bryozoa. In F. W. Harrison & R. M. Woollacott (Eds.), Microscopic anatomy of invertebrates (Vol. 13 (pp. 45–206). Wiley‐Liss.

[ece311276-bib-0052] Nascimento, F. F. , Reis, M. , & Yang, Z. (2017). A biologist's guide to Bayesian phylogenetic analysis. Nature Ecology & Evolution, 1(10), 1446–1454. 10.1038/s41559-017-0280-x 28983516 PMC5624502

[ece311276-bib-0053] Orr, R. J. S. , Di Martino, E. , Gordon, D. P. , Ramsfjell, M. H. , Mello, H. L. , Smith, A. M. , & Liow, L. H. (2021). A broadly resolved molecular phylogeny of New Zealand cheilostome bryozoans as a framework for hypotheses of morphological evolution. Molecular Phylogenetics and Evolution, 161, 107172. 10.1016/j.ympev.2021.107172 33813020

[ece311276-bib-0054] Orr, R. J. S. , Di Martino, E. , Ramsfjell, M. H. , Gordon, D. P. , Berning, B. , Chowdhury, I. , Craig, S. , Cumming, R. L. , Figuerola, B. , Florence, W. , Harmelin, J. G. , Hirose, M. , Huang, D. , Jain, S. S. , Jenkins, H. L. , Kotenko, O. N. , Kuklinski, P. , Lee, H. E. , Madurell, T. , … Liow, L. H. (2022). Paleozoic origins of cheilostome bryozoans and their parental care inferred by a new genome‐skimmed phylogeny. Science Advances, 8(13), eabm7452. 10.1126/sciadv.abm7452 35353568 PMC8967238

[ece311276-bib-0055] Orr, R. J. S. , Haugen, M. N. , Berning, B. , Bock, P. , Cumming, R. L. , Florence, W. K. , Hirose, M. , Di Martino, E. , Ramsfjell, M. H. , Sannum, M. M. , Smith, A. M. , Vieira, L. M. , Waeschenbach, A. , & Liow, L. H. (2019). A genome‐skimmed phylogeny of a widespread bryozoan family, Adeonidae. BMC Evolutionary Biology, 19(1), 235. 10.1186/s12862-019-1563-4 31881939 PMC6935126

[ece311276-bib-0056] Pohowsky, R. A. (1978). The boring Ctenostomate Bryozoa: Taxonomy and paleobiology based on cavities in calcareous substrata. Bulletins of American Paleontology, 73, 1–192.

[ece311276-bib-0057] Pröts, P. , Wanninger, A. , & Schwaha, T. (2019). Life in a tube: Morphology of the ctenostome bryozoan *Hypophorella expansa* . Zoological Letters, 5(1), 28. 10.1186/s40851-019-0142-2 31410295 PMC6686267

[ece311276-bib-0058] Reed, C. G. (1991). Bryozoa. In A. C. Giese , J. S. Pearse , & V. B. Pearse (Eds.), Reproduction of marine invertebrates. VI. Echinoderms and Lophophorates (pp. 85–245). The Boxwood Press.

[ece311276-bib-0059] Remane, A. (1936). *Monobryozoon ambulans* n. gen., n. sp., ein eigenartiges Bryozoon des Meeressandes. Zoologischer Anzeiger, 113, 161–167.

[ece311276-bib-0060] Reverter‐Gil, O. , D'Hondt, J. L. , & Fernandez Pulpeiro, E. (1995). Mise à jour de l'inventaire des Bryozoaires de Roscoff publié par Echalier et Prenant (1951). Cahiers de Biologie Marine, 36, 123–131.

[ece311276-bib-0061] Reverter‐Gil, O. , & Souto, J. (2014). Annotated checklist of recent marine Bryozoa from continental Portugal. Nova Acta Científica Compostelana (Bioloxía), 21, 1–55.

[ece311276-bib-0062] Reverter‐Gil, O. , Souto, J. , & Fernández Pulpeiro, E. (2016). Fauna Iberica. Vol 43. Bryozoa 1. Ctenostomata. Museo Nacional de Ciencias Naturales.

[ece311276-bib-0063] Rogick, M. D. , & Brown, C. J. D. (1942). Studies on fresh‐water Bryozoa. 12. A collection from various sources. Annals of the New York Academy of Sciences, 43, 123–144.

[ece311276-bib-0064] Ronquist, F. , & Huelsenbeck, J. P. (2003). MrBayes 3: Bayesian phylogenetic inference under mixed models. Bioinformatics, 19(12), 1572–1574. 10.1093/bioinformatics/btg180 12912839

[ece311276-bib-0065] Rosso, A. (2008). *Leptichnus tortus* isp. Nov., a new cheilostome etching and comments on other bryozoan‐produced trace fossils. Studi Trentini di Scienze Naturali/Acta Geol, 83, 75–85.

[ece311276-bib-0066] Ryland, J. S. (1970). Bryozoans. Hutchinson University Library.

[ece311276-bib-0067] Saadi, A. J. , Bibermair, J. , Kocot, K. M. , Roberts, N. G. , Hirose, M. , Calcino, A. , Baranyi, C. , Chaichana, R. , Wood, T. S. , & Schwaha, T. (2022). Phylogenomics reveals deep relationships and diversification within phylactolaemate bryozoans. Proceedings of the Royal Society B: Biological Sciences, 289(1986), 20221504. 10.1098/rspb.2022.1504 PMC9653232 36350215

[ece311276-bib-0068] Schack, C. R. , Gordon, D. P. , & Ryan, K. G. (2019). Modularity is the mother of invention: A review of polymorphism in bryozoans. Biological Reviews, 94, 773–809. 10.1111/brv.12478 30450650

[ece311276-bib-0069] Schwaha, T. (2020a). General introduction. In T. Schwaha (Ed.), Handbook of zoology, Bryozoa (pp. 1–10). De Gruyter.

[ece311276-bib-0070] Schwaha, T. (2020b). Morphology of bryozoans. In T. Schwaha (Ed.), Handbook of zoology: Bryozoa (pp. 57–100). DeGruyter.

[ece311276-bib-0071] Schwaha, T. (2020c). Ctenostomata. In T. Schwaha (Ed.), Handbook of zoology. Bryozoa (pp. 269–316). De Gruyter.

[ece311276-bib-0072] Schwaha, T. (2020d). Gymnolaemata. In T. Schwaha (Ed.), Handbook of zoology: Bryozoa (pp. 265–268). de Gruyter.

[ece311276-bib-0073] Schwaha, T. (2021). Morphology of ctenostome bryozoans. 3. *Elzerina*, *Flustrellidra*, Bockiella. Journal of Morphology, 282, 633–651. 33576505 10.1002/jmor.21334 PMC8048840

[ece311276-bib-0074] Schwaha, T. , & De Blauwe, H. (2020). Morphology of ctenostome bryozoans: 1. *Arachnidium fibrosum* . Journal of Morphology, 281(12), 1598–1606. 10.1002/jmor.21275 33009880 PMC7756562

[ece311276-bib-0075] Schwaha, T. , Decker, S. H. , Baranyi, C. , & Saadi, A. J. (2024). Rediscovering the unusual, solitary bryozoan *Monobryozoon ambulans* Remane, 1936: First molecular and new morphological data clarify its phylogenetic position. Frontiers in Zoology, 21(1), 5. 10.1186/s12983-024-00527-1 38443908 PMC10913646

[ece311276-bib-0076] Schwaha, T. , Ruthensteiner, B. , Melzer, R. R. , Asami, T. , & Páll‐Gergely, B. (2019). Three phlya ‐ two type specimens ‐ one shell: History of a snail shell revealed by modern imaging technology. Journal of Zoological Systematics and Evolutionary Research, 57, 527–533.

[ece311276-bib-0077] Schwaha, T. , Waeschenbach, A. , De Blauwe, H. , & Gordon, D. P. (2022). Morphology of ctenostome bryozoans: 6. *Amphibiobeania epiphylla* . Journal of Morphology, 283(12), 1505–1516. 10.1002/jmor.21519 36205214 PMC9828531

[ece311276-bib-0078] Schwaha, T. , Winston, J. E. , & Gordon, D. P. (2022). Morphology of ctenostome bryozoans: 5. Sundanella, with description of a new species from the Western Atlantic and the Multiporata concept. Journal of Morphology, 283(9), 1139–1162. 10.1002/jmor.21494 35788975 PMC9545146

[ece311276-bib-0079] Seo, J. E. , Chae, H. S. , Winston, J. E. , Zagorsek, K. , & Gordon, D. P. (2018). Korean ctenostome bryozoans‐observations on living colonies, new records, five new species, and an updated checklist. Zootaxa, 4486(3), 251–283. 10.11646/zootaxa.4486.3.3 30313746

[ece311276-bib-0080] Silén, L. (1946). On two new groups of Bryozoa living in the shells of molluscs. Arkiv för Zoologi, 38B, 1–7.

[ece311276-bib-0081] Silén, L. (1947). On the anatomy and biology of Penetrantiidae and Immergentiidae (Bryozoa). Arkiv för Zoologi, 40A, 1–48.

[ece311276-bib-0082] Slater, G. S. C. , & Birney, E. (2005). Automated generation of heuristics for biological sequence comparison. BMC Bioinformatics, 6(1), 31. 10.1186/1471-2105-6-31 15713233 PMC553969

[ece311276-bib-0083] Smyth, M. J. (1988). *Penetrantia clionoides*, sp. nov. (Bryozoa), a boring bryozoan in gastropod shells from Guam. Biological Bulletin, 174(3), 276–286.

[ece311276-bib-0084] Soule, D. F. (1950a). A new species of *Terebripora* from the Pacific (Bryozoa Ctenostomata). Journal of the Washington Academy of Sciences, 40(11), 378–381.

[ece311276-bib-0085] Soule, D. F. (1950b). Penetrantiidae and Immergentiidae from the Pacific (Bryozoa Ctenostomata). Transactions of the American Microscopical Society, 69, 359–367.

[ece311276-bib-0086] Soule, J. D. , & Soule, D. F. (1969). Systematics and biogeography of burrowing bryozoans. American Zoologist, 9, 791–802.

[ece311276-bib-0087] Soule, J. D. , & Soule, D. F. (1975). *Spathipora*, its anatomy and phylogenetic affinities. In S. Pouyet (Ed.), Bryozoa 1974 Vol. 3 (pp. 247–253). Documents des laboratoires de géologie de la Faculté des sciences de Lyon. Hors série.

[ece311276-bib-0088] Tamura, K. , Nei, M. , & Kumar, S. (2004). Prospects for inferring very large phylogenies by using the neighbor‐joining method. Proceedings of the National Academy of Sciences of the United States of America, 101(30), 11030–11035. 10.1073/pnas.0404206101 15258291 PMC491989

[ece311276-bib-0089] Tamura, K. , Stecher, G. , & Kumar, S. (2021). MEGA11: Molecular evolutionary genetics analysis version 11. Molecular Biology and Evolution, 38(7), 3022–3027. 10.1093/molbev/msab120 33892491 PMC8233496

[ece311276-bib-0090] Taylor, P. D. (1986). The ancestrula and early growth pattern in two primitive cheilostome bryozoans: *Pyripora catenularia* (Fleming) and *Pyriporopsis partlandensis* Pohowsky. Journal of Natural History, 20, 101–110.

[ece311276-bib-0091] Taylor, P. D. (1990). Bioimmured ctenostomes from the Jurassic and the origin of the cheilostome Bryozoa. Palaeontology, 33, 19–34.

[ece311276-bib-0092] Thorpe, J. P. , Beardmore, J. A. , & Ryland, J. S. (1978). Genetic evidence for cryptic speciation in the marine bryozoan *Alcyonidium gelatinosum* . Marine Biology, 49(1), 27–32.

[ece311276-bib-0093] Thorpe, J. P. , & Ryland, J. S. (1979). Cryptic speciation detected by biochemical geneteics in three ecologically important intertidal bryozoans. Estuarine and Coastal Marine Science, 8, 395–398.

[ece311276-bib-0094] Todd, C. D. , Lambert, W. J. , & Thorpe, J. P. (1998). The genetic structure of intertidal populations of two species of nudibranch molluscs with planktotrophic and pelagic lecithotrophic larval stages: Are pelagic larvae “for” dispersal? Journal of Experimental Marine Biology and Ecology, 228(1), 1–28. 10.1016/S0022-0981(98)00005-7

[ece311276-bib-0095] Todd, J. A. (2000). The central role of ctenostomes in bryozoan phylogeny. In A. Herrera Cubilla & J. B. C. Jackson (Eds.), Proceedings of the 11th international Bryozoology association conference (pp. 104–135). Smithsonian Tropical Research Institute.

[ece311276-bib-0096] Van Soest, R. W. M. , Boury‐Esnault, N. , Vacelet, J. , Dohrmann, M. , Erpenbeck, D. , De Voogd, N. J. , Santodomingo, N. , Vanhoorne, B. , Kelly, M. , & Hooper, J. N. A. (2012). Global diversity of sponges (Porifera). PLoS One, 7(4), e35105. 10.1371/journal.pone.0035105 22558119 PMC3338747

[ece311276-bib-0097] Vieira, L. M. , Migotto, A. E. , & Winston, J. E. (2014). Ctenostomatous Bryozoa from Sao Paulo, Brazil, with descriptions of twelve new species. Zootaxa, 3889(4), 485–524. 25544281 10.11646/zootaxa.3889.4.2

[ece311276-bib-0098] Waeschenbach, A. , Cox, C. J. , Littlewood, D. T. J. , Porter, J. S. , & Taylor, P. D. (2009). First molecular estimate of cyclostome bryozoan phylogeny confirms extensive homoplasy among skeletal characters used in traditional taxonomy. Molecular Phylogenetics and Evolution, 52(1), 241–251. 10.1016/j.ympev.2009.02.002 19236933

[ece311276-bib-0099] Waeschenbach, A. , Taylor, P. D. , & Littlewood, D. T. J. (2012). A molecular phylogeny of bryozoans. Molecular Phylogenetics and Evolution, 62(2), 718–735. 10.1016/j.ympev.2011.11.011 22126903

[ece311276-bib-0100] Waeschenbach, A. , Vieira, L. M. , Reverter‐Gil, O. , Souto‐Derungs, J. , Nascimento, K. B. , & Fehlauer‐Ale, K. H. (2015). A phylogeny of Vesiculariidae (Bryozoa, Ctenostomata) supports synonymization of three genera and reveals possible cryptic diversity. Zoologica Scripta, 44(6), 667–683. 10.1111/zsc.12130

[ece311276-bib-0101] Wisshak, M. , Knaust, D. , & Bertling, M. (2019). Bioerosion ichnotaxa: Review and annotatedlist. Facies, 65(2), 24. 10.1007/s10347-019-0561-8

