# Supplementary material for: Boring systematics: A genome skimmed phylogeny of ctenostome bryozoans and their endolithic family Penetrantiidae with the description of one new species
Source: Ecol Evol. 2024 Apr 18;14(4):e11276. doi: 10.1002/ece3.11276 (PMC11024686; doi:10.1002/ece3.11276)

Arachnidium sp. VIBE_Arach_FR22_78A OR620116

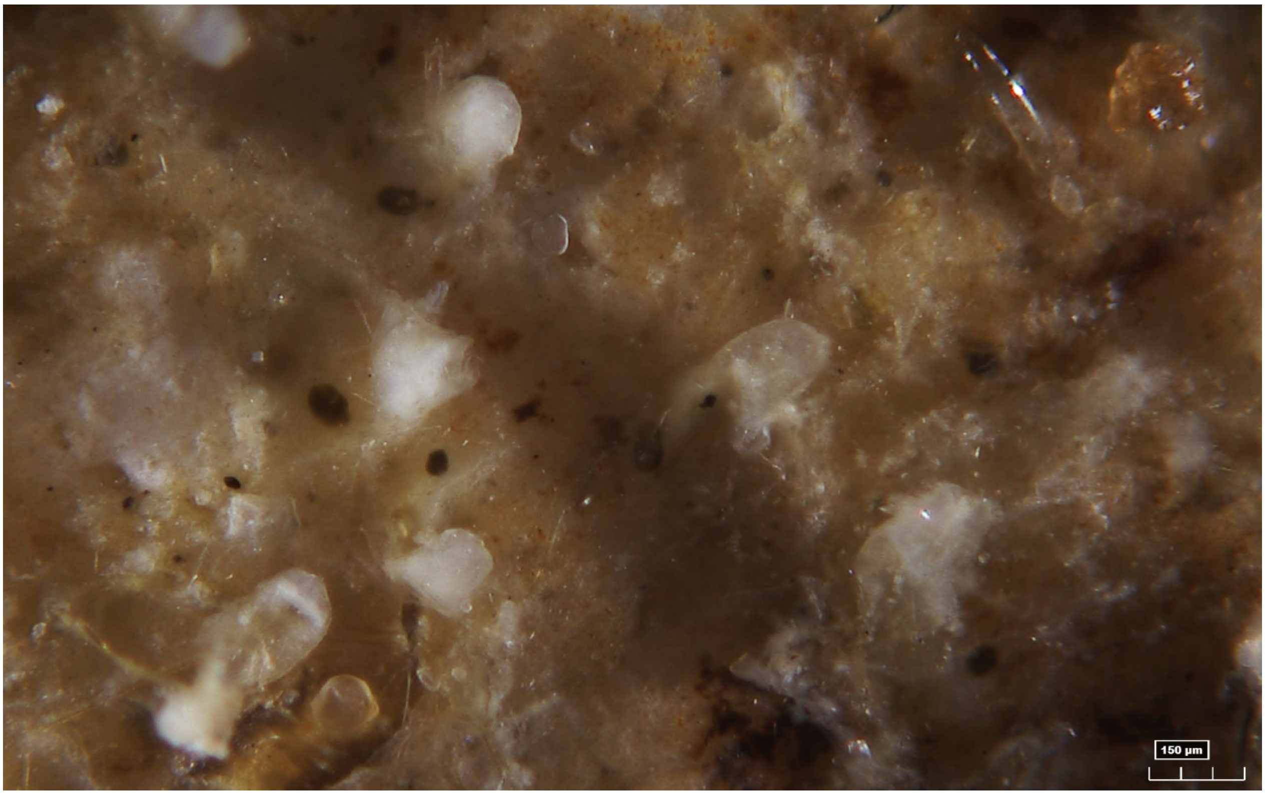

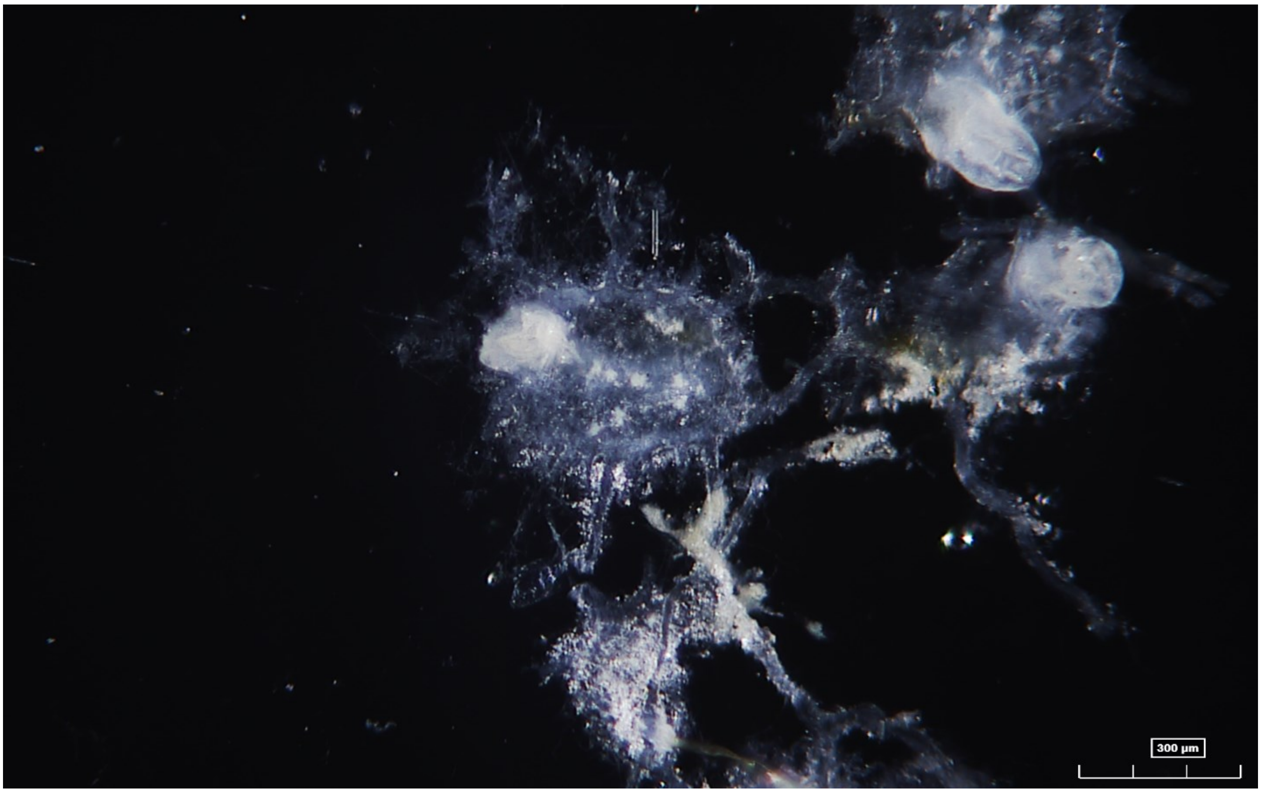

Penetrantia parva VIBE_Penet_NZ19_NL9A

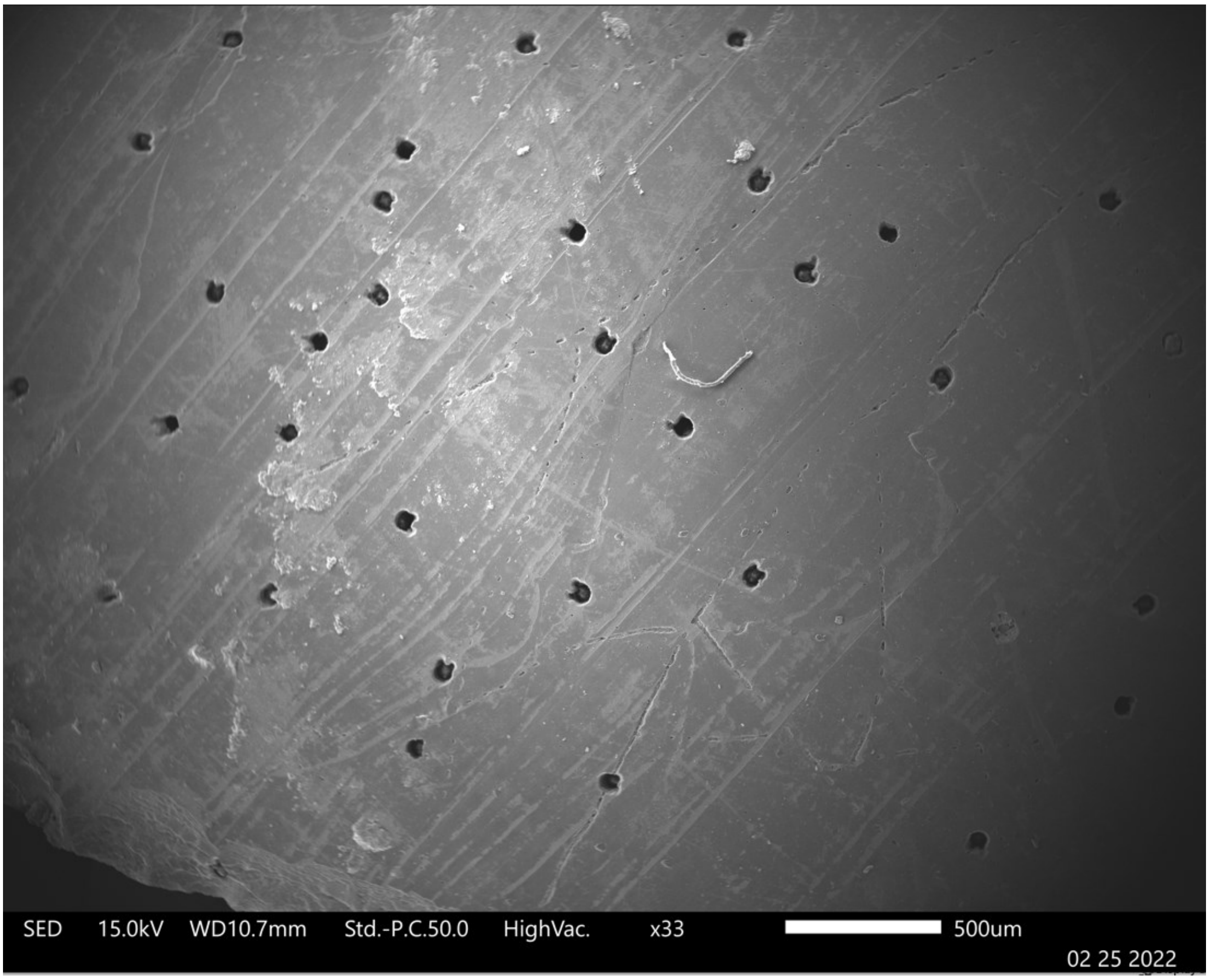

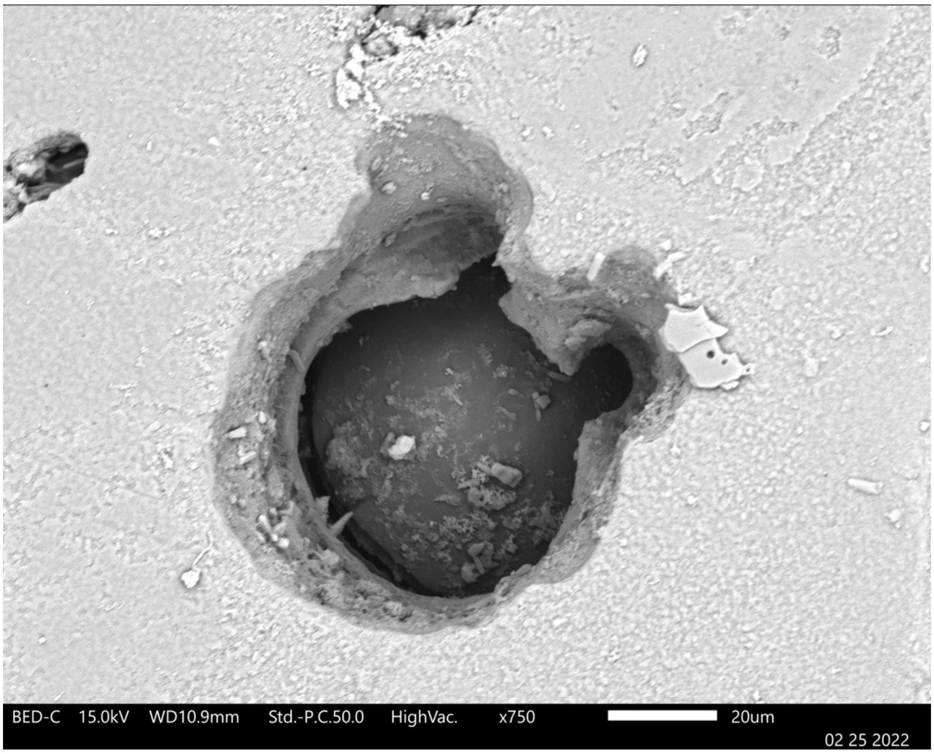

Penetrantia cf. *parva* VIBE_Penet_NZ21_PB3_PARVA

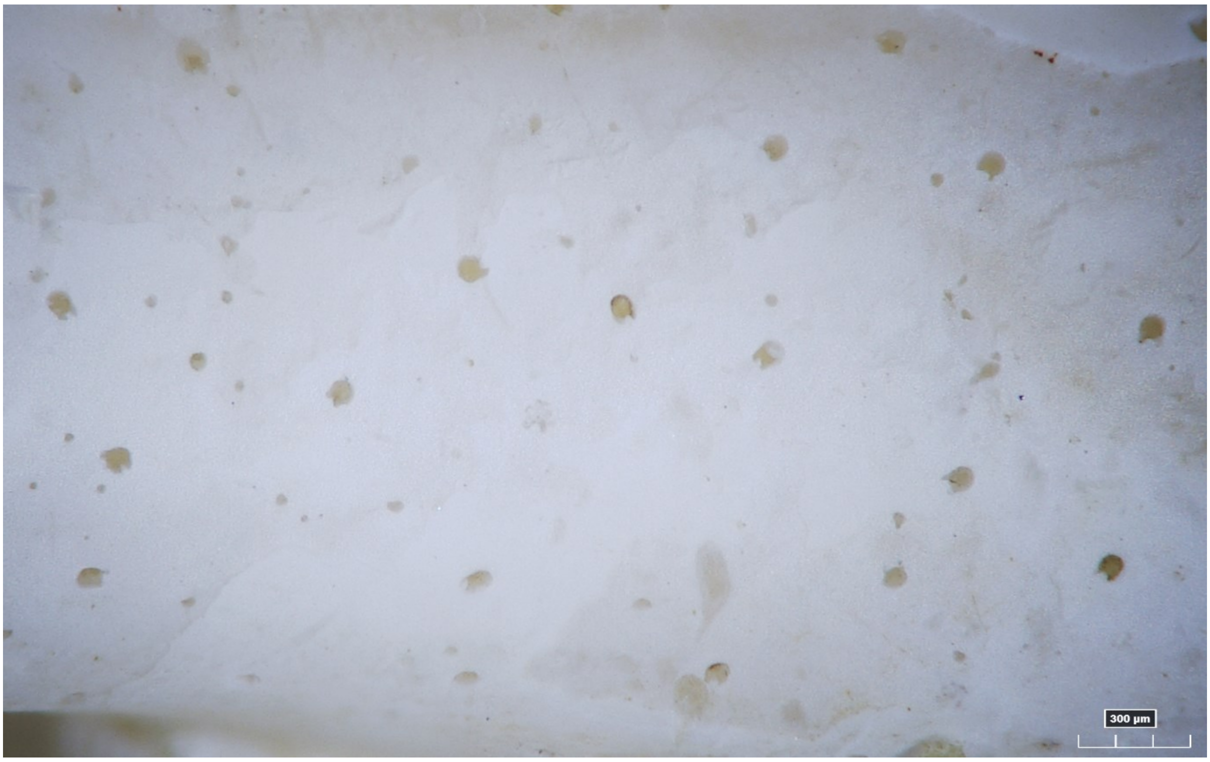

Penetrantia cf. *parva*   VIBE_Penet_CH22_25A

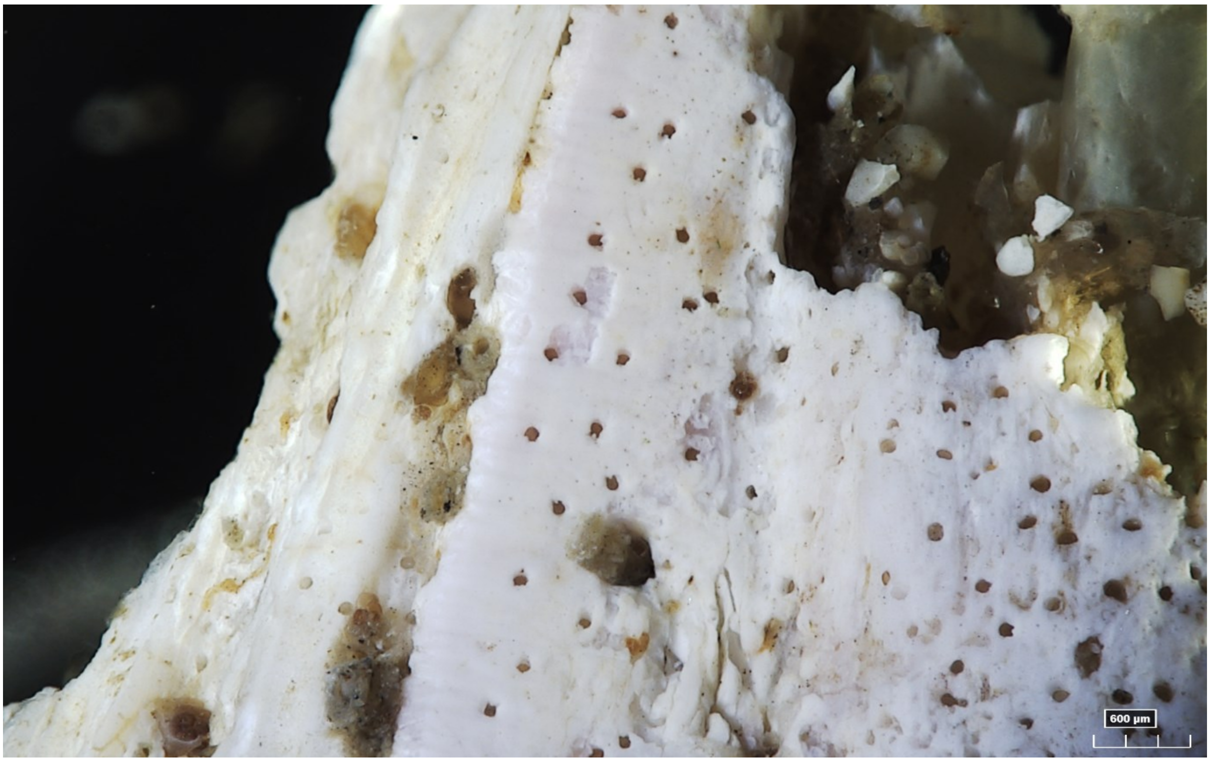

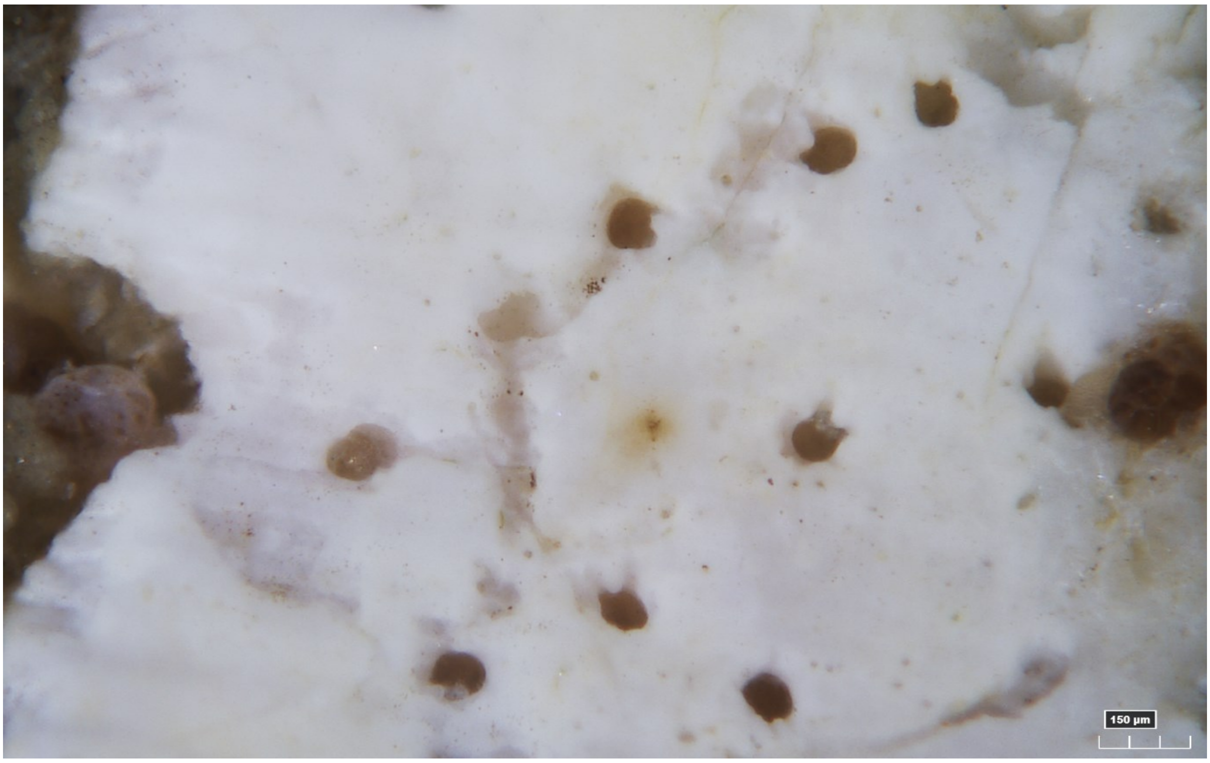

Penetrantia clionoides VIBE_Penet_Guam21_1A

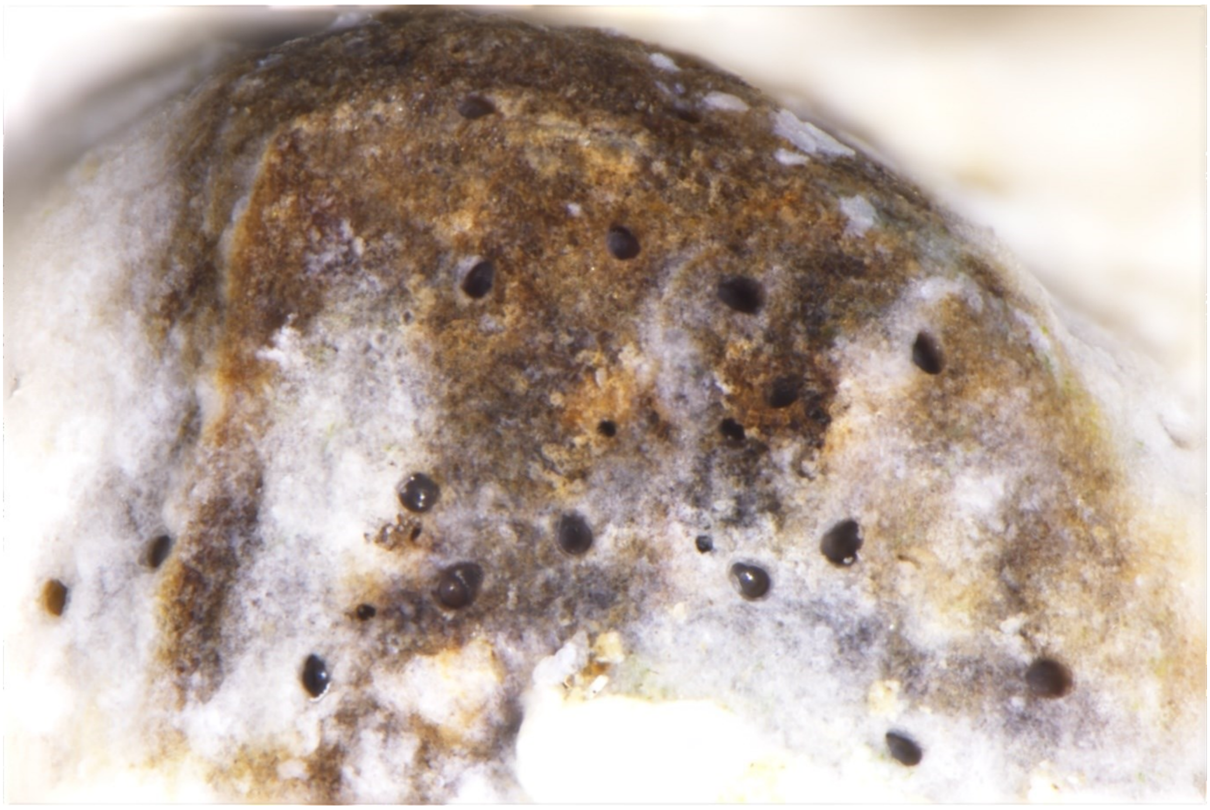

Penetrantia irregularis VIBE_ Penet_NZ21_PB3_IRREG

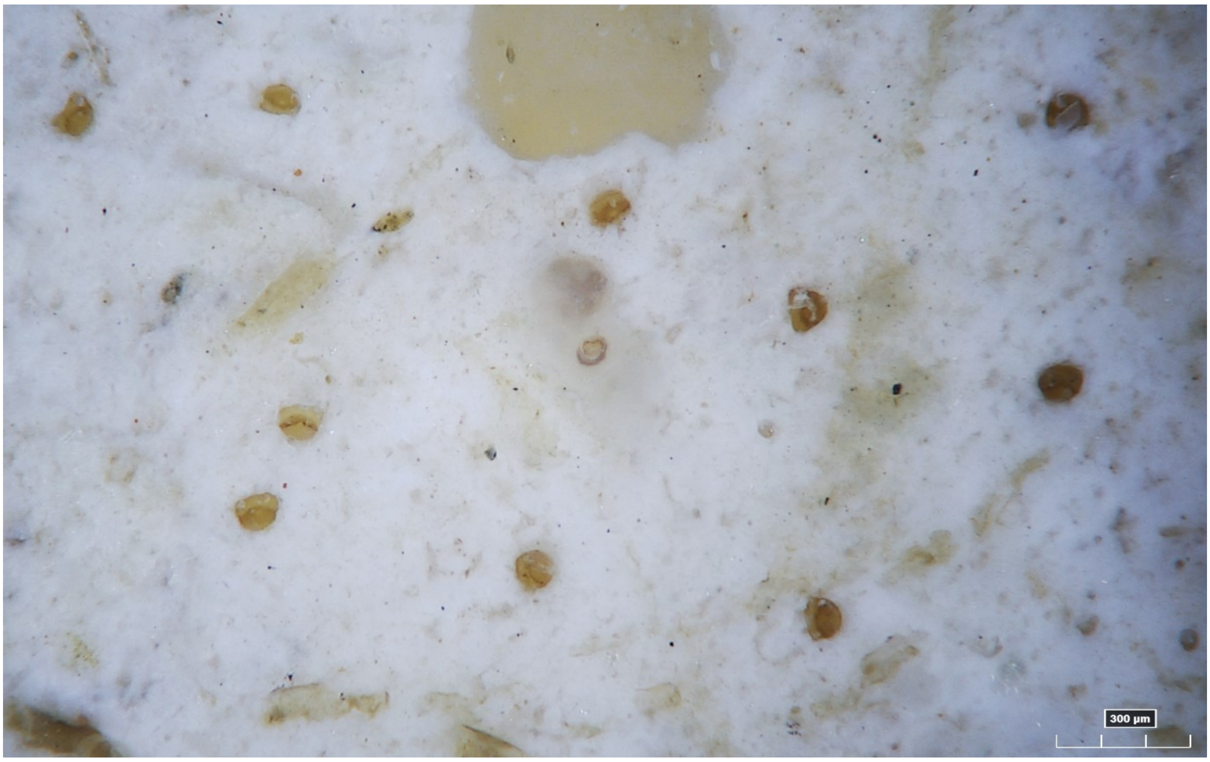

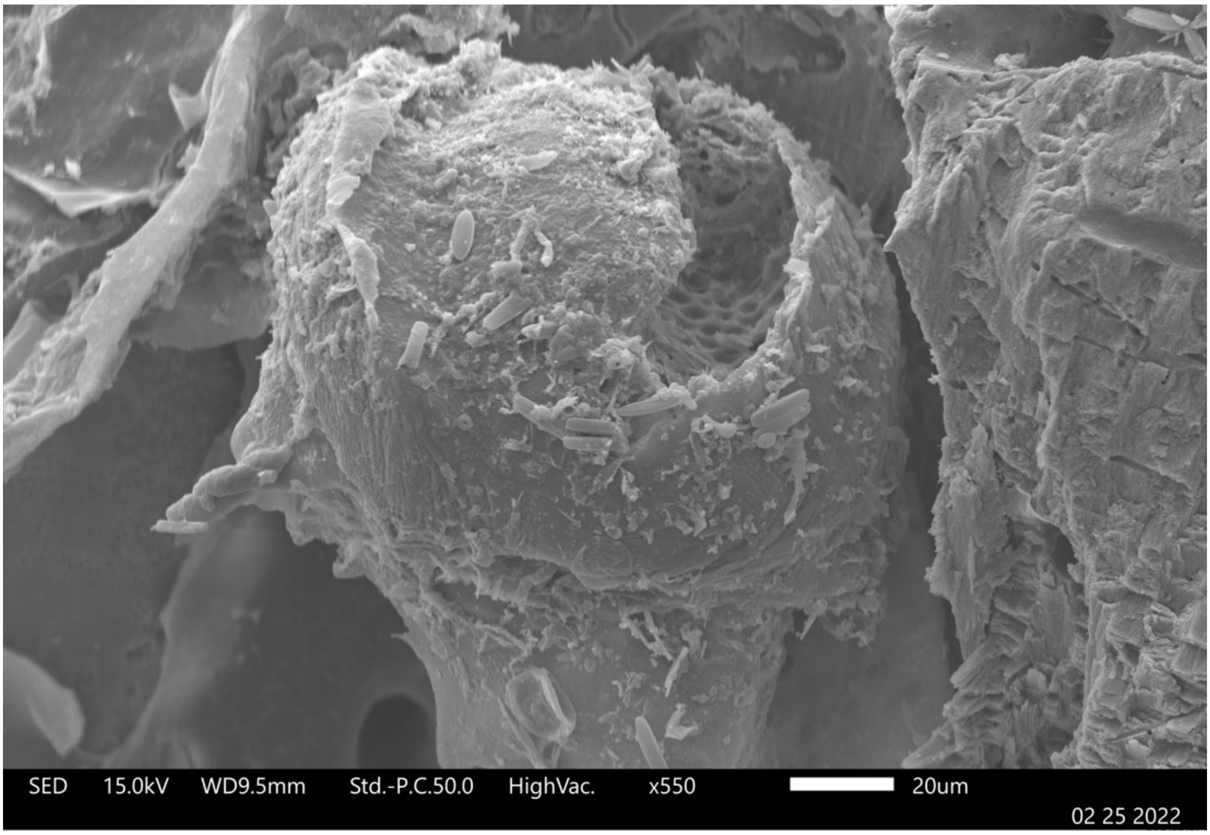

Penetrantia concharum VIBE_Penet_Sk19_39A

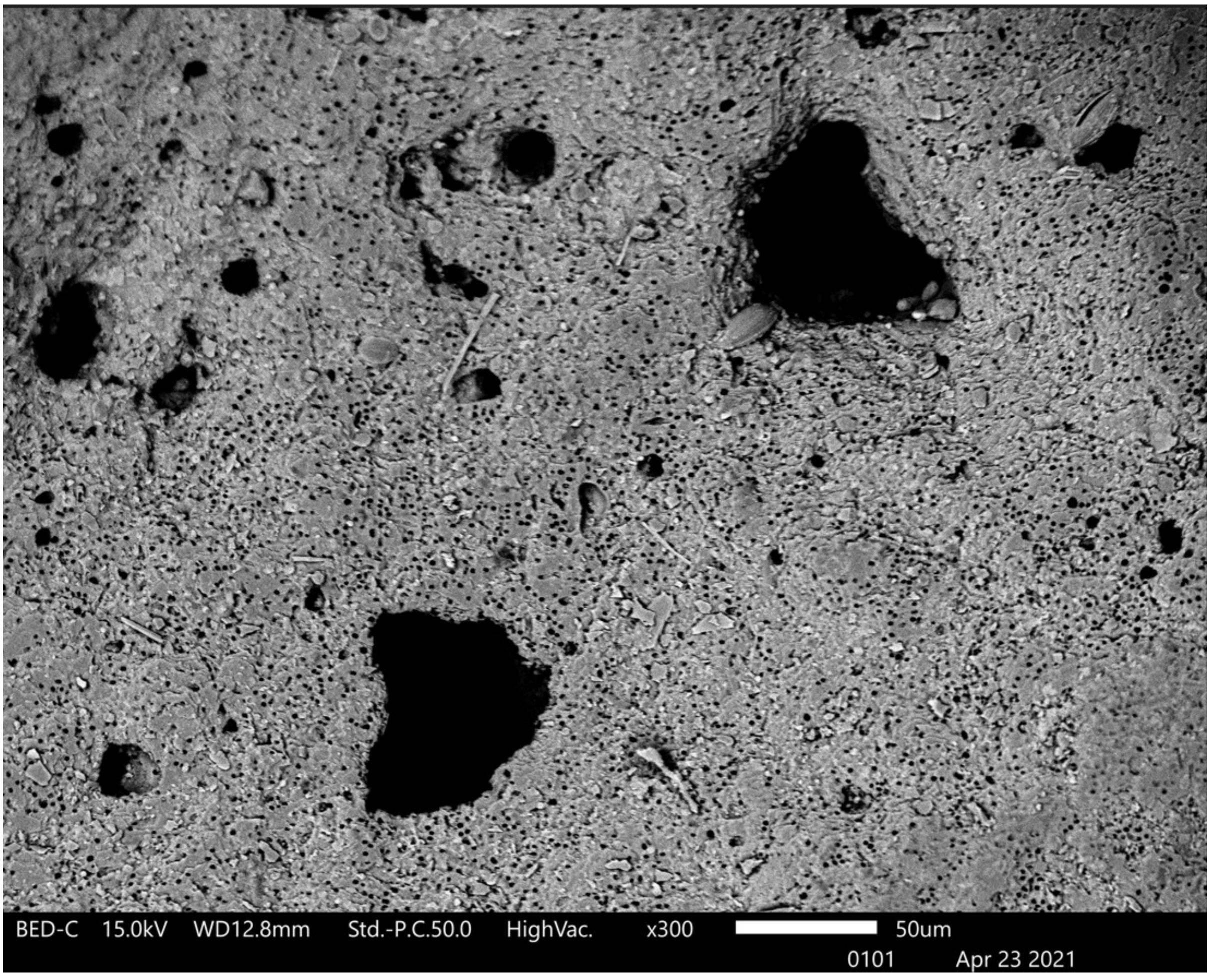

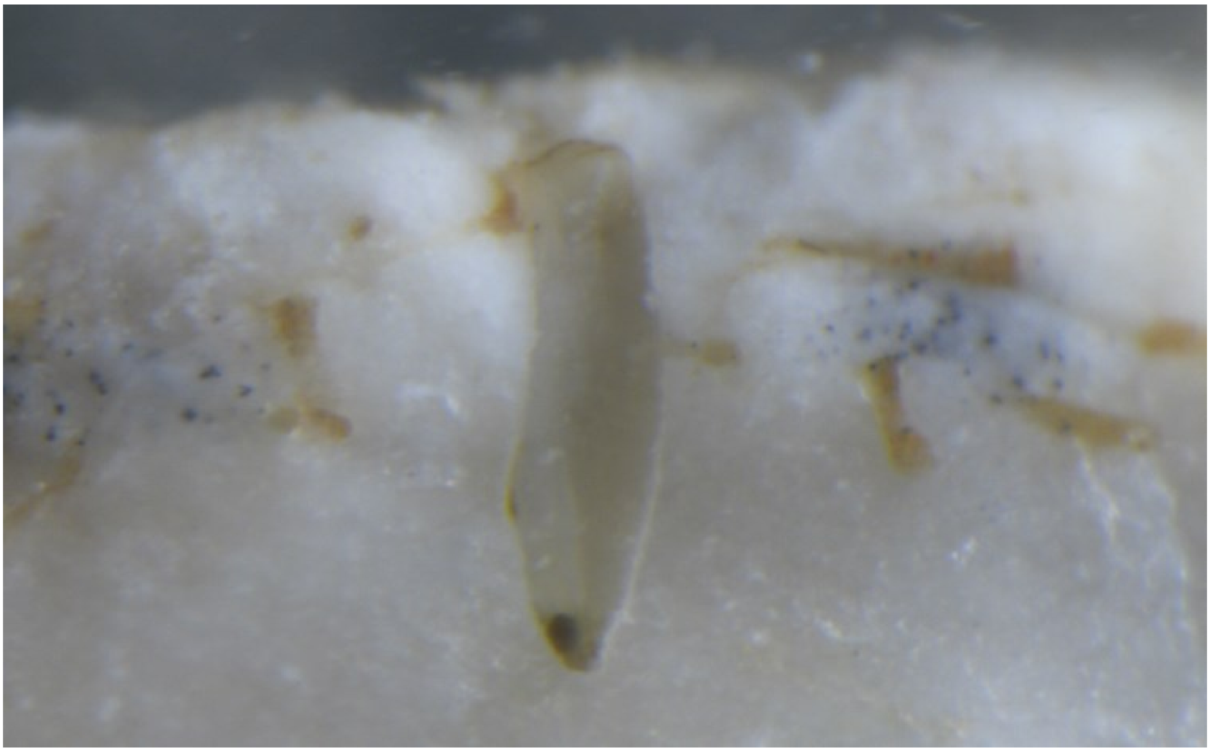

Penetrantia concharum VIBE_ Penet_FR20_6A

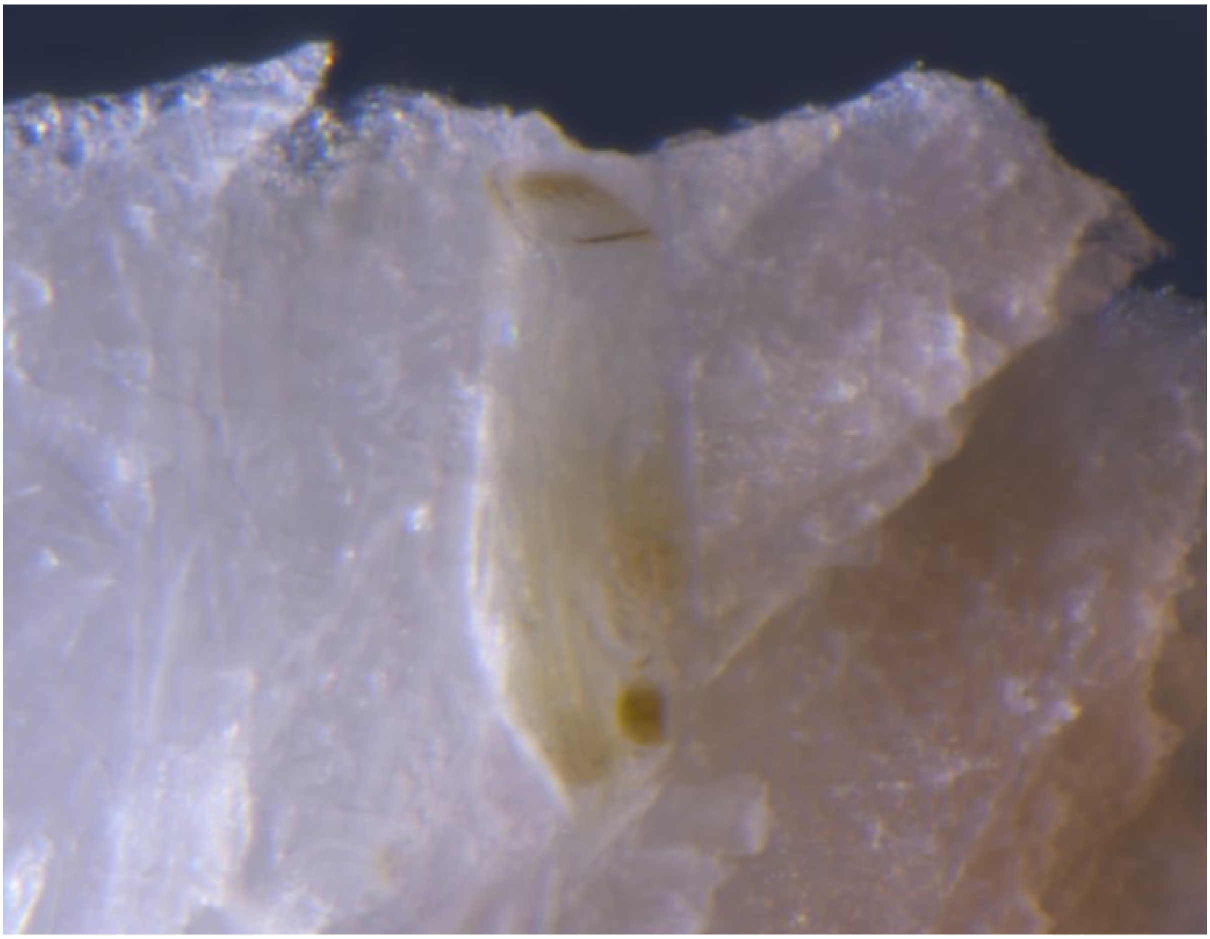

Penetrantia sp.   VIBE_ Penet_FR21_35A

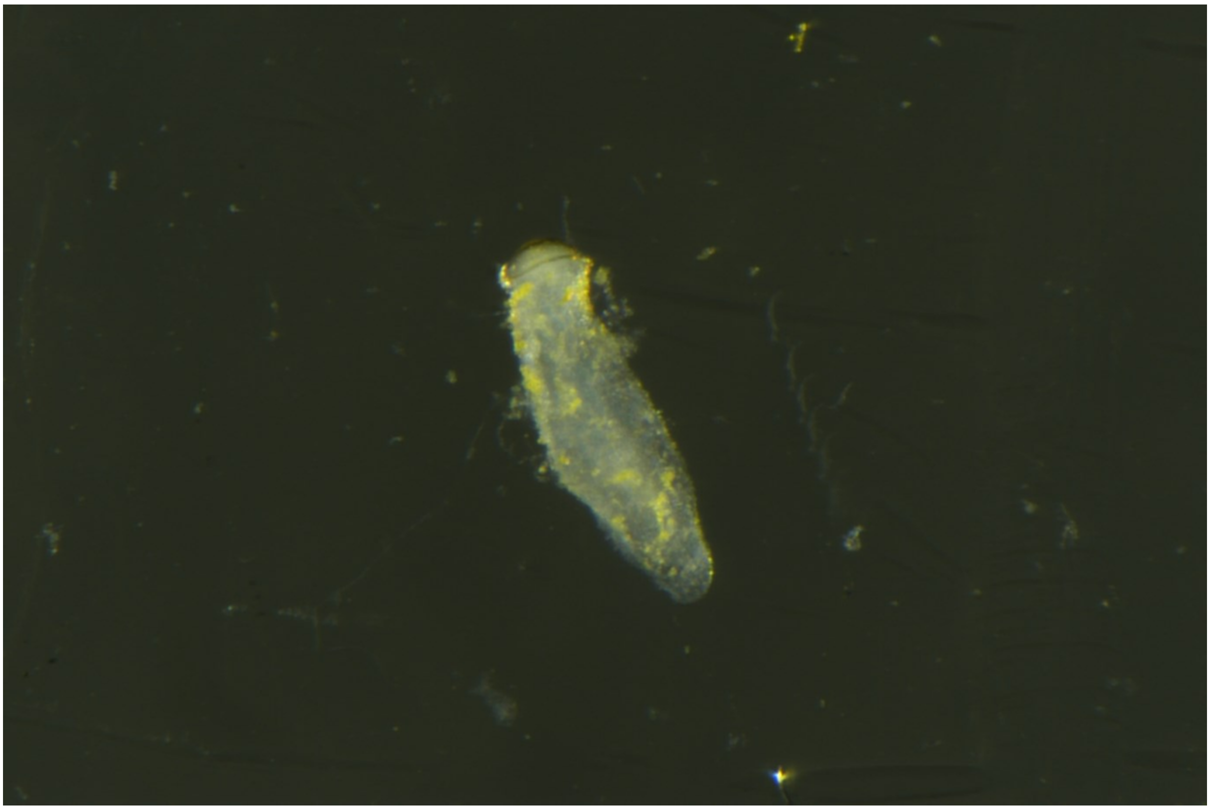

Penetrantia japonica sp. nov.   VIBE_ Penet_JT20_10A

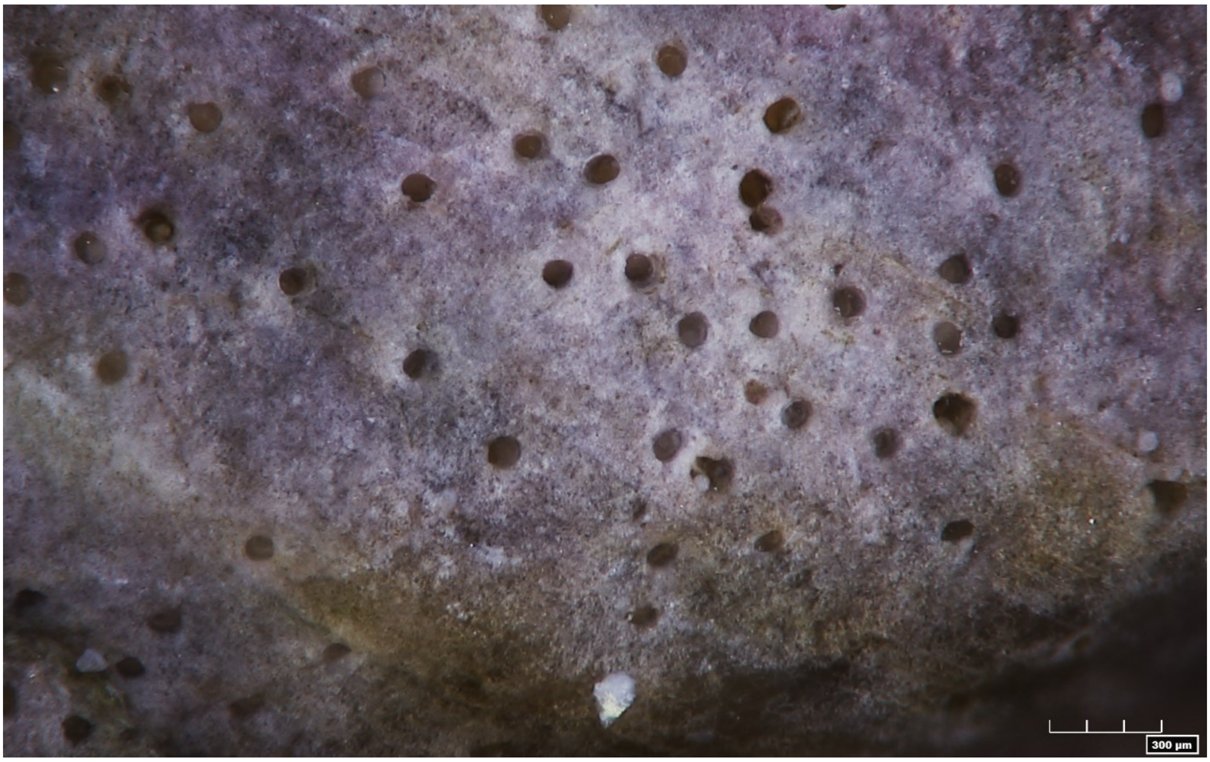

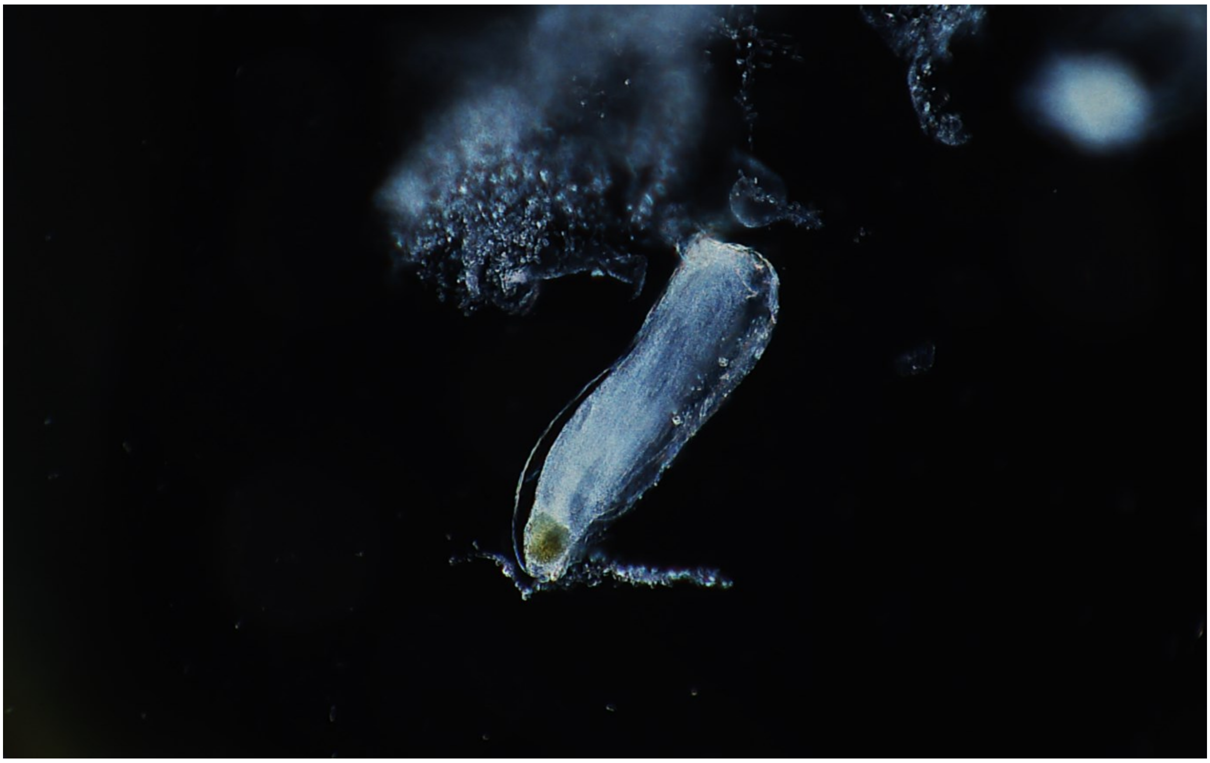

Alcyonidium polyoum VIBE_ Alcy-FR22-62A

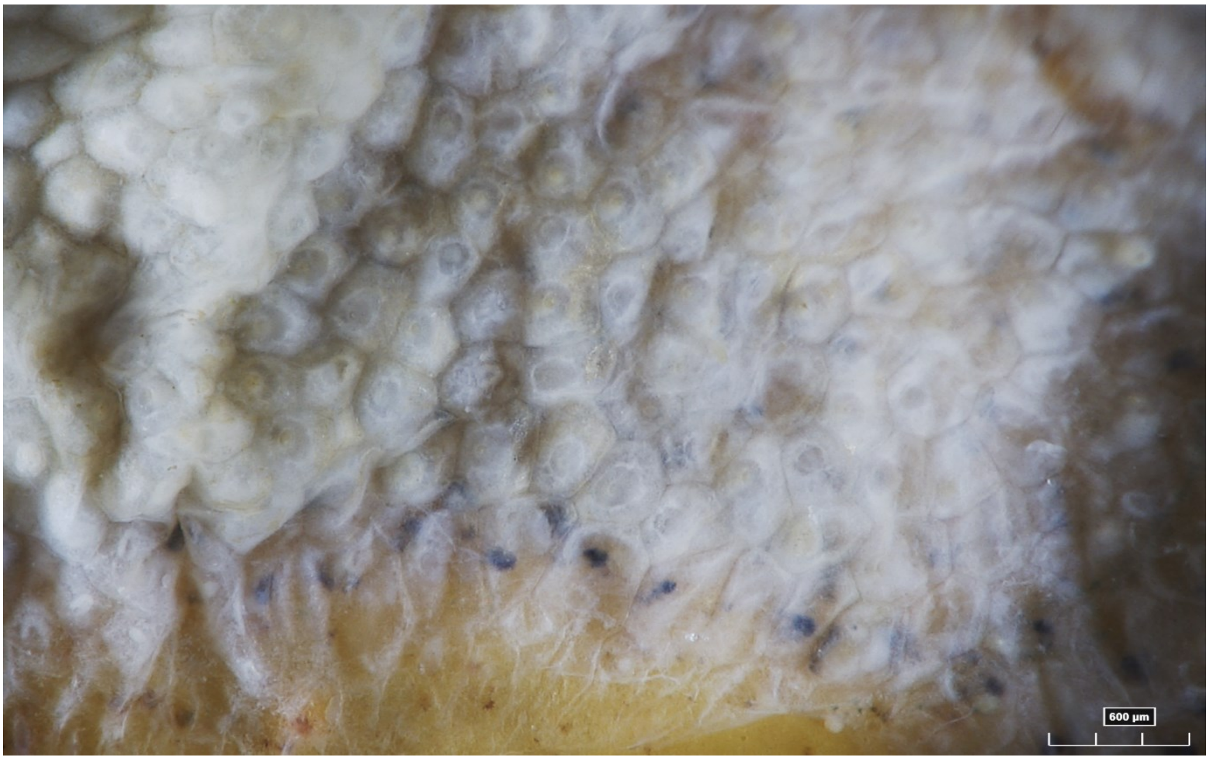

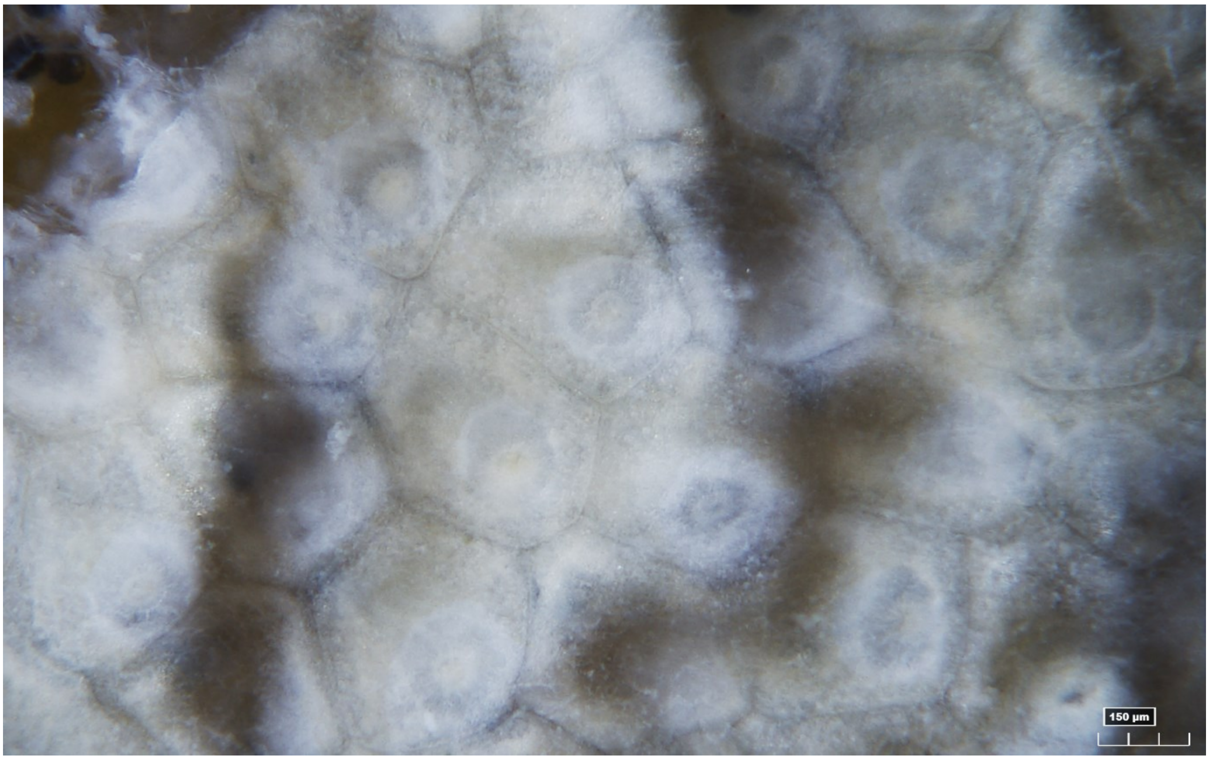

Alcyonidium gelatinosum VIBE_Alcym_FR22_57A

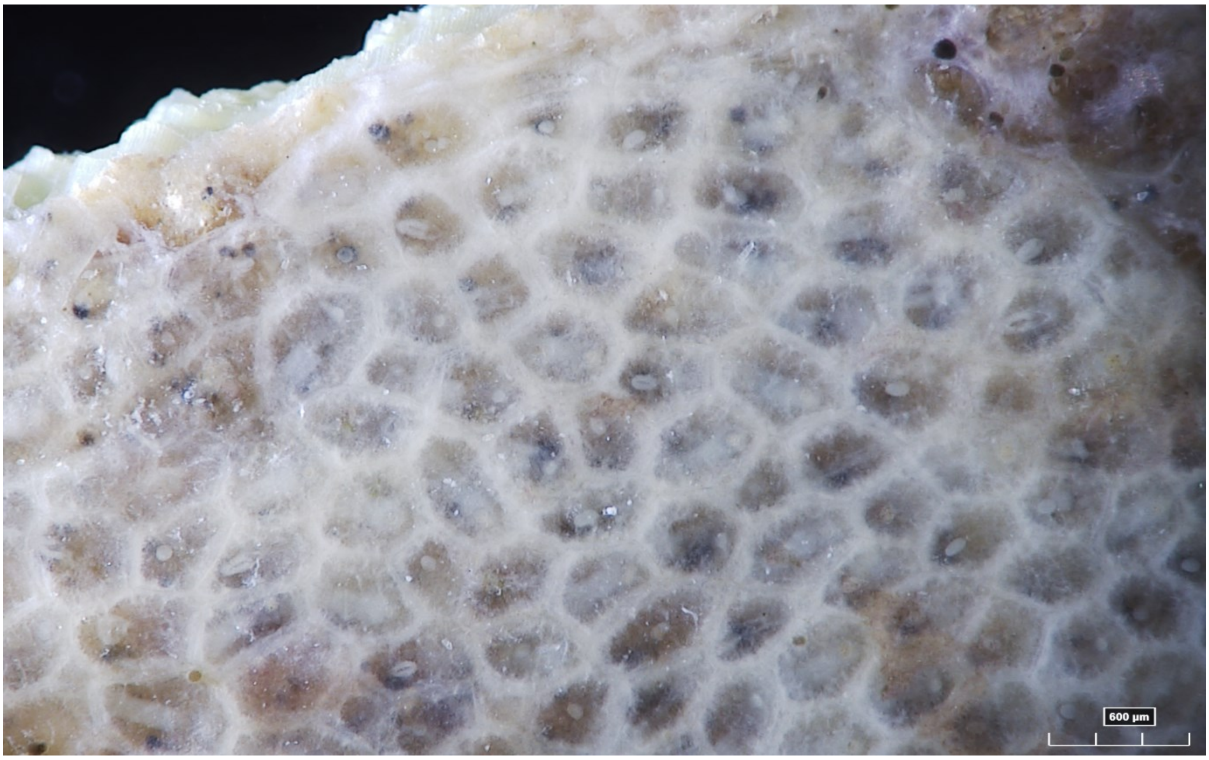

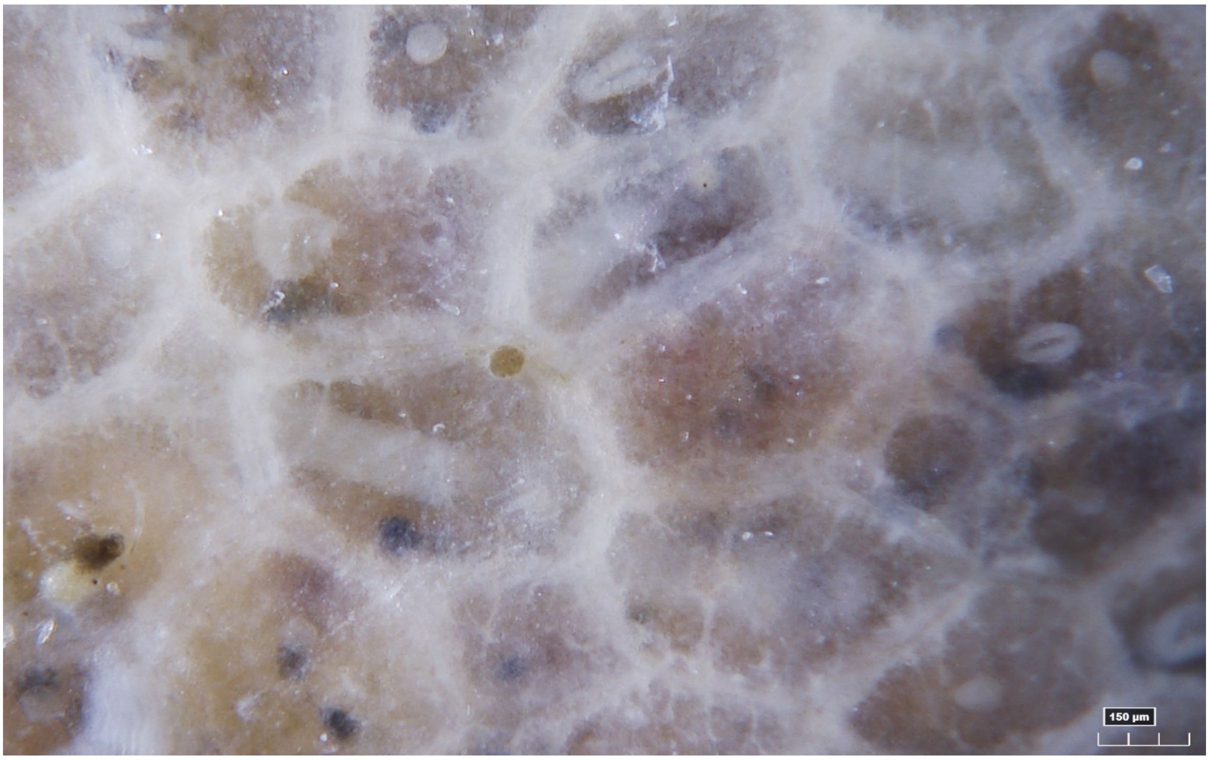

Pherusella liowae VIBE_ Pher_SP19_2A

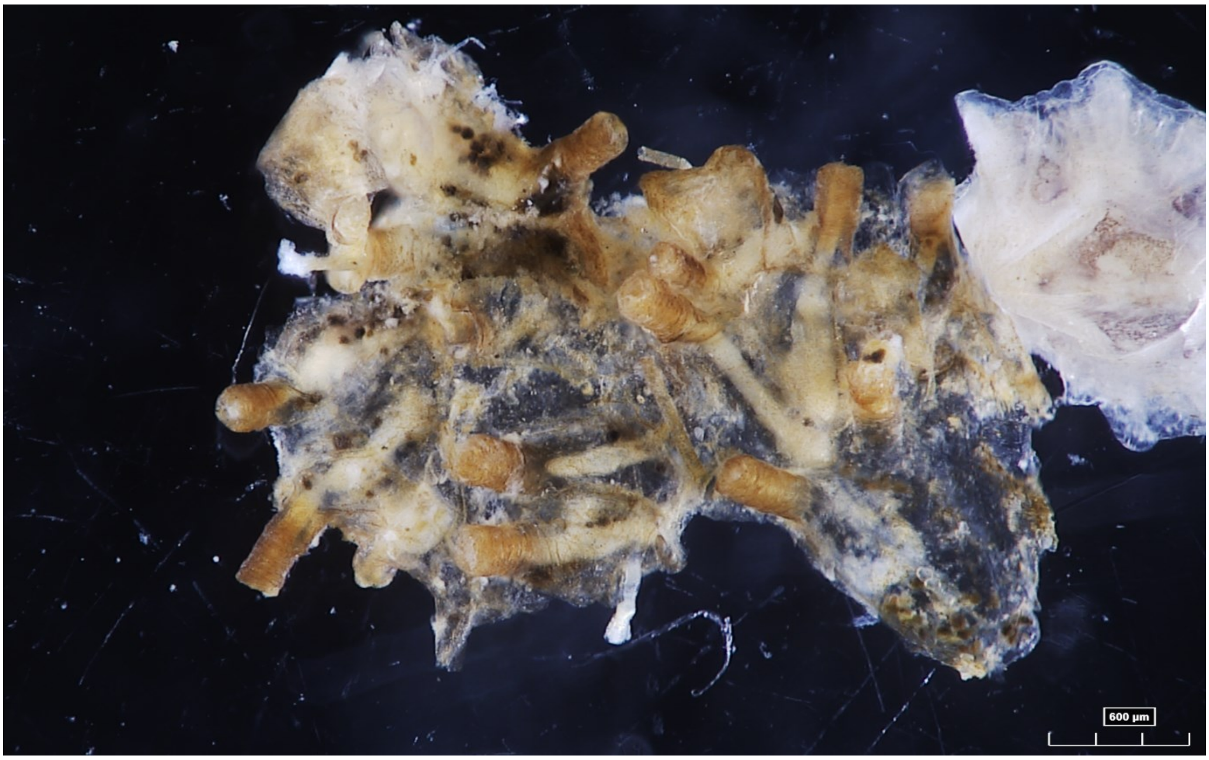

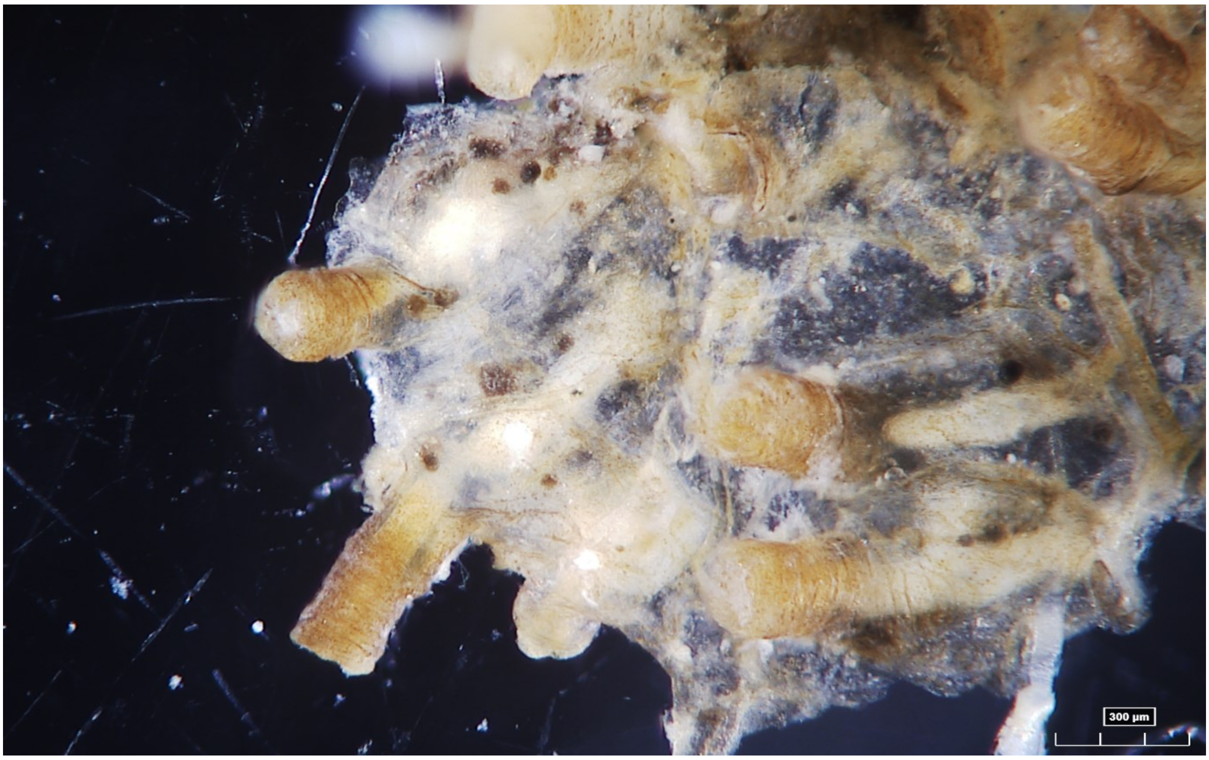

Flustrellidra hispida VIBE_Flus_FR22_61A

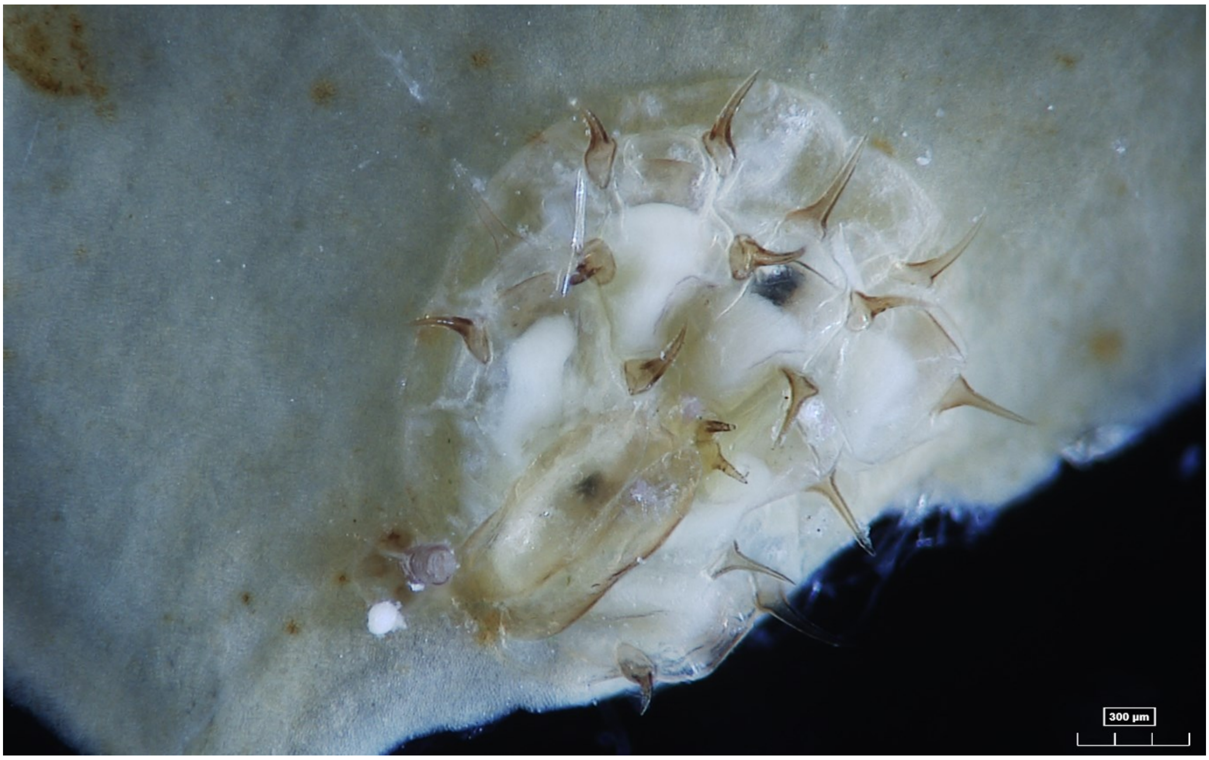

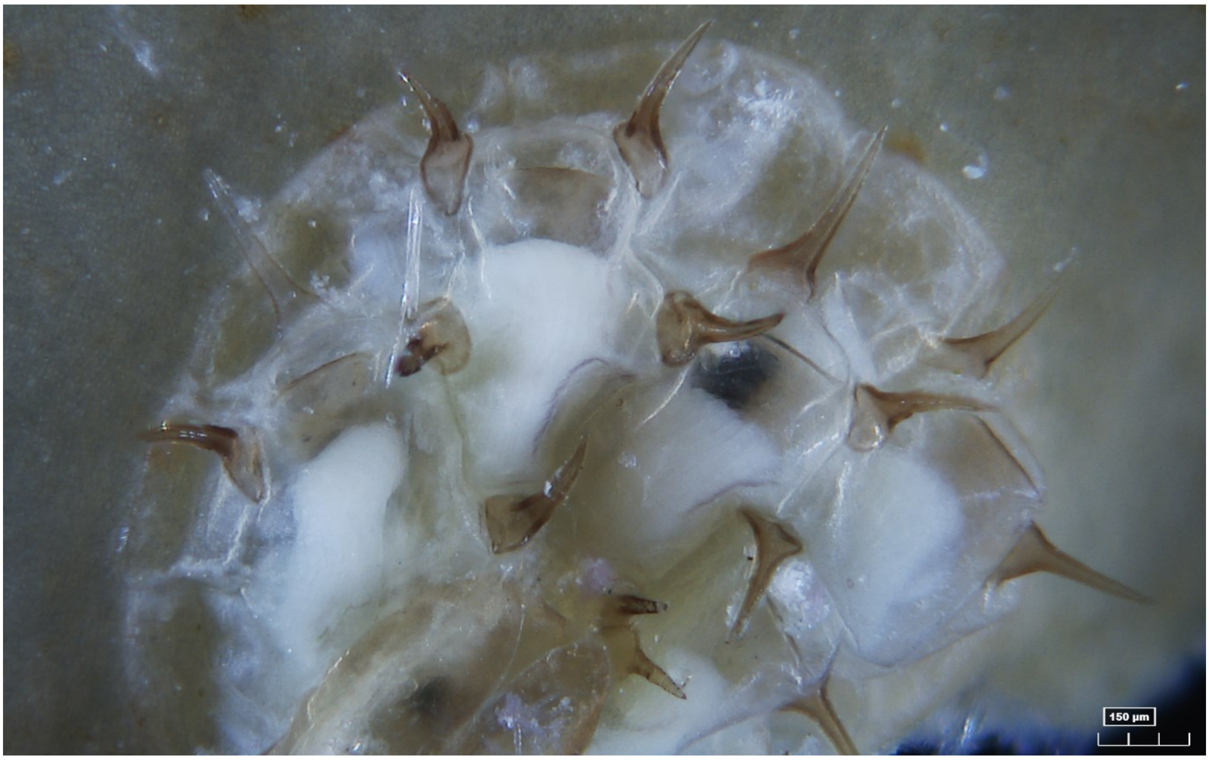

Sundanella sibogae VIBE_Sund-SP19-1A

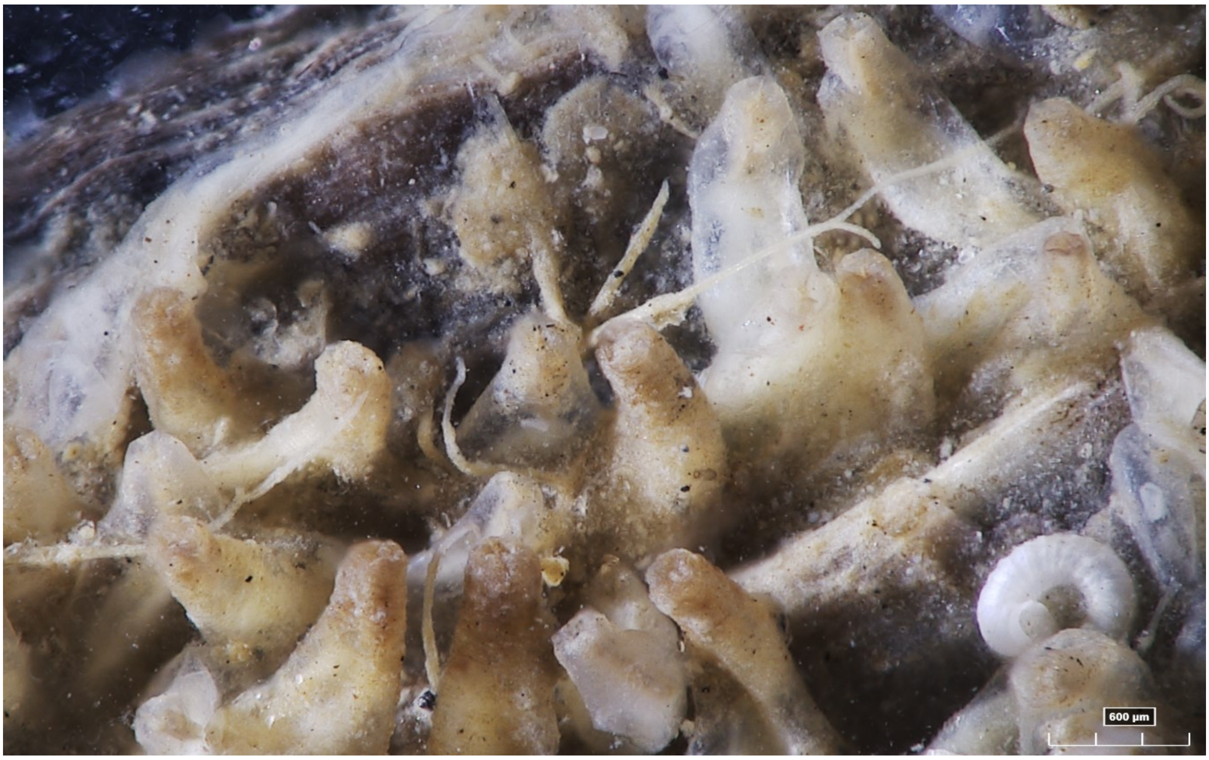

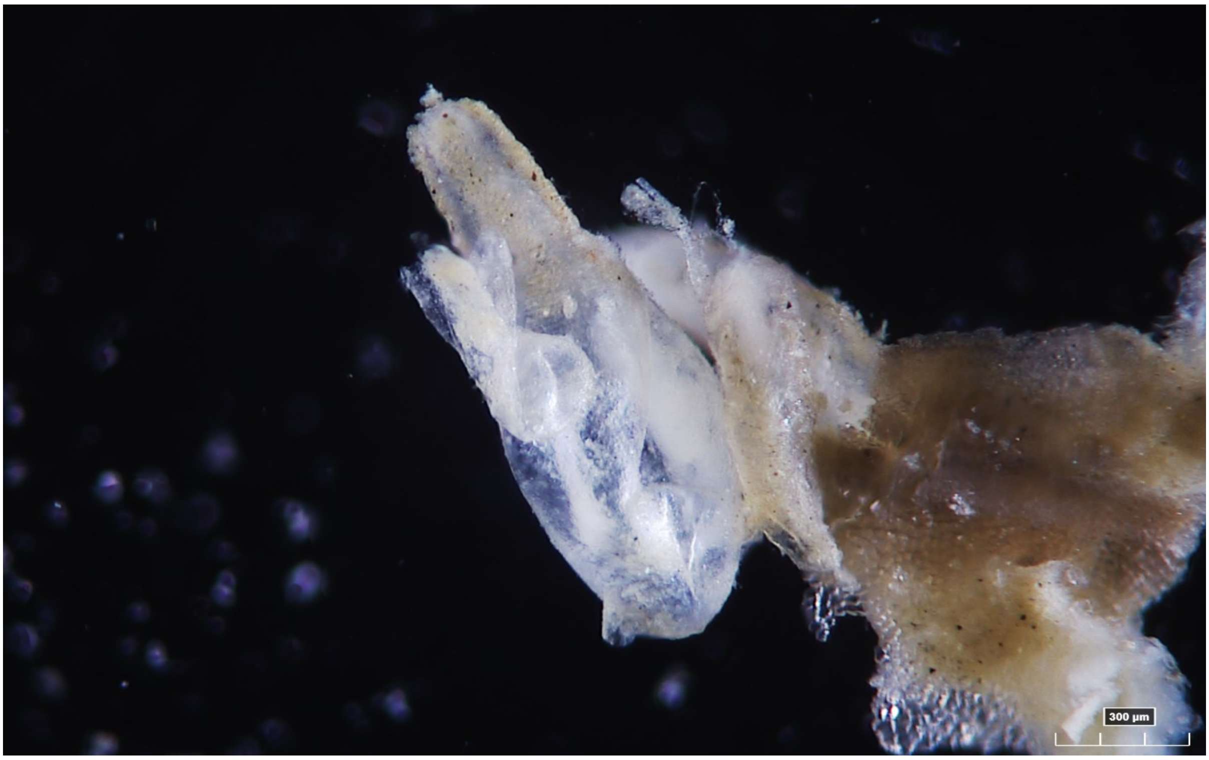

Sundanella sibogae VIBE_ Sund-Brazil22-3

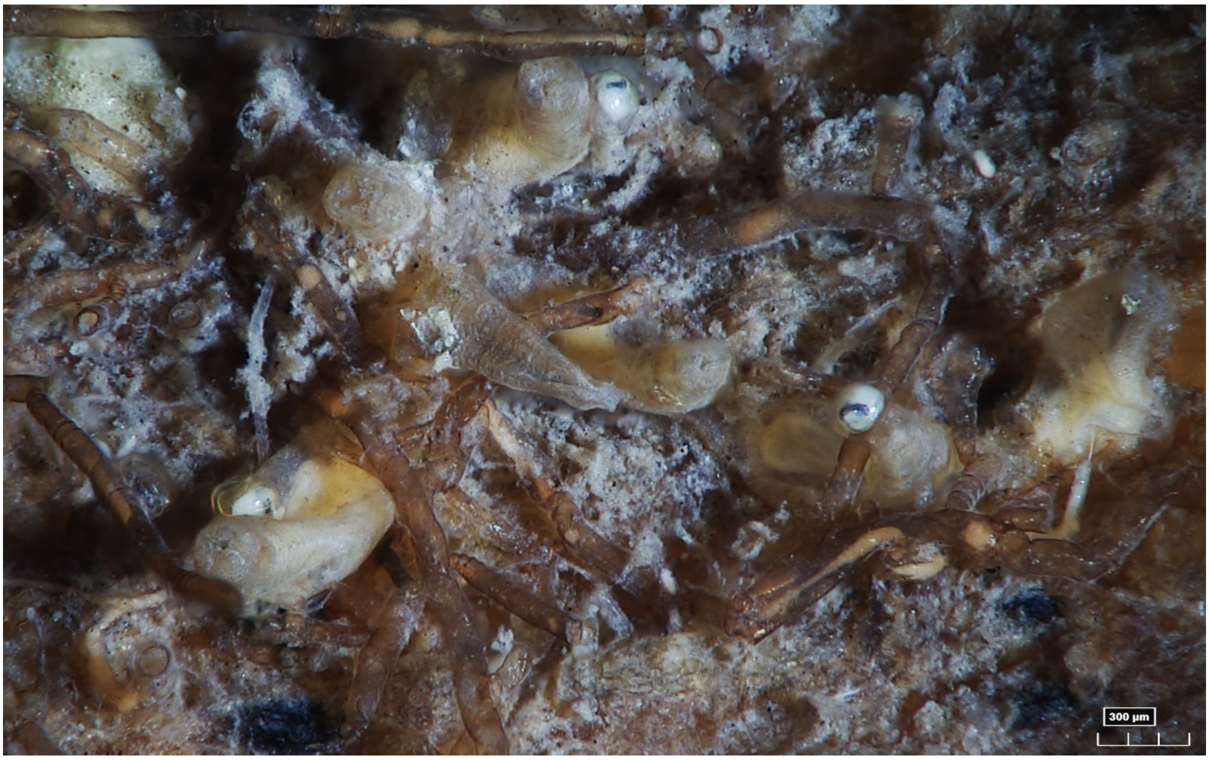

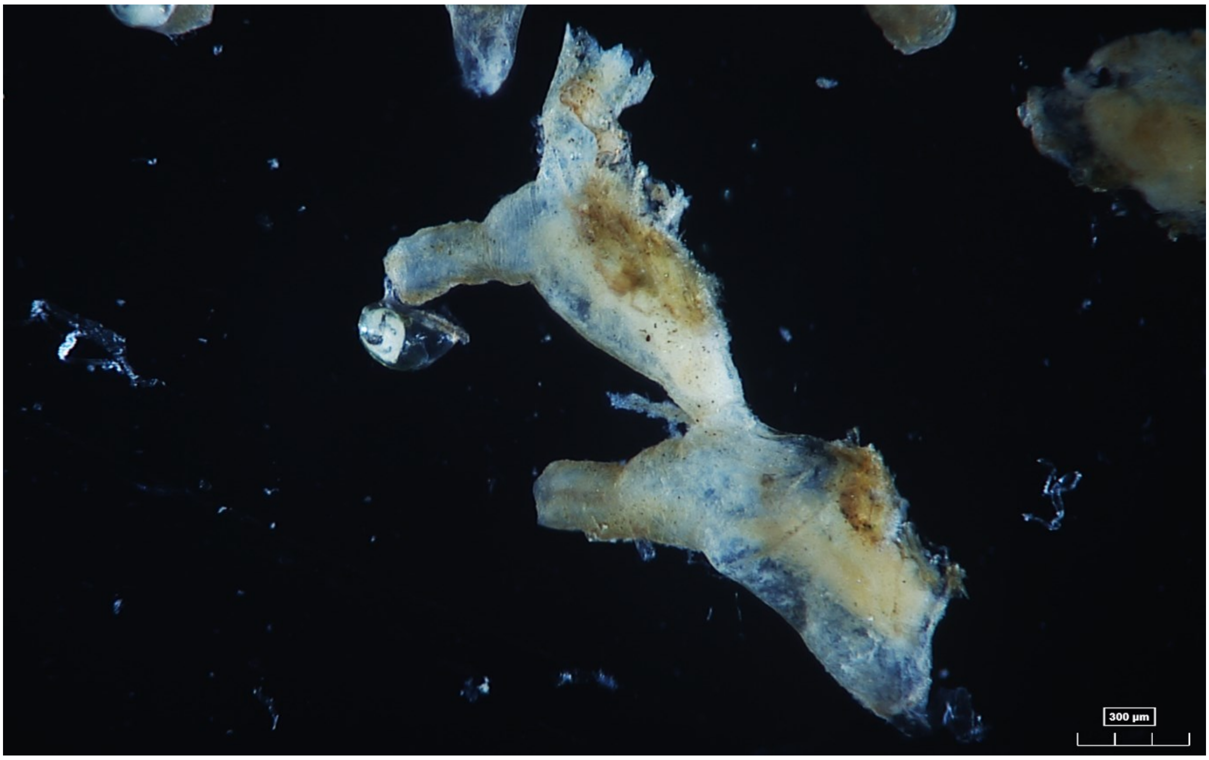

Aeverrillia setigera VIBE_ Aever-Brazil22-2

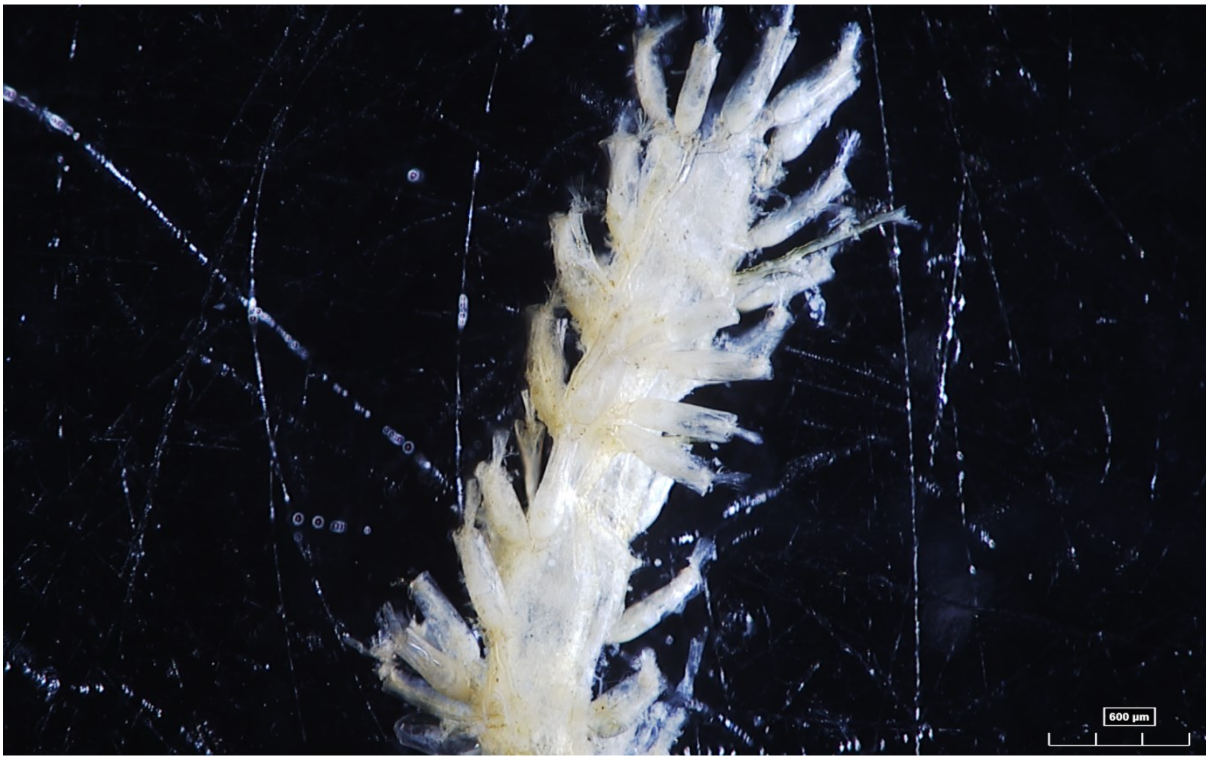

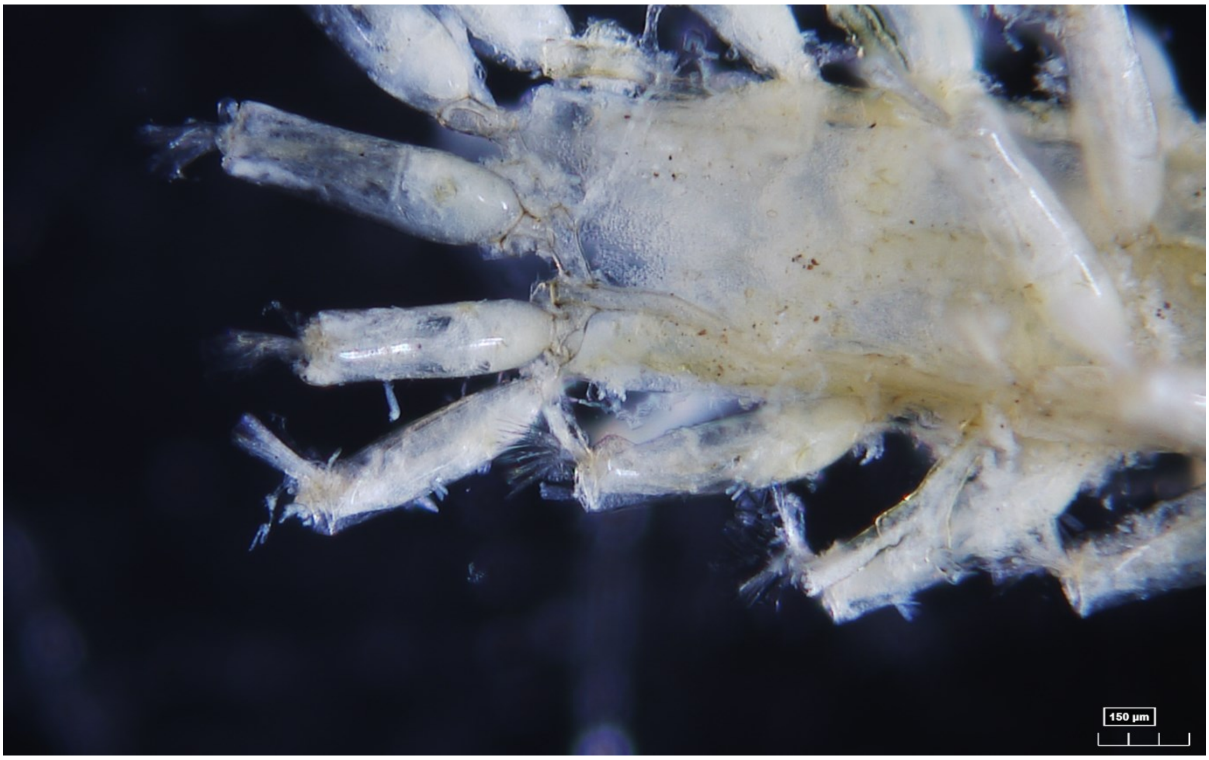

Hislopia malayensis VIBE_ His-Thai20-8A

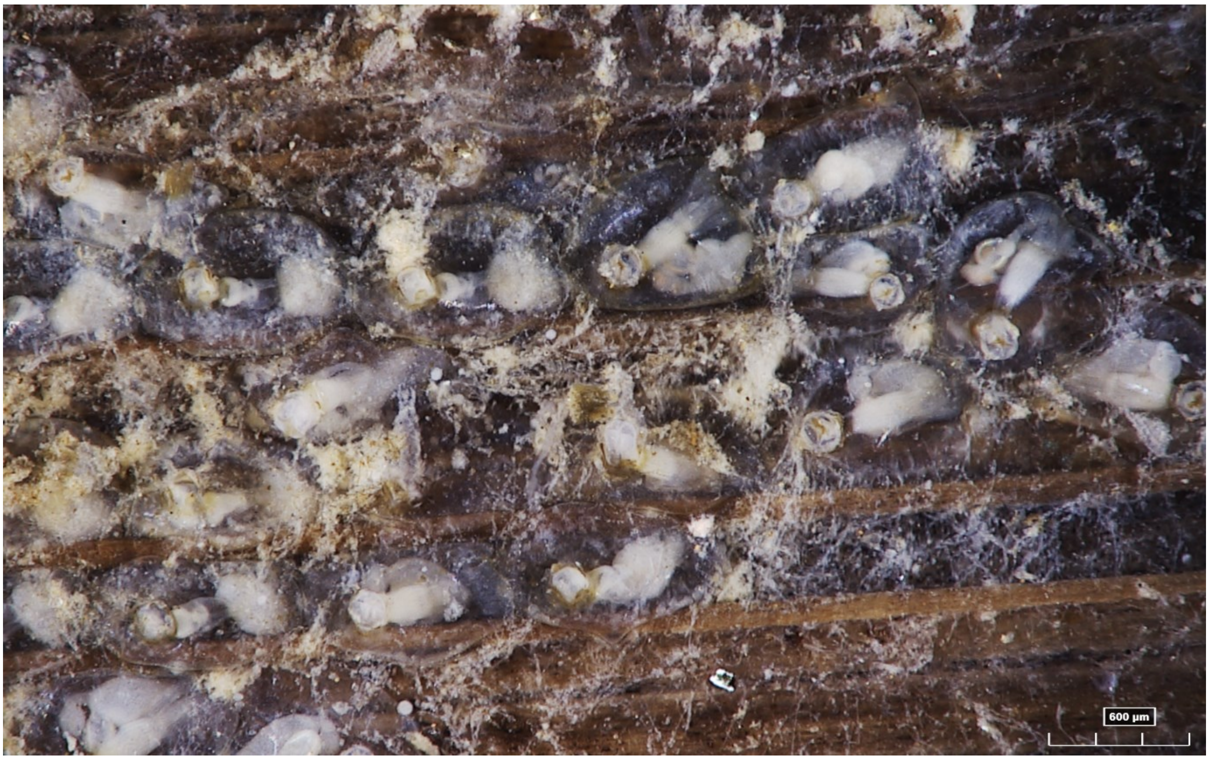

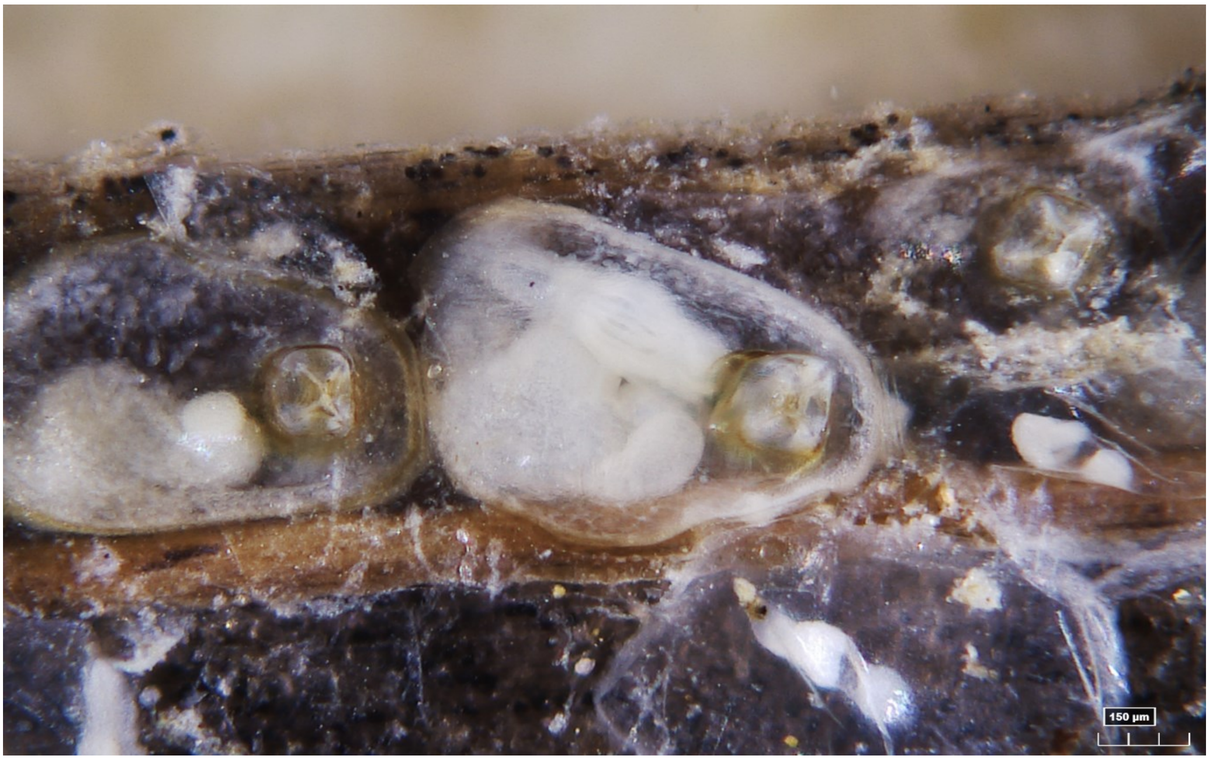

Amphibiobeania epiphylla VIBE_Amphi-NIWA04-1

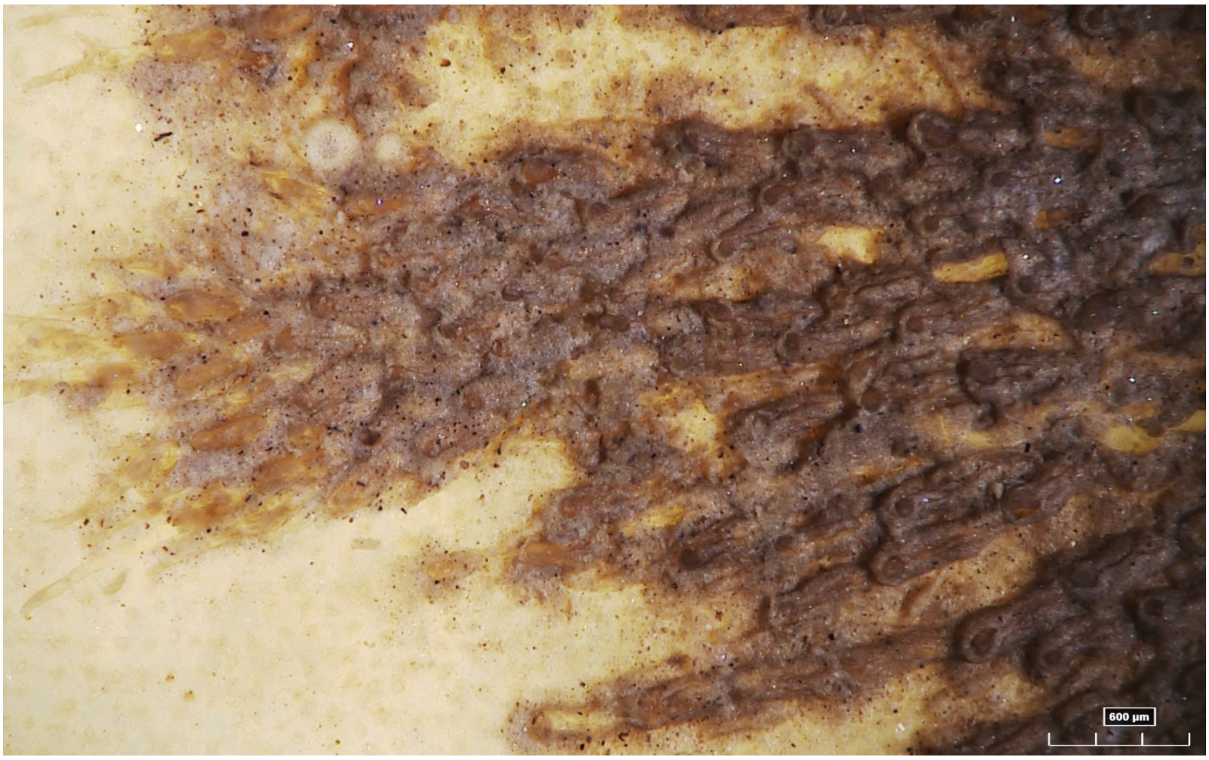

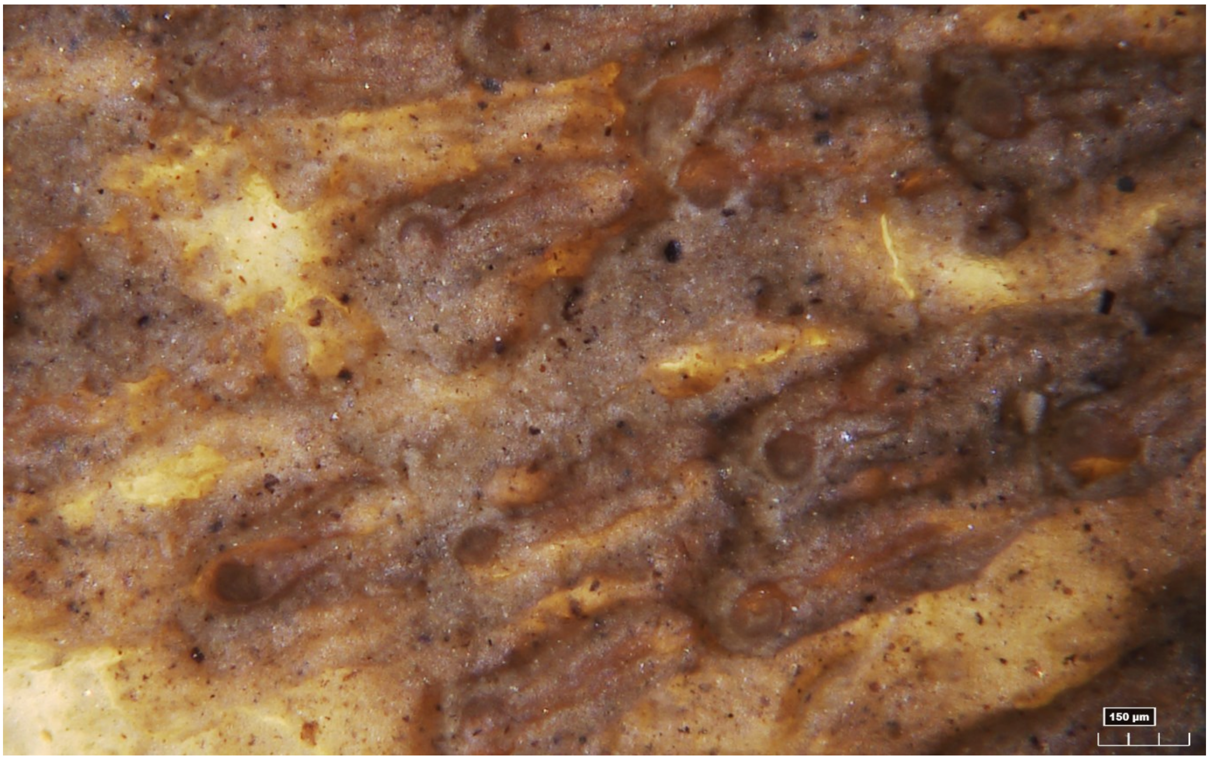

Terebripora sp. VIBE_ Tere-CH22-4A

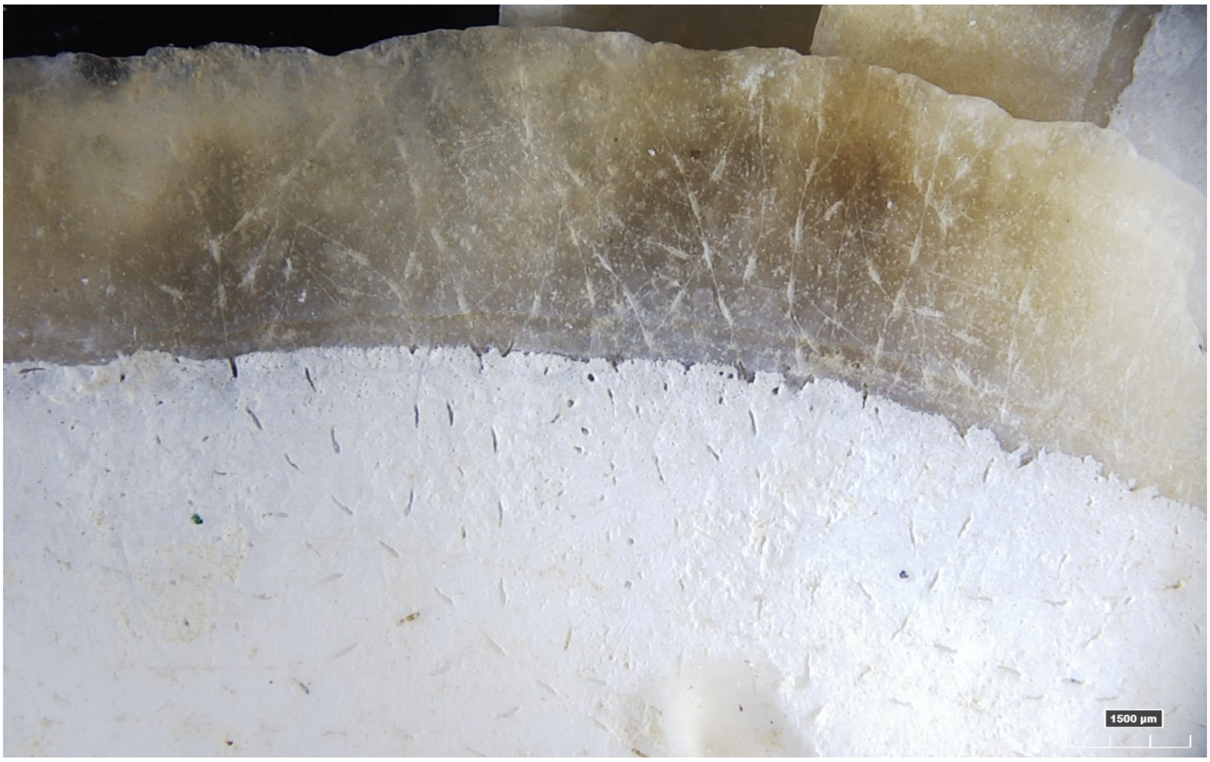

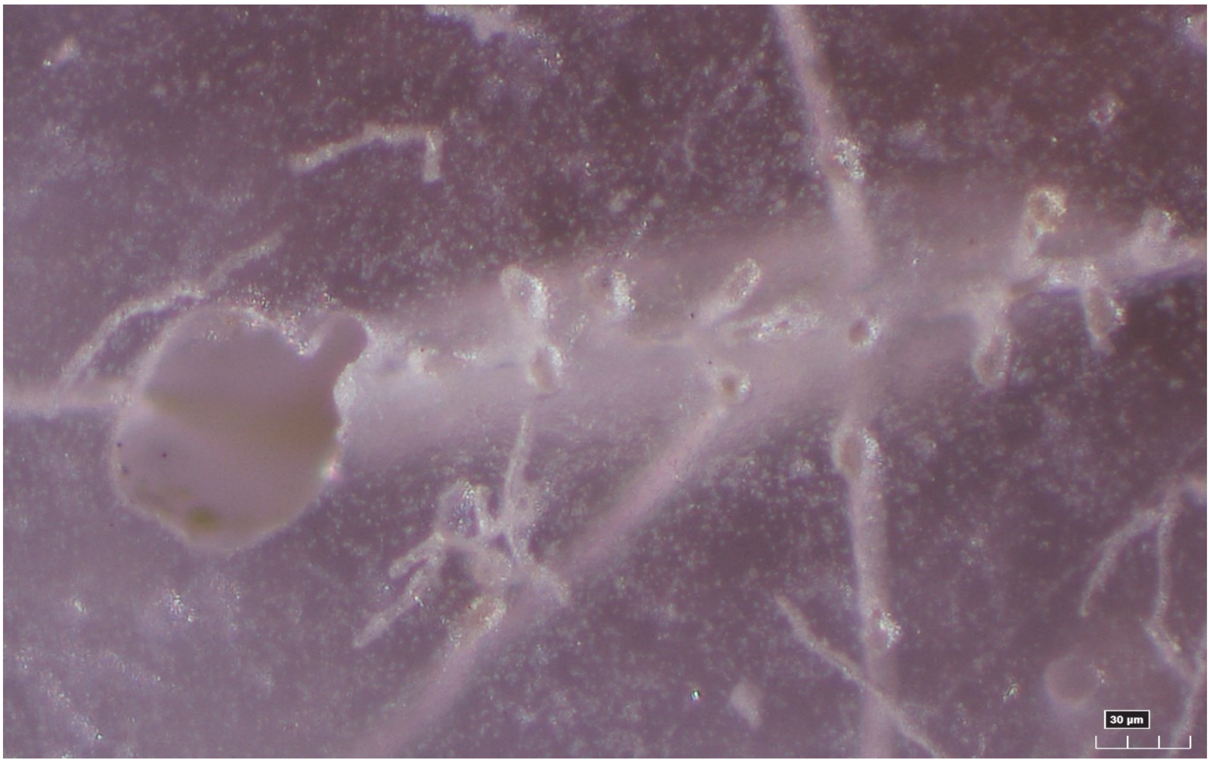

Amathia distans VIBE_ Amathd-Brazil22-4

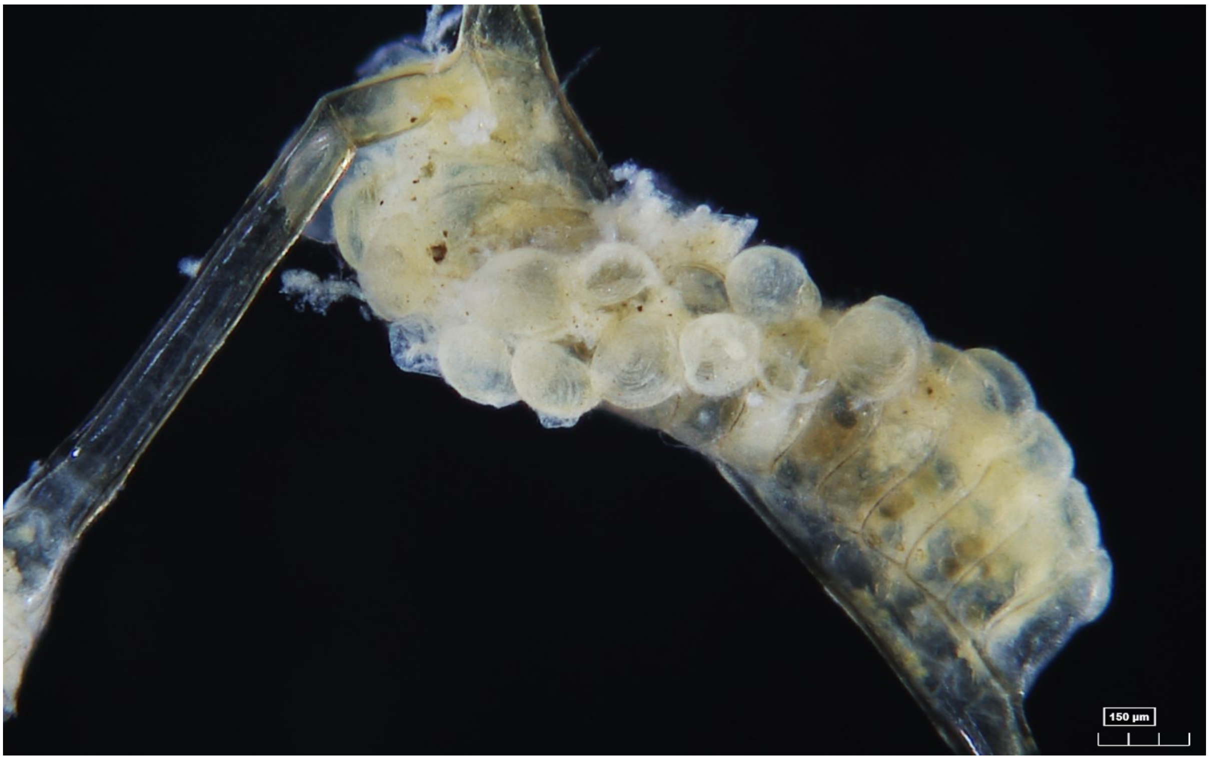

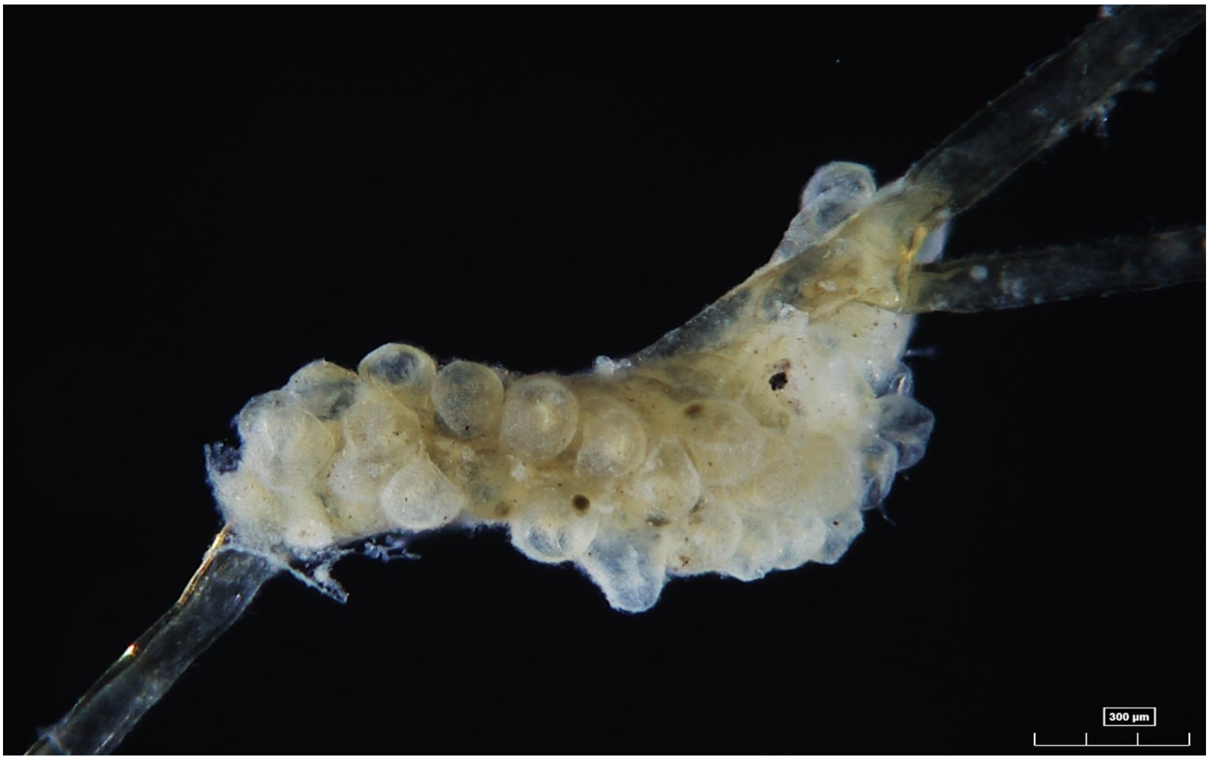

Amathia ernsti VIBE_ Amath-Brazil22-1

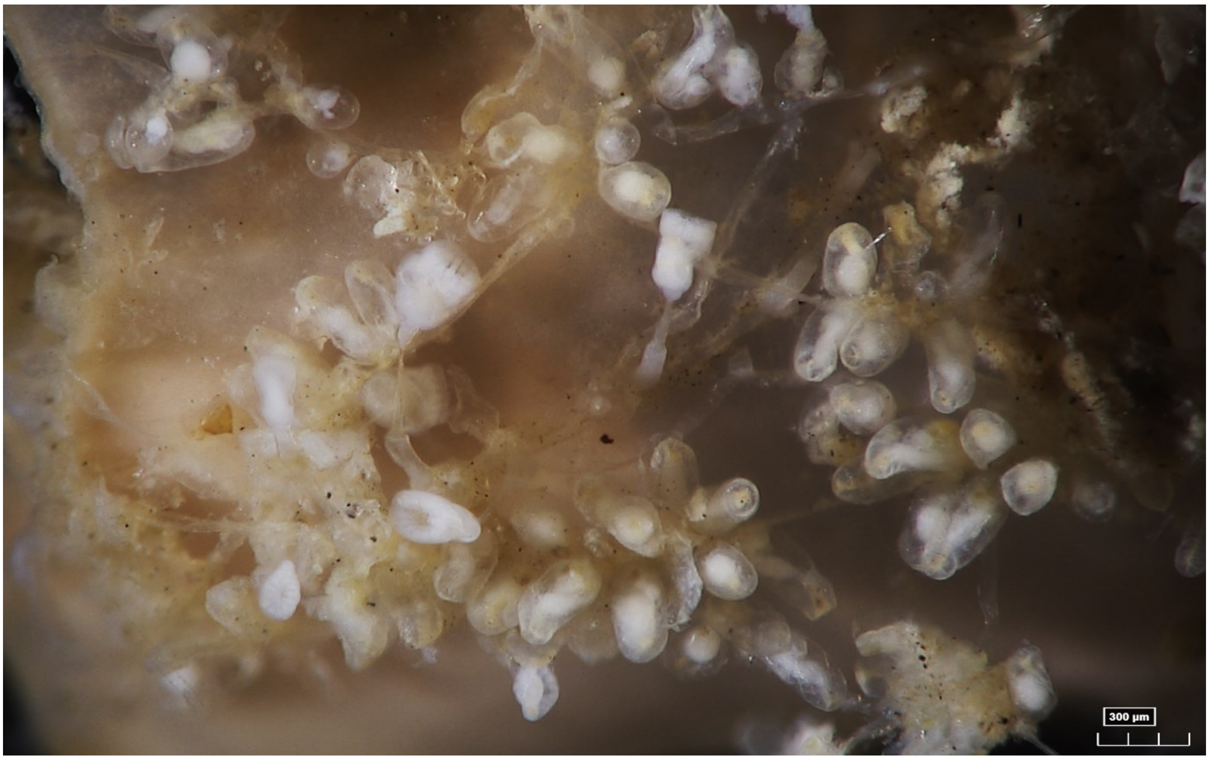

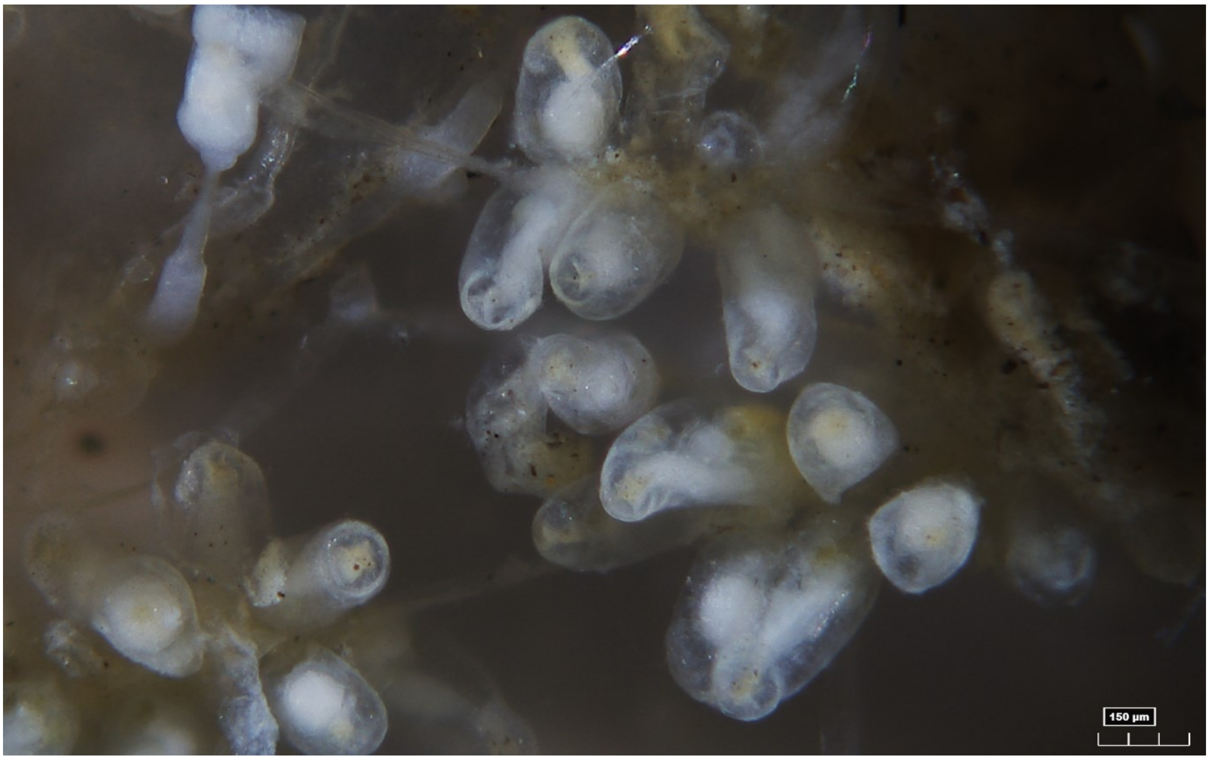

Vesicularia spinosa VIBE_ Vesi-FR22-66A

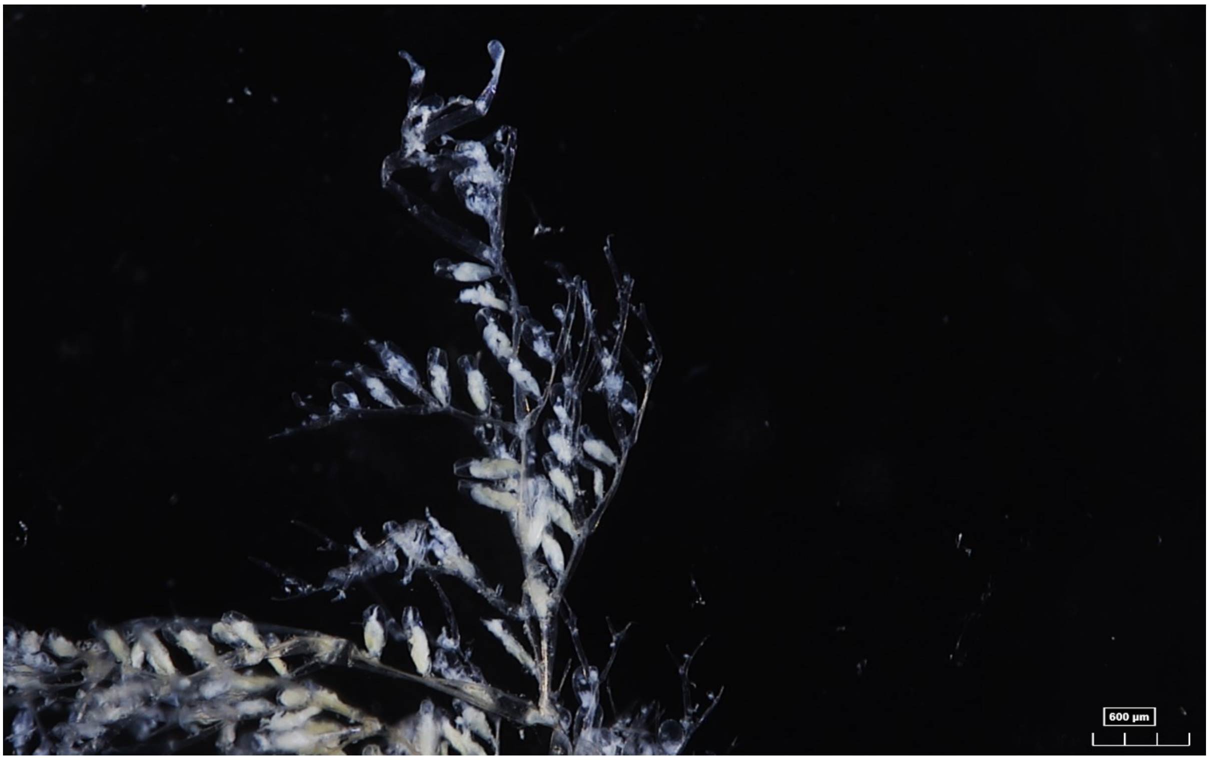

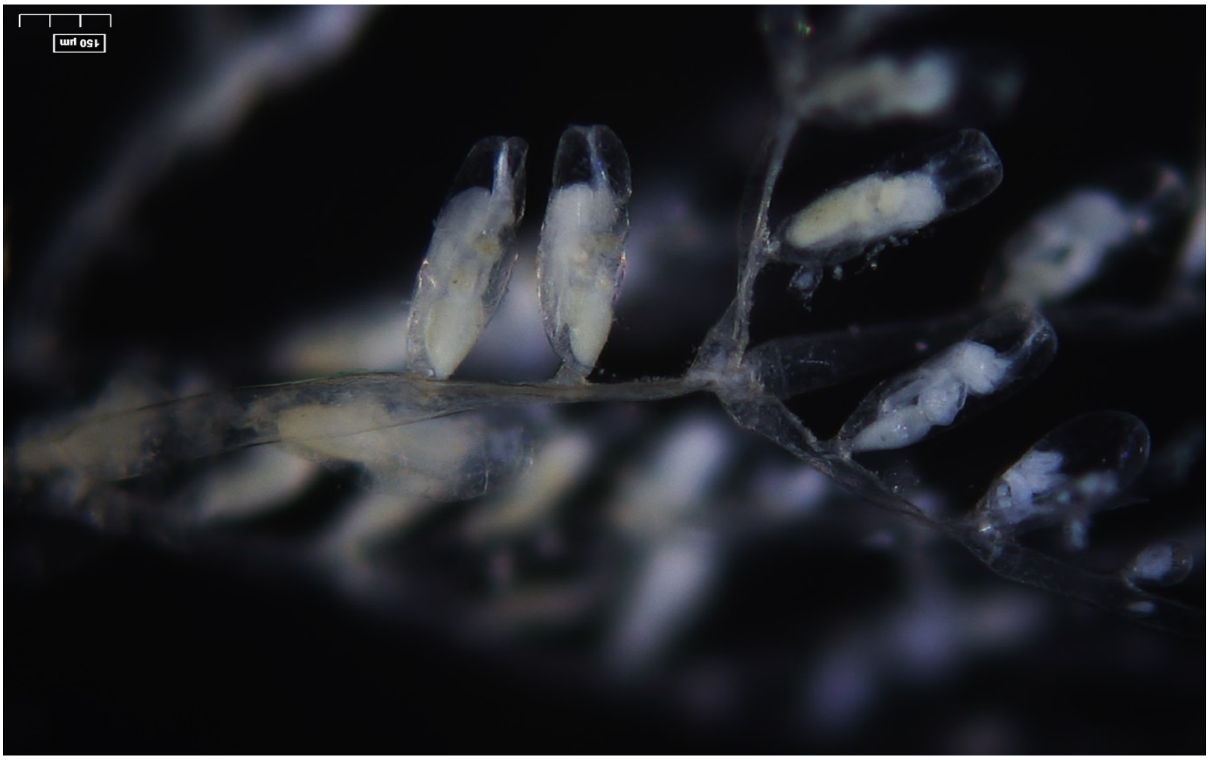

Supplement: Supplementary file 1 — Appendix S1. [file ECE3-14-e11276-s001.pdf]
